# *OsSYL2* *^AA^*, an allele identified by gene‐based association, increases style length in rice (*Oryza sativa* L.)

**DOI:** 10.1111/tpj.15013

**Published:** 2020-10-30

**Authors:** Xiaojing Dang, Yang Yang, Yuanqing Zhang, Xiangong Chen, Zhilan Fan, Qiangming Liu, Jie Ji, Dalu Li, Yanhui Li, Bingjie Fang, Zexu Wu, Erbao Liu, Xiaoxiao Hu, Shangshang Zhu, Dong She, Hui Wang, Yulong Li, Siqi Chen, Yufeng Wu, Delin Hong

**Affiliations:** ^1^ State Key Laboratory of Crop Genetics and Germplasm Enhancement Nanjing Agricultural University Nanjing 210095 China; ^2^ Rice Research Institute Guangdong Academy of Agricultural Sciences Guangzhou 510640 China; ^3^ Special Crop Research Institute Chongqing Academy of Agricultural Sciences Chongqing 402160 China; ^4^ School of Agriculture and Biology Shanghai Jiao Tong University Shanghai 200240 China; ^5^ Key Laboratory of Crop Germplasm of Zhejiang Province Institute of Crop Science Zhejiang University Hangzhou 310058 China

**Keywords:** favorable alleles, genome‐wide association study, hybrid rice seed production, natural variation, stigma characteristics

## Abstract

Stigma characteristics are important factors affecting the seed yield of hybrid rice per unit area. Natural variation of stigma characteristics has been reported in rice, but the genetic basis for this variation is largely unknown. We performed a genome‐wide association study on three stigma characteristics in six environments using 1.3 million single‐nucleotide polymorphism (SNPs) characterized in 353 diverse accessions of *Oryza sativa*. An abundance of phenotypic variation was present in the three stigma characteristics of these collections. We identified four significant SNPs associated with stigma length, 20 SNPs with style length (SYL), and 17 SNPs with the sum of stigma and style length, which were detected repeatedly in more than four environments. Of these SNPs, 28 were novel. We identified two causal gene loci for SYL, *OsSYL3* and *OsSYL2*; *OsSYL3* was co‐localized with the grain size gene *GS3*. The SYL of accessions carrying allele *OsSYL3^AA^* was significantly longer than that of those carrying allele *OsSYL3^CC^*. We also demonstrated that the outcrossing rate of female parents carrying allele *OsSYL2^AA^* increased by 5.71% compared with that of the isogenic line carrying allele *OsSYL2^CC^* in an F_1_ hybrid seed production field. The allele frequencies of *OsSYL3^AA^* and *OsSYL2^AA^* decreased gradually with an increase in latitude in the Northern Hemisphere. Our results should facilitate the improvement in stigma characteristics of parents of hybrid rice.

## Significance Statement

The utilization of heterosis in rice is an effective strategy to enhance the grain yield. However, this entails the production of F_1_ hybrid seeds every year. The yield of F_1_ hybrid seeds is affected by stigma characteristics. The cloning of *OsSYL2^AA^* filled a gap in the genetic study of style length and could be used for improving stigma characteristics to increase the yield of F_1_ seeds.

## INTRODUCTION

**Figure 1:**
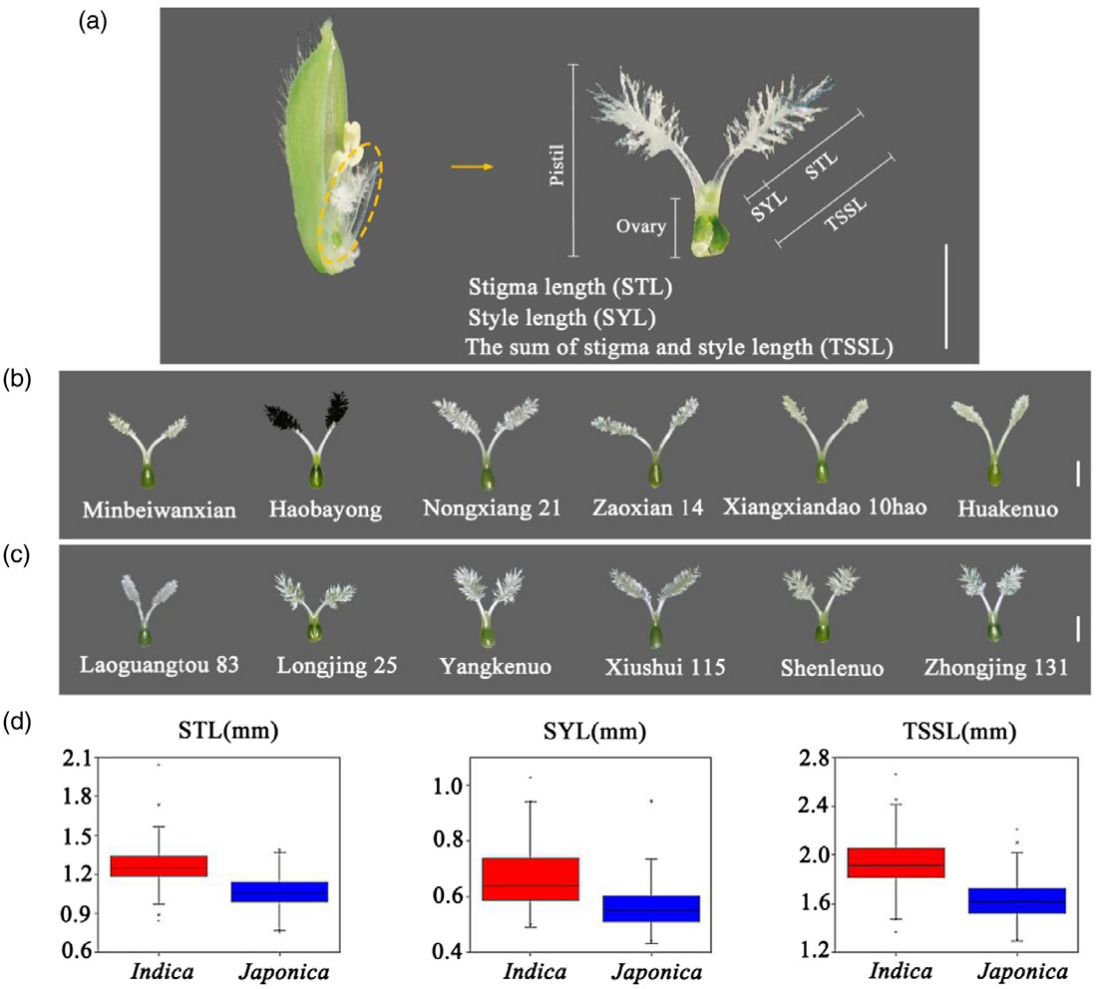
Scheme of rice stigma characteristics investigated, pistil morphology, and the phenotypic value distributions of three stigma characteristics in the *indica* and *japonica* rice subgroups. (a) Scheme of rice stigma characteristics investigated. Scale bar = 1 mm. (b) Stigma morphology of partial *indica* rice accessions. Scale bar = 1 mm. (c) Stigma morphology of partial *japonica* rice accessions. Scale bar = 1 mm. (d) Phenotypic value distributions of three stigma characteristics in the *indica* and *japonica* subgroups. The box edges represent the upper and lower quantiles, with the median value shown by the black line in the middle of each box. Vertical lines represent the data from the lowest quantile to the top quantile. Individuals falling outside the range of the whiskers are shown as asterisks.

Rice is cultivated on approximately 160 million hectares of land distributed across 117 countries, with an average annual yield of 4.4 tonnes per hectare (GRiSP (Global Rice Science Partnership), 2013), and feeds more than 3.5 billion people worldwide. As the human population increases and the area of arable land decreases, increasing the rice grain yield per unit area per unit time is a necessity. The utilization of F_1_ heterosis is an effective strategy to enhance rice grain yield; however, this entails the production of F_1_ hybrid seeds every year. The yield of F_1_ hybrid rice seeds is determined by the outcrossing seed setting rate for a given number of spikelets per unit area. The outcrossing seed setting rate in rice is mainly affected by the stigma exertion percentage, which is largely determined by stigma length (STL), style length (SYL), and the sum of stigma and style length (TSSL) (Figure 1a).

Stigma characteristics in rice are a complex quantitative trait controlled by multiple genes (Virmani and Athwal, 1974; Yu *et al*., 2003). In nature, the TSSL in *Oryza* ranges from 1.25 to 3.21 mm (Kato and Namai, 1987; Li *et al*., 2010; Marathi *et al*., 2015). Thirteen quantitative trait loci (QTLs) for TSSL have been identified, and are distributed on chromosomes 1, 2, 3, 4, 6, and 7 (Liu *et al*., 2015; Marathi *et al*., 2015; Dang *et al*., 2016). These QTLs explain phenotypic variance (*R* ^2^) of 2.9–20.0%; 9 of the 13 QTLs have an *R* ^2^ > 10%. The additive effect of QTL *qSSL‐3* on chromosome 3 is the largest and can increase TSSL by 9.2% (Li *et al*., 2010). Among the QTLs, one candidate gene, *Os03g0253400* (MSU ID *LOC_Os03g14850*) for controlling TSSL has also been identified (Liu *et al*., 2015).

The STL in *Oryza* ranges from 0.55 to 2.3 mm (Virmani and Athwal, 1973; Uga *et al*., 2010). To date, 32 QTLs have been detected for STL, including 1, 1, 12, 3, 3, 3, 2, 0, 3, 2, 0, and 2 on chromosomes 1 to 12, respectively (Uga *et al*., 2003, 2010; Yan *et al*., 2009; Li *et al*., 2010; Marathi *et al*., 2015; Dang *et al*., 2016). The *R* ^2^ of these QTLs ranges from 2.2% to 30.8%; 10 of the 32 QTLs have an *R* ^2^ > 10%. The additive effect of QTL *qSTL‐4* on chromosome 4 is the largest and increases STL by 10.6% (Uga *et al*., 2003). However, no gene governing the STL has been isolated.

The SYL in *Oryza* ranges from 0.4 to 1.76 mm (Li *et al*., 2010; Dang *et al*., 2016). In total, 23 QTLs for SYL have been detected, and are located on chromosomes 1, 2, 3, 4, 6, 7, 10, and 11. These QTLs have a wide *R* ^2^ of 3.1–55.4%, in which 12 QTLs have an *R* ^2^ > 10% (Uga *et al*., 2003, 2010; Li *et al*., 2010; Marathi *et al*., 2015; Dang *et al*., 2016; Zhou *et al*., 2017). The additive effect of QTL *qSYL3* on chromosome 3 is the largest and can increase SYL by 10.2% (Uga *et al*., 2010). However, no gene governing the SYL has been isolated.

Hybrid rice has been cultivated commercially in China for 43 years. In the past 20 years, the average seed production yield has remained stagnant at 2.25 t ha^−1^. Increasing the TSSL, STL, and SYL to improve the rate of stigma exertion will be beneficial to increase the seed production yield of hybrid rice. In this study, we investigated the TSSL, STL, and SYL of 353 rice accessions at two locations over 3 years. By combining the TSSL, STL, and SYL with single‐nucleotide polymorphism (SNP) data, we performed a genome‐wide association study (GWAS) and identified significant SNP loci. Further, using the method of gene‐based association we identified a novel causative gene *OsSYL2* for SYL. The function of *OsSYL2^AA^* was validated by transgenic complementation testing and evaluation of F_1_ seed production in the paddy field. These results filled in the gap from gene cloning and functional analysis of stigma characteristics. The gene‐based association method used in this study resulted in the ability to access the causative gene/SNP for *OsSYL2*. The favorable alleles identified herein could facilitate improvement in stigma characteristics of the parents of hybrid rice.

## RESULTS

### Phenotypic variation of the stigma characteristics in the natural population

According to the definitions of the three stigma characteristics (STL, SYL, and TSSL) (Figure 1a), the phenotypic values of STL, SYL, and TSSL were investigated in 353 accessions containing *indica* and *japonica* subspecies (Table S1 in the online Supporting Information). Partial photos of pistils are shown in Figure 1(b) and (c). The distributions of global averages over the six environments for STL, SYL, and TSSL in each subspecies are shown in Figure 1(d). In the 353 accessions, the mean value of STL was calculated per environment, ranging from 1.16 ± 0.16 mm to 1.19 ± 0.18 mm, with coefficients of variation (CV) across the six environments from 13.79% to 15.38% (Figure S1, Table S2); the mean value of SYL was calculated per environment, ranging from 0.60 ± 0.11 mm to 0.63 ± 0.43 mm, with CV from 18.03% to 19.35% (Figure S1, Table S2); and the mean value of TSSL was calculated per environment, ranging from 1.77 ± 0.22 mm to 1.80 ± 0.24 mm (Figure S1, Table S2), with CV from 12.43% to 13.33%. The average CV of TSSL across the six environments was 12.96%, which was lower than that for STL (14.67%) and SYL (18.91%) (Table S2). These results indicate that there was abundant phenotypic variation in the natural populations studied. Compared with *indica* rice, the *japonica* rice population had lower values for the three stigma characteristics (Figure 1d). The results of joint analysis of variance for each trait showed significant differences among genotypes, but not among the environments and the interactions of genotypes with environments (Table S3), indicating that the environment had little effect on the stigma characteristics and the abundant phenotypic variation of the stigma characteristics was mainly attributable to variation in genotype.

### Genomic variation at the SNP level in the 353 rice accessions

A total of 1.90 billion paired‐end reads of length 150 bp were obtained from 353 rice accessions resequenced using the Illumina resequencing platform, with an average coverage depth of 4.36‐fold for each accession. After mapping against the Nipponbare reference genome sequence, we identified 1 326 094 SNPs after excluding the sites with missing data for more than 18% of all the accessions. We observed 463 740 SNPs in the genic regions: 48 054 synonymous, 52 283 non‐synonymous, 270 622 intronic, 62 181 3′‐untranslated region (UTR), and 30 600 5′‐UTR SNPs.

Based on the SNP data, the SNP density and nucleotide diversity (π) showed great variation along chromosomes (Figure S2a,b). Some chromosome regions of the *indica* and *japonica* groups had high values of *F*_ST_ (the population differentiation index, also called the fixation index; *F*_ST _> 0.5), including a total length of 1.6 Mb in *japonica* rice and 0.7 Mb in *indica* rice, indicating that they contain gene loci that may be involved in geographic adaptation (Figure S2c). The *F*_ST_ value between the *indica* group and *japonica* group was 0.52. These results suggest a high level of genetic differentiation between *indica* and *japonica* rice. Within cultivars, the π level of *indica* rice (6.75 × 10^−4^) was higher than that of *japonica* rice (4.34 × 10^−4^). These results indicate the presence of rich genomic diversity at the SNP level among the 353 accessions.

### Population genetic structure and linkage disequilibrium

The Bayesian model‐based population structure analysis provided evidence that there is a significant population structure in the 353 rice accessions. As the log‐likelihood values increased with an increase in the *K* value (Figure S3a), we used the Δ*K* value to determine a suitable number of subgroups, *K*. The Δ*K* value was highest at *K* = 2 (Figure S3b). Therefore, the entire population was divided into two subgroups, named the *indica* subgroup and *japonica* subgroup (Figure S3c). We defined an accession as a non‐admixed accession when its *Q* value was larger than 0.85. The number of non‐admixed accessions in the *indica* and *japonica* subgroups was 166 and 165, respectively, and the remaining 22 accessions were assigned to the admixture group. The results of the population structure analysis based on the Bayesian model were further confirmed by principal component analysis (Figure S3d) and neighbor‐joining tree analysis based on Nei’s genetic distances (Nei *et al*., 1983) (Figures S3e and S4).

We further analyzed linkage disequilibrium (LD; expressed as *r* ^2^) in the whole rice population, the *indica* subgroup, and the *japonica* subgroup. The extent of LD was measured by the chromosomal distance at which *r* ^2^ decreased to half its maximum value. The LD decay distances in the whole rice population, the *indica* subgroup, and the *japonica* subgroup were 177 kb (*r* ^2^ = 0.26), 57 kb (*r* ^2^ = 0.26), and 214 kb (*r* ^2^ = 0.28), respectively (Figure S3f). We believe that the faster LD decay in the *indica* rice subgroup than in the *japonica* rice subgroup may be attributable to the frequent artificial crossing of *indica* rice during the breeding process, because two to three crops of *indica* rice are grown per year in areas where the latitude is below 30° N, whereas only one crop of *japonica* rice is grown per year in area were the latitude is above 30° N. The LD decay distance of the *indica* rice subgroup was slightly lower than that reported by Huang *et al*. (2010) (123 kb). The LD decay distance of the *japonica* rice subgroup was slightly higher than that reported by Huang *et al*. (2010) (167 kb).

### Genome‐wide association mapping

Using the mixed linear model with correction of kinship bias, we conducted GWAS between stigma characteristics and SNPs [minimum allele frequency (MAF) > 0.05] in the 353 rice accessions. In this population, 41 significantly associated SNP loci were detected in the 34 LD regions (Table 1). These SNPs were located on chromosomes 1–4, 6, 7, 9, 10, and 12. In addition, these significantly associated SNP loci were repeatedly detected in at least four environments, which showed that the SNP–trait associations were stable (Table S4).

**Table 1:** The summary of single‐nucleotide polymorphisms (SNPs) significantly associated with stigma characteristics

Characteristics	Chromosome	SNP site	Local LD	Allele	Range −log_10_ (*P*)	Range *R* ^2^ (%)	Environment
STL	2	14 511 886	14 307 211–14 761 529	A/T	5.70–5.90	3.88–4.06	E1, E4–E6
	3	22 114 062	21 877 844–22 363 223	C/T	5.65–6.18	3.80–4.20	E1, E4–E6
	7	24 396 164	24 294 065–24 763 963	T/C	6.26–7.41	4.26–5.20	E2, E4–E6
	10	14 401 388	14 177 572–14 650 408	T/C	5.68–7.05	3.85–4.93	E1, E2, E4–E6
SYL	1	11 281 296	11 038 247–11 507 022	C/T	5.66–6.75	3.83–4.71	E2, E3, E5, E6
	1	25 139 654	24 889 863–25 389 637	T/C	5.58–6.54	3.75–4.45	E2–E6
	1	31 869 936	31 672 589–32 049 317	A/G	5.56–7.63	3.73–5.43	E2, E3, E5, E6
	1	34 395 535	34 145 615–34 645 322	G/A	6.20–7.48	4.20–5.29	E1, E3–E6
	2	30 510 492	30 465 900–30 660 551	G/A	6.05–7.09	4.15–4.96	E1–E6
	2	30 512 579	30 458 941–30 697 061	A/G	5.56–7.29	3.73–5.13	E1, E3–E6
	2	30 596 777	30 465 900–30 710 907	T/G	5.79–8.06	3.93–5.85	E1‐E6
	2	30 606 084	30 465 056–30 786 393	C/T	5.82–7.39	4.01–5.20	E1–E3, E5, E6
	2	30 620 061	30 465 900–30 786 312	G/T	5.53–6.77	3.71–4.72	E1, E3–E6
	3	16 690 429	16 663 167–16 915 445	G/A	5.62–7.26	3.78–5.12	E1–E3, E6
	3	16 692 834	16 663 167–16 915 445	A/G	5.53–6.79	3.71–4.72	E2, E3, E5, E6
	3	16708 049	16 663 167–16 915 445	A/G	5.62–5.86	3.78–4.03	E1–E5
	3	16 733 441	16 663 167–16 915 445	C/A	5.88–7.91	4.05–5.75	E2–E5
	3	16 881 568	16 663 167–16 915 445	G/T	5.78–7.02	3.93–4.91	E1–E3, E5, E6
	3	30 732 321	30 731 129–30 825 725	T/C	5.65–6.24	3.80–4.22	E2–E5
	4	9 290 646	9 046 520–9 509 221	A/G	5.88–7.64	4.05–5.43	E2, E3, E5, E6
	6	1 153 212	1 153 212–1 153 212	A/G	5.574–7.88	3.92–5.71	E2–E6
	6	26 598 751	26 398 744–26 848 690	C/A	5.74–6.06	3.92–4.15	E3–E6
	9	17 486 934	17 244 424–17 736 495	C/T	5.72–7.88	3.90–5.71	E1–E3, E6
	10	3 094 153	3 044 455–3 127 541	T/A	5.97–7.59	4.07–5.38	E2, E3, E5, E6
TSSL	3	16 183 113	16 114 203–16 301 618	A/G	6.24–7.41	4.22–5.19	E1–E6
	3	16 184 264	16 148 731–16 321 816	A/G	5.52–7.17	3.71–5.01	E1–E6
	3	16 197 239	16 012 266–16 321 816	A/G	5.65–7.33	3.79–5.19	E1, E3–E6
	3	16 197 256	16 012 266–16 321 816	T/C	5.62–6.82	3.78–4.79	E1, E3–E6
	3	16 202 578	16 088 681–16 321 816	C/T	5.88–7.39	4.05–5.24	E1–E6
	3	16 256 300	16 183 113–16 321 816	A/G	5.62–6.49	3.78–4.44	E1, E2, E4–E6
	3	16 258 184	16 183 113–16 321 816	T/G	5.64–7.27	3.79–5.11	E3–E6
	3	16 686 373	16 663 167–16 931 334	G/A	5.50–7.27	3.68–5.11	E1–E6
	3	16 691 998	16 663 167–16 940 264	T/G	5.89–7.39	4.05–5.24	E1, E2, E4–E6
	3	16 720 463	16 663 167–16 970 433	T/A	5.51–5.85	3.70–4.03	E1, E2, E4, E5
	3	16 733 441	16 663 167–16 970 433	C/A	5.60–7.53	3.74–5.33	E1, E4–E6
	3	16 878 104	16 663 167–17 020 970	G/A	5.55–6.84	3.72–4.80	E1–E4, E6
	3	17 019 509	16 777 275–17 020 970	G/A	5.89–6.91	4.06–4.83	E1, E2, E5, E6
	6	16 692 729	16 692 729–16 692 729	C/A	6.25–7.53	4.26–5.33	E2, E3, E5, E6
	9	20 459 617	20 210 383–20 553 707	A/G	5.69–6.70	3.85–4.67	E1, E2, E5, E6
	12	16 580 802	16 545 017–16 659 883	A/G	5.82–7.18	4.01–5.01	E2, E3, E5, E6
	12	16 585 045	16 482 437–16 680 222	T/C	6.15–7.32	4.18–5.19	E2, E3, E5, E6

LD, linkage disequilibrium; STL, stigma length; SYL, style length; TSSL, the sum of stigma and style length. The value of −log_10_ (*P*) indicates the significance levels and PVE indicates the percentage of phenotypic variation explained by each SNP. Environments: E1, environment 1, Nanjing 2014; E2, environment 2, Nanjing 2015; E3, environment 3, Nanjing 2016; E4, environment 4, Yuanyang 2014; E5, environment 5, Yuanyang 2015; E6, environment 6, Yuanyang 2016.

For STL, four significant SNP loci were identified on chromosomes 2, 3, 7, and 10, contributing 3.80–5.20% of the *R* ^2^ (Table 1, Figure S5). The SNP locus on chromosome 10 was detected in five environments (Figure S5, Table S4).

For SYL, 20 significant SNP loci were identified on chromosomes 1–4, 6, 9, and 10, contributing 3.71–5.85% of the *R* ^2^ (Table 1, Figure S6). Two of these SNP loci were detected in six environments and eight loci were detected in five environments (Figure S6, Table S4).

For TSSL, 17 significant SNP loci were identified on chromosomes 3, 6, 9, and 12, with *R* ^2^ from 3.68% to 5.33% (Table 1, Figure S7). Four of these SNP loci were detected across six environments and five loci were detected in five environments (Figure S7, Table S4). The SNP locus (16 733 441 bp) was associated with both SYL and TSSL traits simultaneously. Next, we analyzed the major SNP loci relevant to SYL with a significant peak, present in chromosomes 3 and 2, respectively.

### **Allele** ****OsSYL3^AA^**** **increases style length**

**Figure 2:**
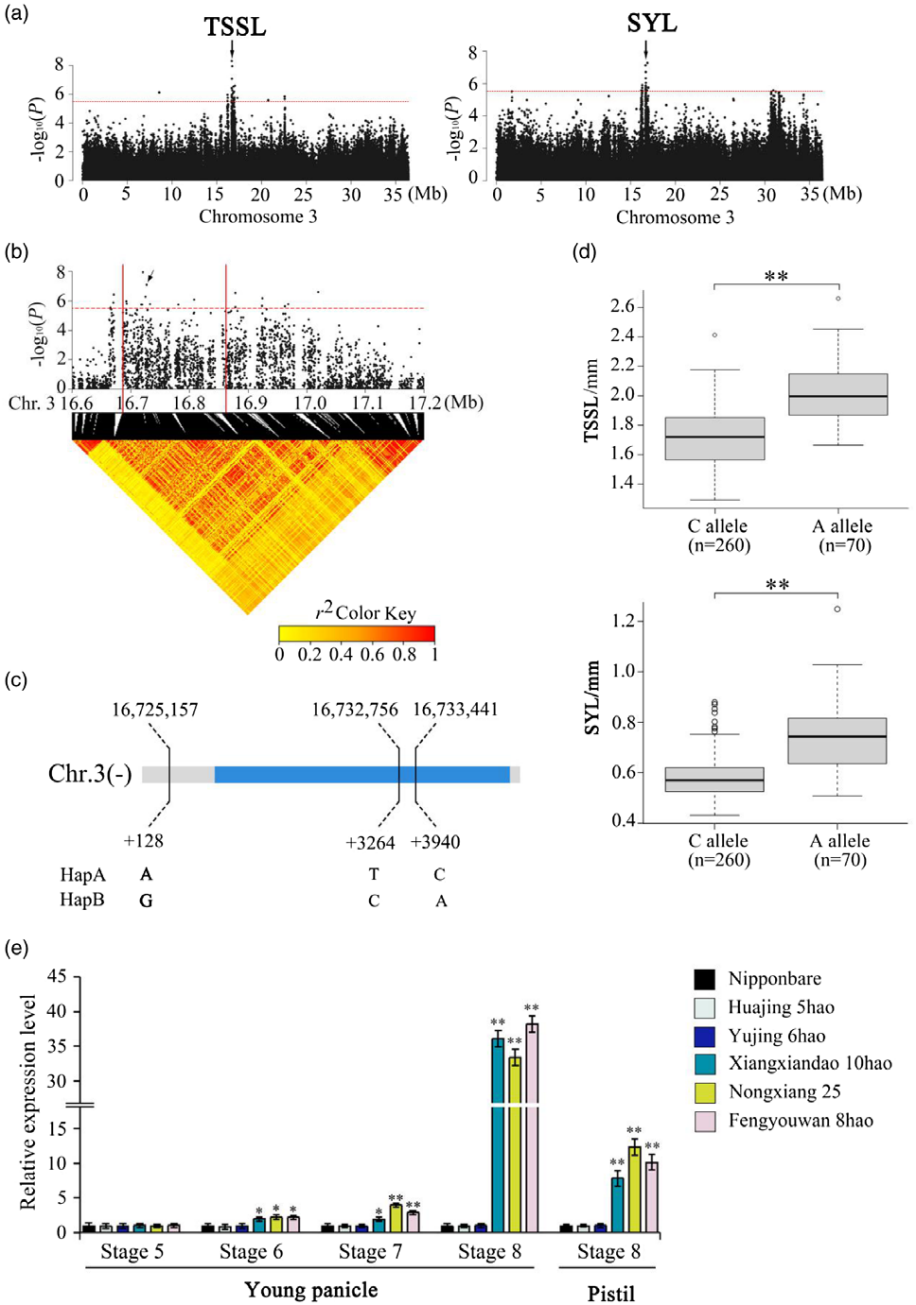
Genome‐wide association study for the sum of stigma and style length (TSSL) and style length (SYL) and identification of the causal gene *OsSYL3* (*Os03g0407400*). (a) Manhattan plots for TSSL and SYL. Arrowheads indicate the position of strong peaks. Dashed lines represent significance thresholds (−log_10_ *P* = 5.5). (b) Local Manhattan plot (top) and linkage disequilibrium heatmap (bottom). The arrow indicates the position of nucleotide variation in *Os03g0407400*. The candidate region lies between the red solid lines. (c) Single‐nucleotide polymorphisms in *OsSYL3* cDNA between HapA and HapB. (d) Box plots for TSSL and SYL traits for the two alleles (*n* = 260 versus 70). Central lines indicate the median value, box edges represent the upper and lower quantiles, whiskers extend to 1.5× the interquantile range, and dots represent outliers (**P < 0.01, two‐tailed Welch’s *t*‐test). (e) Relative expression of *Os03g0407400* in young panicles at development stages 5–8 and pistils at stage 8 from the three accessions (Nipponbare, Huajing 5hao, and Yujing 6hao) with a short TSSL and SYL and the three accessions (Xiangxiandao 6hao, Nongxiang 25, and Fengyouwan 8hao) with a long TSSL and SYL, determined by quantitative RT‐PCR (**P < 0.01, *P < 0.05, two‐tailed Welch’s *t*‐test). Data are presented as means ± SE; *n* = 3 independent biological replicates.

For the association signal (chromosome 3: 16 733 441) in the 16.69–16.87 Mb region, there were 15 candidate genes associated with the significant SNP loci (Figure 2a,b). In this region, 9 of the 15 genes contained non‐synonymous SNPs (Tables S5 and S6). Only one non‐synonymous SNP in *Os03g0407400* was found to be significantly associated with the SYL and TSSL traits (−log_10_ *P* ≥ 5.5). Hereafter, the gene *Os03g0407400* is referred to as *OsSYL3*. *OsSYL3* was classified into two haplotypes based on three SNPs in its cDNA containing one SNP in an UTR, one in an intron and one missense SNP in the coding region (Figure 2c). This missense SNP causes a base change from base C to base A at nucleotide (nt) 165 in the coding sequence, resulting in an amino acid change from cysteine (C) to a stop codon. The average TSSL and SYL values of 70 accessions carrying the allele *OsSYL3^AA^* were 1.97 ± 0.23 mm and 0.76 ± 0.28 mm, respectively. The average TSSL and SYL values of 260 accessions carrying the allele *OsSYL3^CC^* were 1.72 ± 0.16 mm and 0.58 ± 0.25 mm, respectively. The differences in TSSL and SYL values between the *OsSYL3^AA^* and *OsSYL3^CC^* genotypes were highly significant (Welch’s *t*‐test, *P* = 2.20 × 10^–16^) (Figure 2d).

The quantitative (q) RT‐PCR results showed that the expression of *OsSYL3^AA^* was higher than that of *OsSYL3^CC^* in young panicles at differentiation stages 6, 7, and 8, but no significant difference was found at stage 5 (Figures 2e and S8). The expression of *OsSYL3^AA^* was the highest in young panicles at stage 8, of the four stages investigated, whereas the expression of *OsSYL3^CC^* did not peak in panicles among the four stages. We further performed qRT‐PCR analysis of pistils at stage 8, sampled from three accessions (Xiangxiandao 10hao, Nongxiang 25, and Fengyouwan 8hao) with longer TSSL and SYL and three accessions (Nipponbare, Huajing 5hao, and Yujing 6hao) with shorter TSSL and SYL. The results showed that the expression of *OsSYL3^AA^* in each of the three accessions with longer TSSL and SYL was significantly higher than that of *OsSYL3^CC^* in each of the three accessions with shorter TSSL and SYL. By searching the China Rice Data Center (http://www.ricedata.cn/gene/) websites, we found that the gene locus *Os03g0407400* was identical to *Grain Size 3* (*GS3*) reported by Fan *et al*. (2006). The allele *GS3^AA^* increased grain length and STL (Takano‐Kai *et al*., 2009, 2011). Therefore, we will not study the function of *OsSYL3^AA^* further.

### Introduction of the allele *OsSYL2^AA^* increases SYL

**Figure 3:**
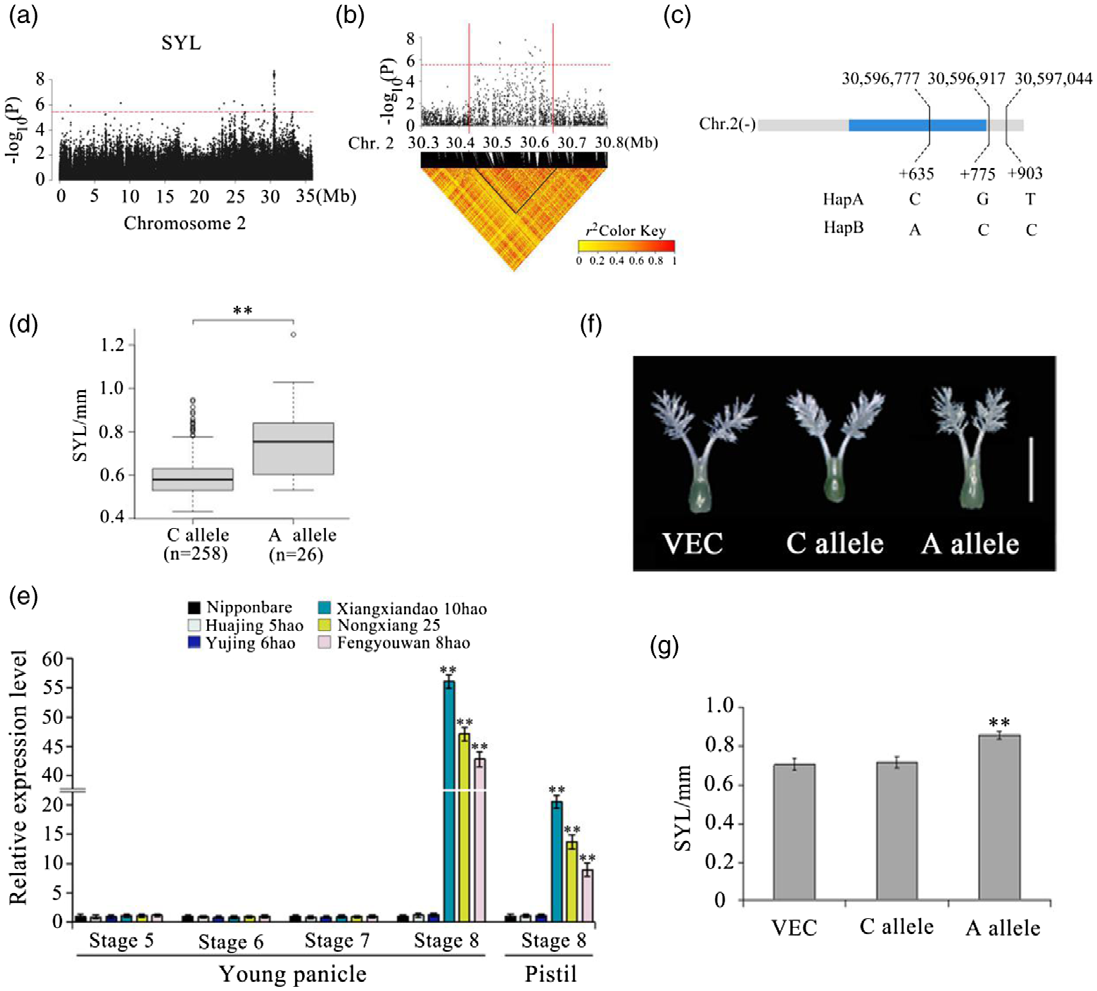
Genome‐wide association study for style length (SYL) and identification of the causal gene *OsSYL2* (*Os02g0733900*). (a) The Manhattan plot of chromosome 2 for SYL. In the Manhattan plots of chromosome 2, horizontal dashed lines indicate the significance threshold (−log_10_ *P* = 5.5). Arrows indicate the position of strong peaks for SYL. (b) Local Manhattan plot (top) and linkage disequilibrium heatmap (bottom). The arrow indicates the position of nucleotide variation in *Os02g0733900*. The candidate region lies between the red solid lines. (c) Single‐nucleotide polymorphisms in *OsSYL2* cDNA between HapA and HapB. (d) Boxplots for SYL based on the alleles for *Os02g0733900*. Central line, median; box limits, upper and lower quantiles; whiskers, 1.5× the interquantile range; dots, outliers. Differences between alleles were statistically analyzed using Welch’s *t*‐test (**P < 0.01). (e) Relative expression of *Os02g0733900* in young panicles at development stages 5–8 and pistils at stage 8 from the three accessions (Nipponbare, Huajing 5hao, and Yujing 6hao) with a short SYL and the three accessions (Xiangxiandao 6hao, Nongxiang 25, and Fengyouwan 8hao) with a long SYL, determined by quantitative RT‐PCR (**P < 0.01, two‐tailed Welch’s *t*‐test). Data are presented as means ± SE; *n* = 3 independent biological replicates. (f) Images of pistil of transgenic plants transformed with the empty vector (VEC), C allele, and A allele. Scale bar = 1 mm. (g) Style length of transgenic plants. Data are presented as means ± SE (*n* = 20).

For SNPs associated with SYL, a significant peak appeared in chromosome 2 and 33 candidate genes were detected in the candidate region of 30.45–30.65 Mb (200 kb) (Figure 3a,b). For SNPs in this candidate region, 10 of the 33 genes contain non‐synonymous SNPs (Tables S7 and S8). However, only one non‐synonymous SNP was significantly associated with SYL (−log_10_ *P* ≥ 5.5); it was located within the gene locus *Os02g0733900* (MSU ID *LOC_Os02g50110*). Hereafter, gene *Os02g0733900* is referred to as *OsSYL2*. The full length of *OsSYL2* is 602 bp, including one exon and no introns. Gene *OsSYL2* encodes an 80‐amino‐acid protein. No putative conserved domains have been detected for *OsSYL2*. *OsSYL2* was classified into two haplotypes based on three SNPs in its cDNA containing one missense SNP in the coding region and two SNPs in the UTR (Figure 3c). The missense SNP causes a base change from base C to base A at nt 126 in the coding sequence, which results in an amino acid change from histidine (H) to asparagine (N) at amino acid 42 (Figure 3c). The average SYL value of 26 accessions carrying the allele *OsSYL2^AA^* was 0.77 ± 0.18 mm. The average SYL value of 258 accessions carrying the allele *OsSYL2^CC^* was 0.58 ± 0.21 mm. The difference in SYL value between the *OsSYL2^AA^* and *OsSYL2^CC^* genotypes was highly significant (Welch’s *t*‐test, *P* = 9.06 × 10^–5^) (Figure 3d).

The qRT‐PCR results showed that the expression of *OsSYL2^AA^* was higher than that of *OsSYL2^CC^* in young panicles at differentiation stage 8, but no significant differences were found at stages 5, 6, and 7 (Figure 3e). We further performed qRT‐PCR analysis using pistils at stage 8, sampled from the aforementioned six accessions, and found that the expression of *OsSYL2^AA^* in each of the three accessions with longer SYL was significantly higher than that of *OsSYL2^CC^* in each of the three accessions with shorter SYL. These results suggested that enhanced expression of *OsSYL2^AA^* might increase SYL.

Based on the results of GWAS, no SNP loci found in the promoter region of *OsSYL2* were associated with SYL. Further, we searched the website of promoter functional elements (http://bioinformatics.psb.ugent.be/webtools/plantcare/html/#opennewwindow), but found no SNP loci in the *cis*‐element regulatory region. We speculated that phenotypic variation between the accessions with the AA allele and those with the CC allele was caused by SNP loci in the coding sequence region. Thus, we conducted transformation of *OsSYL2* gene to confirm our speculation.

We introduced the genome sequence of the allele *OsSYL2^AA^* and empty vector into Nipponbare, respectively. Compared with the plants of Nipponbare genome, plants transformed with the allele *OsSYL2^AA^* had a longer SYL whereas those transformed with the empty vector showed no phenotypic change (Figure 3f,g). These results showed that *OsSYL2* was the causal gene for SYL on chromosome 2.

**Figure 4:**
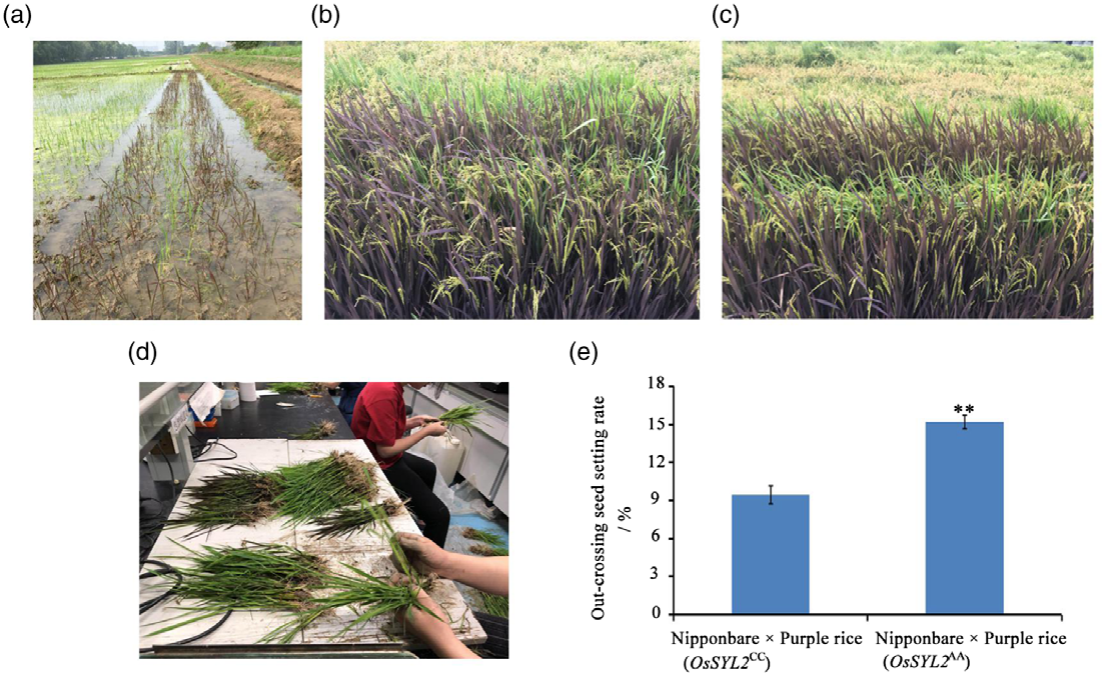
F_1_ seed production field and investigation of outcrossing rate. (a) Planting pattern of male and female parent for F_1_ seed production. (b) Seed production in the paddy rice field for combination of Nipponbare (*OsSYL2^CC^*) × purple rice at filling stage. (c) Seed production in the paddy rice field for combination of Nipponbare (*OsSYL2^AA^*) × purple rice at filling stage. (d) Investigation of the frequency of purple seedlings. (e) Histogram of the outcrossing seed setting rate. Significance was determined using a *t‐*test (**P < 0.01).

To further evaluate the potential of the *OsSYL2^AA^* allele in hybrid rice seed production, we performed a field experiment using the two combinations, Nipponbare (*OsSYL2^CC^*) × purple rice accession and Nipponbare (*OsSYL2^AA^*) × purple rice accession. The potential of *OsSYL2^AA^* for hybrid rice seed production was evaluated by calculating the percentage of purple seedlings in the germination experiment with the two F_1_ populations. After investigating the frequency of purple seedlings, we found that in the combination of Nipponbare (*OsSYL2^CC^*) × purple rice, the percentage of purple seedlings, which represents the outcrossing seed setting rate, was 9.47% (Figure 4). In the combination of Nipponbare (*OsSYL2^AA^*) × purple rice, the outcrossing seed setting rate was 15.18%, an extra 5.71% compared with the former combination.

### Allele frequency distribution and stigma characteristic performance

**Figure 5:**
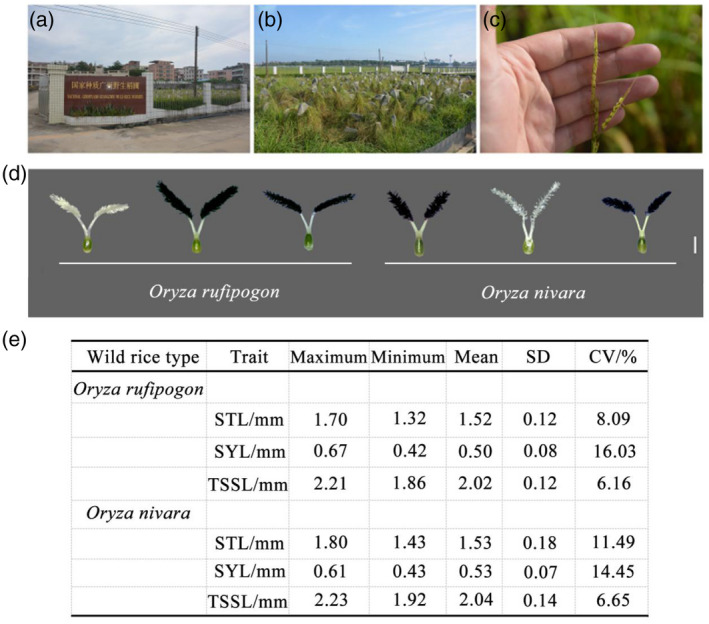
Field performance of *Oryza rufipogon* at filling stage and stigma morphology. (a) National Germplasm Guangzhou Wild Rice Nursery. (b) Field performance of *O*. *rufipogon* at filling stage. (c) Flowering spikelet of *O*. *rufipogon*. (d) Stigma morphology of *O*. *rufipogon* and *Oryza* *nivara*. Scale bar = 1 mm. (e) Basic statistics of the three stigma characteristics of *O*. *rufipogon*.

To elucidate the allele types of *OsSYL2* and *OsSYL3* loci in *Oryza* *rufipogon* and *Oryza nivara*, we sequenced eight accessions of *O*. *rufipogon* and four accessions of *O*. *nivara* at 5.64‐fold coverage. The information for 12 wild rice accessions is presented in Figure 5 and Table S9. The sequencing results showed that the alleles of both *OsSYL2* and *OsSYL3* loci were CC in both *O*. *rufipogon* and *O*. *nivara,* which was similar to *OsSYL2* and *OsSYL3* loci in *japonica* rice. We investigated the stigma characteristics of wild rice and found that STL was longer than SYL. The average SYL of *japonica* rice was 0.62 ± 0.12 mm, which was not significantly different from that of *O*. *rufipogon* (0.50 ± 0.08 mm) and *O*. *nivara* (0.53 ± 0.07 mm) (Figure 5). Therefore, we defined the CC alleles as wild type and the others as mutant alleles.

We investigated the regional differentiation of diverse alleles on *OsSYL2* and *OsSYL3* gene loci. The *OsSYL2^AA^* mutant allele (longer SYL) was found to be mainly distributed in accessions collected from low‐latitude regions, such as southern and central China, which is consistent with the grain length distribution of accessions collected in these areas (Figure S9). A similar situation was observed for *OsSYL3*, in which the mutated allele *OsSYL3^AA^* was mainly distributed in accessions collected from southern China and southeastern Asia (Figure S9). These results suggested that the longer SYL accession with a mutated allele was naturally selected during the process of domesticating *indica* rice and that the shorter SYL accession with a wild‐type allele was naturally retained during the domestication of *japonica* rice.

## DISCUSSION

In this study we investigated the phenotypic data for three stigma characteristics in 353 rice accessions and identified a rich variety of phenotypic variances. The CV for the three traits ranged from 12.43% (TSSL in E4) to 19.35% (SYL in E2, E3, and E6) (Table S2). The results of a joint variance analysis showed that variations in these three stigma characteristics were the main contribution to diverse genotypes, and no interactions between genotypes and environments were detected. These facts mentioned above provide a basis for the discovery of the favorable alleles of stigma characteristics. We also investigated the grain length of 353 rice accessions and calculated the Pearson’s correlation coefficients (Table S9). The results showed that TSSL and SYL are positively correlated with grain length, which is consistent with the results reported by Virmani and Athwal (1973) and Zhou *et al*. (2017). In the comparison of stigma characteristics between cultivated rice plants and the wild rice plants *O*.*rufipogon* and *O*.*nivara*, we found that TSSL in the wild rice plants was longer than that in the cultivated rice plants (Figure 5, Table S2), which provided a partial explanation for the higher outcrossing rate of wild rice than the cultivated rice plants. Therefore, increasing STL or SYL, or both, to lengthen TSSL is an effective strategy to enhance the outcrossing rate in F_1_ hybrid rice seed production.

We detected 41 SNP loci that were significantly associated with stigma characteristics; these were located in 34 LD regions (Table 1). Based on the information in the Gramene website (http://www.gramene.org/markers/) and the China Rice Data Center database (http://www.ricedata.cn/gene/), local LD regions harboring the 13 associated SNP sites were overlapped with the flanking regions of two QTLs (*qSYL3* and *qSYL6*) and two genes (*GL7* and *GS3*) reported previously (Uga *et al*., 2003; Fan *et al*., 2006; Wang *et al*., 2015; Zhou *et al*., 2017) (Table S10), and the remaining 28 associated SNP loci were newly identified in this study.

We identified two GWAS signals significantly associated with SYL to nearly single‐gene resolution. Gene *OsSYL3* coincided with the location of the grain size gene *GS3*. Takano‐Kai *et al*. (2011) reported that *GS3* could also increase STL. Zhou *et al*. (2017) confirmed that *GS3* had a large effect on SYL and no significant effect on STL. In the present study we further confirmed that *OsSYL3* (*GS3*) could regulate SYL. The allele *OsSYL3^AA^* increased SYL and the allele *OsSYL3^CC^* decreased SYL.

Gene *OsSYL2* is a gene newly identified in this study. The full length of *OsSYL2* is 602 bp, including one exon and no introns. Gene *OsSYL2* encodes an 80‐amino‐acid protein. We have demonstrated that a base C‐to‐A non‐synonymous mutation at nt 126 in the coding sequence of *OsSYL2* caused the long SYL phenotype by qRT‐PCR, the complementation test and evaluation of F_1_ seed production potential in the paddy field. By searching the STRING website, we found that 10 proteins interacted with *OsSYL2* (http://string‐db.org/cgi/network.pl?taskId=jf8rY202mAHU) (Figure S10). Of these, five are jasmonate ZIM‐domain (JAZ) proteins participating in jasmonic acid (JA) signal transduction, one is a member of the basic helix–loop–helix transcription factor family, one is a member of the protein kinase superfamily, and the other three are uncharacterized proteins. Considering that no putative conserved domains have been detected for OsSYL2, we speculated that the *OsSYL2* gene may regulate SYL through participation in the JA signal transduction pathway. Yang *et al*. (2012) reported that there was an antagonistic effect between JA and gibberellic acid (GA). Gibberellic acid causes elongation of the cells, lengthening SYL, as reported by Zhang (2020). Therefore, we speculated that the OsSYL2^CC^ protein (containing histidine) interacted with the JAZ protein to inhibit GA, resulting in a shorter SYL. The OsSYL2^AA^ protein, containing asparagine, interacted with JAZ protein weakly or not at all, allowing us to deduce that the inhibition of GA was weakened or relieved and SYL was lengthened. These results provide the basis for the further functional research into the *OsSYL2* gene.

In this study we found that neither the *OsSYL2^AA^* allele nor the *OsSYL3^AA^* allele, which increase SYL, are found in wild rice. This suggests that the genetic factors driving TSSL length and increased outcrossing rate in wild species are different from those mapped here. We speculated that the *OsSYL2^AA^* allele or the *OsSYL3^AA^* allele were naturally occurring mutations and were selected for during rice domestication.

We also found that SYL from *indica* rice (at low latitude) was longer than that of temperate *japonica* rice (at high latitude). Collectively, we inferred that there may be selective hitchhiking between SYL and rough rice grain length because the latter became shorter from *indica* to temperate *japonica* rice, which is a consequence of artificial selection. Thus, the accessions with the two alleles, *OsSYL2^AA^* and *OsSYL3^AA^*, can be used to increase SYL in the maintainer lines (pollen parents used for multiplying the cytoplasmic male sterile lines) of hybrid *japonica* rice by crossing and marker‐assisted selection breeding methods.

## EXPERIMENTAL PROCEDURES

### Accession sampling, field planting, and phenotypic identification

#### Accession sampling

To reflect geographical distribution (different latitudes) and phenotypic differences in stigma characteristics, we selected the 353 rice accessions, including 134 landraces from China, 206 modern improved cultivars, and 13 *javanica* accessions. The 206 modern improved cultivars were from China (170), Vietnam (21), Japan (7), the Philippines (6), Indonesia (1), and Malaysia (1). The 13 *javanica* accessions were from Indonesia (9) and China (4). Information regarding the accessions, including the variety name, country of origin, latitude, and longitude, is listed in Table S1.

#### Field planting

The seeds of 353 germplasms were collected, stored, and supplied by the State Key Laboratory of Crop Genetics and Germplasm Enhancement at Nanjing Agricultural University. All 353 accessions were grown during the normal season (May to October) across six different environments, over 3 years (2014–2016) and two locations. The two locations were Nanjing (32°07′ N, 118°64′ E) in Jiangsu province and Yuanyang (35°05′ N, 113°96′ E) in Henan province. In each environment, the field trials were conducted with two replicates, using a completely randomized block design. Each plot contained 40 plants with five rows. The plants were spaced at 20 cm × 17 cm and managed in accordance with routine agricultural management practices.

#### Phenotypic evaluation

In the rice growing season we investigated three rice stigma characteristics, that is, STL, SYL, and TSSL. At the full‐bloom stage, 10 flowering spikelets were collected from the highest panicle on each individual plant and placed in an Eppendorf tube containing tap water. We used the tap water to keep the spikelets fresh and retain the original appearance of the pistil. Next, on the same day, the pistils were taken out from the glume and photographed under a stereomicroscope (10×, MC50, Guangzhou Micro‐shot Technology Co., Ltd, http://mshot.qianyan.biz/). The STL, SYL, and TSSL were measured with a micrometer (Figure 1a). For each replication, the average of 10 STLs (two stigmas per pistil) was used as the mean value for each plant. The average of 10 SYLs was used as the mean value for each plant and the average of 10 TSSLs was used as the mean value for each plant. Five plants were evaluated in each accession in each replicate.

#### Library construction and sequencing

For each of the 191 accessions to be sequenced, two blades from a single plant were collected at tillering stage (1 month after seedling transplanting) for extraction of genomic DNA using the standard cetyltrimethylammonium bromide protocol (Murray and Thompson, 1980). In accordance with the manufacturer’s instructions (Illumina, https://www.illumina.com/), 5 μg of genomic DNA from each accession was used to construct paired‐end sequencing libraries, with insert sizes of approximately 350 bp. Paired‐end 150 bp reads were obtained using the Illumina HiSeq X10 platform, and the raw sequences were further processed by removing the adaptor contamination and low‐quality reads, yielding a total of 0.532 Tb of genomic sequence data, with an average of 5.48‐fold genomic coverage for each of the 191 accessions. All library construction, sequencing, and sequence cleaning was performed by Mega Genomics‐Beijing (http://www.megagenomics.cn/mobile.php/).

For the 162 sequenced accessions, the raw Illumina sequencing data generated by Chen *et al*. (2014) (151) and Huang *et al*. (2012) (11) were downloaded from the NCBI Sequence Read Archive using the accession number PRJNA171289 and from EBI European Nucleotide Archive using the accession number ERP000106, respectively. This information is listed in Table S1.

### The SNP calling and annotation

#### The SNP calling

All paired‐end sequence reads were aligned against the Nipponbare genome sequence downloaded from the International Rice Genome Sequencing Project (IRGSP‐1.0, http://rapdb.dna.affrc.go.jp/) using Bowtie 2 software. The parameter used for read mapping was bowtie2‐x<bt2‐ids> {−1<m1>−2<m2>|‐U<r>}‐S [<hit>]. The reads used for further SNP calling must have a unique mapping position in the Nipponbare genome and a mapping score of more than 60. Finally, 95% of the total reads were mapped to the scaffolds of the Nipponbare genome; the 3% of reads which did not map to any location or mapped to multiple locations were removed. The mapping results were converted to the VCF format by the software SAMtools (version 0.1.18) (Li *et al*., 2009). The SNP calling was performed using the HaplotypeCaller of GATK 3.8‐0 (https://software.broadinstitute.org/gatk/). Those SNPs with a MAF lower than 5% in the population were removed.

#### Annotation

The software snpEff (Cingolani *et al*., 2012) was used for SNP annotation of the Nipponbare genome sequence. Exonic regions, splicing sites (within 2 bp of a splicing junction), 5′‐ and 3′‐UTRs, intronic regions, upstream and downstream regions (within a 5‐kb region upstream or downstream from the transcription start site), and intergenic regions were categorized. The SNPs in the coding exons were of two types, synonymous and non‐synonymous; the former does not cause amino acid changes whereas the latter does. The cases in which base substitutions cause a stop gain and stop loss are non‐synonymous SNPs. Indels in the exonic regions were classified according to whether there were frameshift (3 bp insertion or deletion) mutations.

### Population genetic analysis

Based on the SNP matrix of the 353 rice accessions, we calculated the simple matching coefficient for all SNPs as the genetic distance using the software SSAHA (Ning *et al*., 2001). The software PHYLIP 3.52 (Felsenstein, 1993) was used to construct the neighbor‐joining tree, and MEGA 5.0 software (Tamura *et al*., 2011) was used to display the tree. We performed principal component analysis using the smartpca program of the gcta64 software (Price *et al*., 2006), and the first two principal components were plotted in two dimensions. The population structure was analyzed using STRUCTURE 2.3.4 software (Pritchard *et al*., 2000). We set the parameter for admixture and no linkage to run STRUCTURE 2.3.4 software with a burn‐in of 50 000 replicates and 100 000 Markov chain Monte Carlo iterations. For each *K* value (*K* = 1–10), 10 repeats were performed. We analyzed the results by the EVANNO method with STRUCTURE HARVESTER (Earl and vonHoldt, 2012) and used CLUMPP (version 1.1.2) (Jakobsson and Rosenberg, 2007) to permute run clusters. If the mean log‐likelihood value increased with an increase in *K* value, Δ*K* values were calculated to determine a suitable *K* value. DISTRUCT (Rosenberg, 2004) was used to plot the results. We determined the non‐admixed individuals in each genetic subpopulation with *Q*‐matrix assignment of above 0.85.

The LD was estimated using the *r* ^2^ value (Mather *et al*., 2007) for all inter‐ and intra‐chromosomal SNP pairs and was calculated using PopLDdecay 3.40 software (Zhang *et al*., 2018), with default parameters. The LD decay was calculated by grouping the SNP pairs into 1‐kb bins, and averaging the *r* ^2^ within bins; the average *r* ^2^ values were then used to plot the fitting curve line for the whole population, and the *indica* and *japonica* subpopulations. The LD heatmaps surrounding peaks in the GWAS were constructed using the R package ‘LDheatmap’ (Shin *et al*., 2006).

Across the rice genome, the nucleotide diversity (π) of *indica* rice and *japonica* rice in each 100 kb was calculated by the program ANGSD (version 0.613) (Korneliussen *et al*., 2014). We calculated the *F*_ST_ between the *indica* subpopulation and the *japonica* subpopulation by VCFtools (version 0.1.12b) (Danecek *et al*., 2011) based on the Weir and Cockerham method (Weir and Cockerham, 1984).

### Genome‐wide association mapping

We carried out a GWAS on a total of 18 sets of phenotypic data (six environments × three traits) and the genotypic data of 1 326 094 common SNPs (MAF > 0.05) with a mixed linear model (MLM) program by GAPIT (version 2.12) (Lipka *et al*., 2012). The false discovery rate (FDR) was calculated for significant associations using the Benjamini and Hochberg (1995) correction method, with 1.0 × 10^−5^ as the threshold. The Manhattan and quantile–quantile plots were generated by the qqman (Turner, 2014) package in R. The candidate genes in these associated loci were identified by BLAST query of the Nipponbare genome to obtain coordinates and were confirmed as significantly associated with the corresponding traits.

### Identification of the candidate genes in the GWAS‐associated loci

We took the following steps to narrow the candidate gene region. First, based on the associated loci identified, the candidate region was estimated by pairwise LD correlation (Shin *et al*., 2006). Second, according to the reference genome sequence of Nipponbare, the SNP types located in the candidate region were analyzed. We focused on the associated non‐synonymous SNPs which could induce amino acid changes and were significantly associated with the traits in the GWAS result. Third, the different expression of candidate genes between three samples with shorter SYL and three samples with longer SYL was used to narrow the candidate genes. We then conducted the gene‐based association analysis to classify the samples into distinct alleles. Finally, to confirm the causal SNPs per gene, the difference between phenotypes with distinct alleles was calculated and the significance was tested by a two‐tailed Welch’s *t*‐test.

### The RNA extraction procedure and quantitative real‐time PCR (qRT‐PCR)

Using an ultrapure RNA kit (Omega Bio‐tek, https://www.omegabiotek.com), total RNA was extracted from young panicles at development stages 5–8 (as per the criterion described by Itoh *et al*., 2005) and young pistils at stage 8, respectively, sampled from the six accessions (three accessions with shorter SYL and three accessions with longer SYL). We used RNase‐free DNase I treatment to remove any contamination from genomic DNA and HiScript II Q RT SuperMix (Vazyme, http://www.vazyme.com) to perform the first‐strand cDNA synthesis by reverse transcription from 1 µg of RNA. The 18S rRNA gene was used as an internal control. Real‐time quantitative RT‐PCR was conducted in a 96‐well thermocycler (Roche Applied Science LightCycler 480, https://lifescience.roche.com/) using SYBR Green (Vazyme). We set the following cycling conditions: first, denaturation, at 95°C for 5 min; followed by an amplification and quantification program, 40 cycles of 95°C for 10 sec, 60°C for 30 sec, and 72°C for 60 sec with a single fluorescence measurement; and third, the melting curve (60–95°C with a heating rate of 0.1°C sec^–1^ and continuous fluorescence measurement); and finally, a cooling step to 40°C. Three independent replicates were performed. The sequences of the primers used for qRT‐PCR are listed in Table S11. Relative gene expression of the target gene was calculated following the equation: exp = 2^−ΔCt^, where ΔCt = Ct_target gene_−Ct_18S rRNA_.

### Generation of *Os02g0733900* transgenic plants

The full‐length genomic DNA of *Os02g0733900* was PCR amplified from Xiangxiandao 10hao. The PCR product was cloned into the pBWA(V)HII vector. The primer sets used for PCR are listed in Table S11. The construct was introduced into *Agrobacterium tumefaciens* (EHA105) and transferred into Nipponbare. The corresponding empty vector was also transformed into Nipponbare as a control. Twenty‐five independent T_1_ seedlings obtained were grown in a paddy field under natural conditions. The T_2_ seeds harvested from T_1_ plants at the maturity stage were grown in the paddy field in the next rice growing season (May to October). At the tillering stage, the three allele genotypes (AA, AC, CC) on the *Os02g0733900* locus were determined using the primers listed in Table S11 and the stigma characteristics were measured in the *Os02g0733900^AA^* and *Os02g0733900^CC^* plants at flowering stage.

### Evaluation of F_1_ hybrid seed production potential for *OsSYL2* in the paddy field

To evaluate the potential of the *OsSYL2* gene in F_1_ hybrid rice seed production we selected Nipponbare (short stigma), transgenic complementary line with *OsSYL2^AA^* (long stigma), as the female parent, and a purple rice accession as the male parent. The female and male lines were grown in a 4:2 row ratio. The male line was planted on both sides in four rows each, and the female material was planted in the middle in two rows. During the flowering stage, artificial supplementary pollination was performed twice per day. After 30 days, the seeds of the female lines were harvested. The potential of OsSYL2 for hybrid rice seed production was evaluated by detecting the percentage of purple seedlings in the germination experiment in the F_1_ populations. Ten thousand ‘F_1_’ seeds were sampled from each F_1_ combination and sown in plastic plates (39 cm × 39 cm × 6 cm) to investigate the frequency of purple seedlings. Two replicates were conducted for each combination.

## Author contributions

DH and XD designed the experiments and managed the project. XD, YZ, XC, ZF, QL, JJ, DL, YL, BF, ZW, EL, XH, SZ, DS, HW, YL, and SC conducted field planting, phenotyping, and DNA sampling. XD and YZ prepared DNA samples, qRT‐PCR and transformation analysis, and performed the test evaluating the potential of the *OsSYL2* gene in hybrid rice seed production. XD and YY performed the data analysis. XD and YY wrote the manuscript draft, which was revised by YW and DH.

## Conflict of interest

The authors declare no competing financial interests.

## Supporting information


**Figure S1.** Phenotypic distribution of stigma length, style length and the sum of stigma and style length traits in the germplasm collection in six environments.
**Figure S2.** Genetic diversity across 12 chromosomes.
**Figure S3.** Population structure analysis of 353 rice accessions and the decay of linkage disequilibrium.
**Figure S4.** Neighbor‐joining tree with accession ID.
**Figure S5.** Manhattan plots and quantile–quantile plots depicting the results of genome‐wide association study for the stigma length trait using a mixed line model in the 353 cultivated rice accessions in each environment.
**Figure S6.** Manhattan plots and quantile–quantile plots depicting the results of genome‐wide association study for the style length trait using a mixed line model in the 353 cultivated rice accessions in each environment.
**Figure S7.** Manhattan plots and quantile–quantile plots depicting the results of genome‐wide association study for the sum of stigma and style length trait using a mixed line model in the 353 cultivated rice accessions in each environment.
**Figure S8.** The morphology of young panicles in different development stages.
**Figure S9.** The gene allele frequency differences at the causal polymorphisms of *OsSYL2* and *OsSYL3* in five geographic groups.
**Figure S10.** Protein networks interacting with *OsSYL2*. Network nodes represent proteins and edges represent protein–protein association.

**Table S1.** Names and origins of 353 rice accessions used for association mapping and the corresponding *Q*‐values calculated by STRUCTURE software.

**Table S2.** Basic statistics of the three stigma characteristics in each environment.

**Table S3.** The results of joint analysis of variance for the three stigma characteristics.

**Table S4.** The distribution of the significant association single‐nucleotide polymorphism loci detected in the rice population composed of 353 accessions in the six environments.

**Table S5.** The single‐nucleotide polymorphism information in the 16.69–16.87 Mb candidate region for style length and the sum of stigma and style length traits.

**Table S6.** Candidate gene annotation in the linkage disequilibrium region 16.69–16.87 Mb associated with style length and the sum of stigma and style length traits.

**Table S7.** The single‐nucleotide polymorphism information in the 30.45–30.65 Mb candidate region for style length trait.

**Table S8.** Candidate gene annotation in the linkage disequilibrium region 30.45–30.65 Mb associated with style length trait.

**Table S9.** Correlation coefficients between grain length trait and stigma characteristics.

**Table S10.** The results of significantly associated single‐nucleotide polymorphism loci detected in this study overlapped with the quantitative trait loci/genes of rice stigma characteristics reported previously.

**Table S11.** Primers used in this study.

## Data Availability

Sequencing data that support the findings of this study have been deposited in the Sequence Read Archive (SRA), NCBI with the accession code PRJNA554986.
