# Supplementary material for: OsSYL2 AA, an allele identified by gene‐based association, increases style length in rice (Oryza sativa L.)
Source: Plant J. 2020 Oct 30;104(6):1491–503. doi: 10.1111/tpj.15013 (PMC7821000; doi:10.1111/tpj.15013)
Supplement: Supplementary file 1 — Figure S1. Phenotypic distribution of stigma length, style length and the sum of stigma and style length traits in the germplasm collection in six environments. Figure S2. Genetic diversity across 12 chromosomes. Figure S3. Population structure analysis of 353 rice accessions and the decay of linkage disequilibrium. Figure S4. Neighbor‐joining tree with accession ID. Figure S5. Manhattan plots and quantile–quantile plots depicting the results of genome‐wide association study for the stigma length trait using a mixed line model in the 353 cultivated rice accessions in each environment. Figure S6. Manhattan plots and quantile–quantile plots depicting the results of genome‐wide association study for the style length trait using a mixed line model in the 353 cultivated rice accessions in each environment. Figure S7. Manhattan plots and quantile–quantile plots depicting the results of genome‐wide association study for the sum of stigma and style length trait using a mixed line model in the 353 cultivated rice accessions in each environment. Figure S8. The morphology of young panicles in different development stages. Figure S9. The gene allele frequency differences at the causal polymorphisms of OsSYL2 and OsSYL3 in five geographic groups. Figure S10. Protein networks interacting with OsSYL2. Network nodes represent proteins and edges represent protein–protein association. [file TPJ-104-1491-s001.pdf]

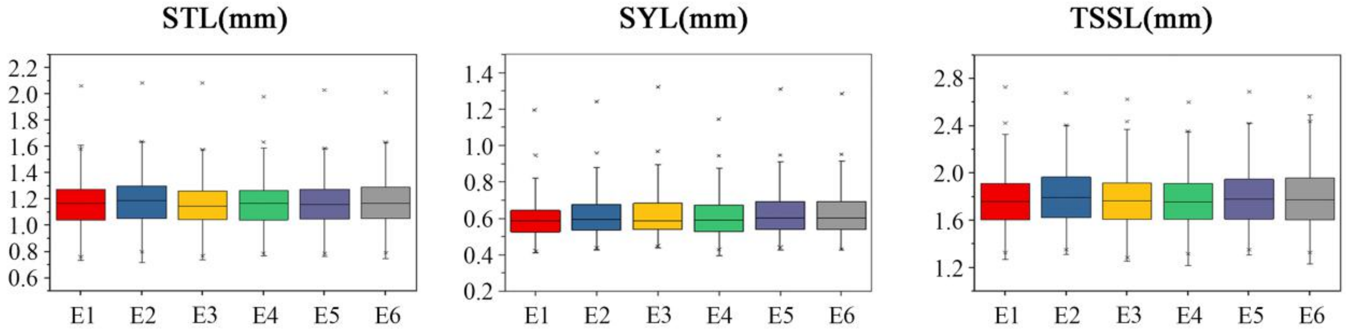

Figure S1. Phenotypic distribution of STL, SYL and TSSL traits in the germplasm collection in six environments. STL, stigma length; SYL, style length. TSSL, the sum of stigma and style length. E1, Nanjing 2014; E2, Nanjing 2015; E3, Nanjing 2016; E4, Yuanyang 2014; E5, Yuanyang 2015; E6, Yuanyang 2016.

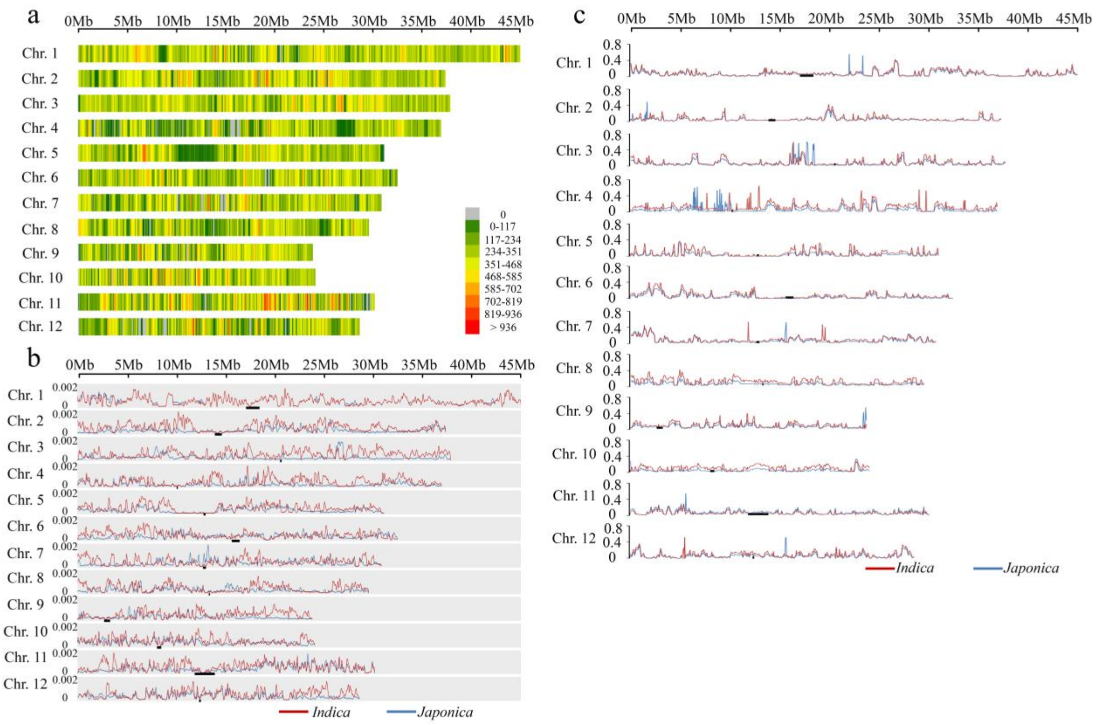

Figure S2. Genetic diversity across 12 chromosomes. (a) SNP density. The number of SNPs within a 100-kb window is indicated by the color index. (b) Sequence diversity (π) along chromosomes in *Indica* and *Japonica*, respectively. Black blocks on chromosomes indicate the centromeres of the reference genome. The red lines indicate *Indica* and the blue lines indicate *Japonica*. (c) Population genetic differentiation (F_{ST}). F_{ST} among the subpopulations of *indica* and *japonica* was calculated in 100-kb windows. Black blocks on chromosomes indicated the centromeres of the reference genome. The red lines indicate *Indica* and the blue lines indicate *Japonica*.

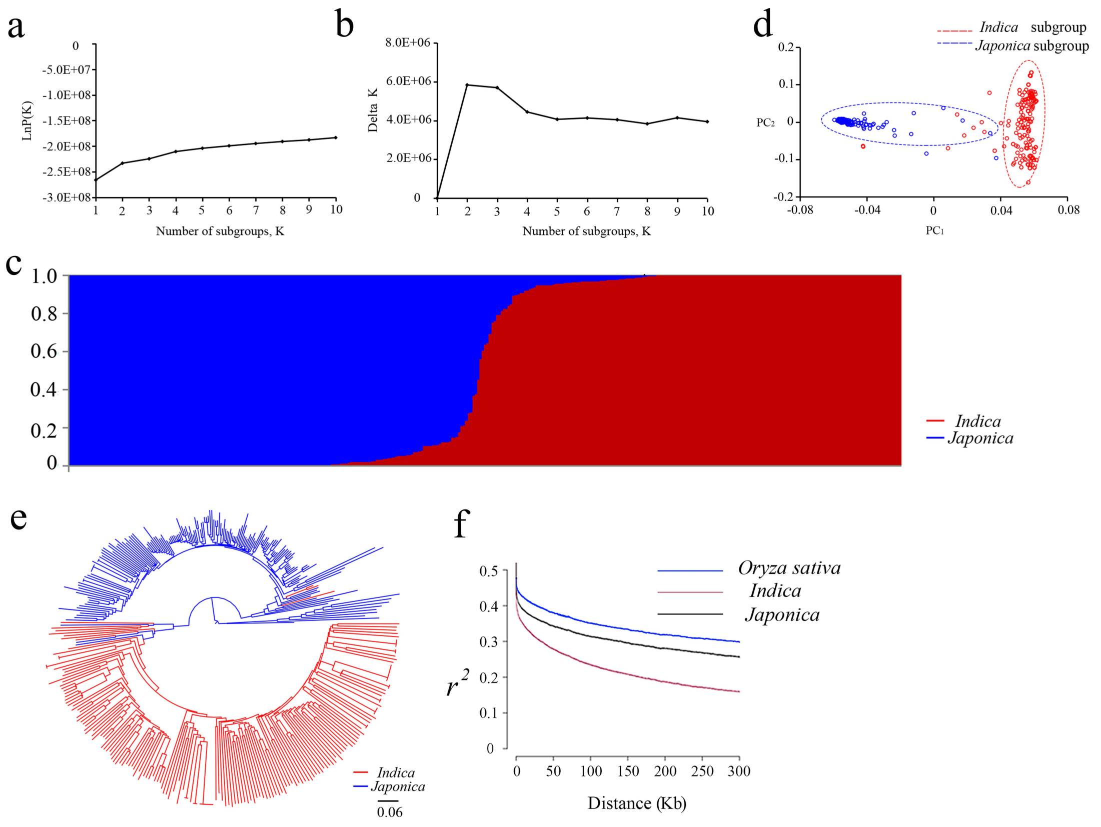

Figure S3. Population structure analysis of 353 rice accessions and the decay of linkage disequilibrium. (a) Changes of the mean LnP(K). Graph of mean LnP(K) on the y axis and the number of subgroups on the x axis. (b) Changes in the mean delta K. Graph of mean delta K on y axis and number of subgroups on x axis. (c) Population structure based on a Bayesian model. Each vertical bar represents one accession, and the length of each vertical bar represents the ancestral proportion. (d) Principal component analysis showing the population structure in the diversity panel. The red lines indicate *Indica* accessions. The blue lines indicate *Japonica* accessions. (e) Phylogenetic tree of 353 accessions based on whole-genome SNPs. The scale bar at the bottom represents genetic distance. Accessions within different subgroups are displayed by different colors. (f) Linkage disequilibrium differences among the full cultivated rice population, *indica* subpopulation and *japonica* subpopulation.

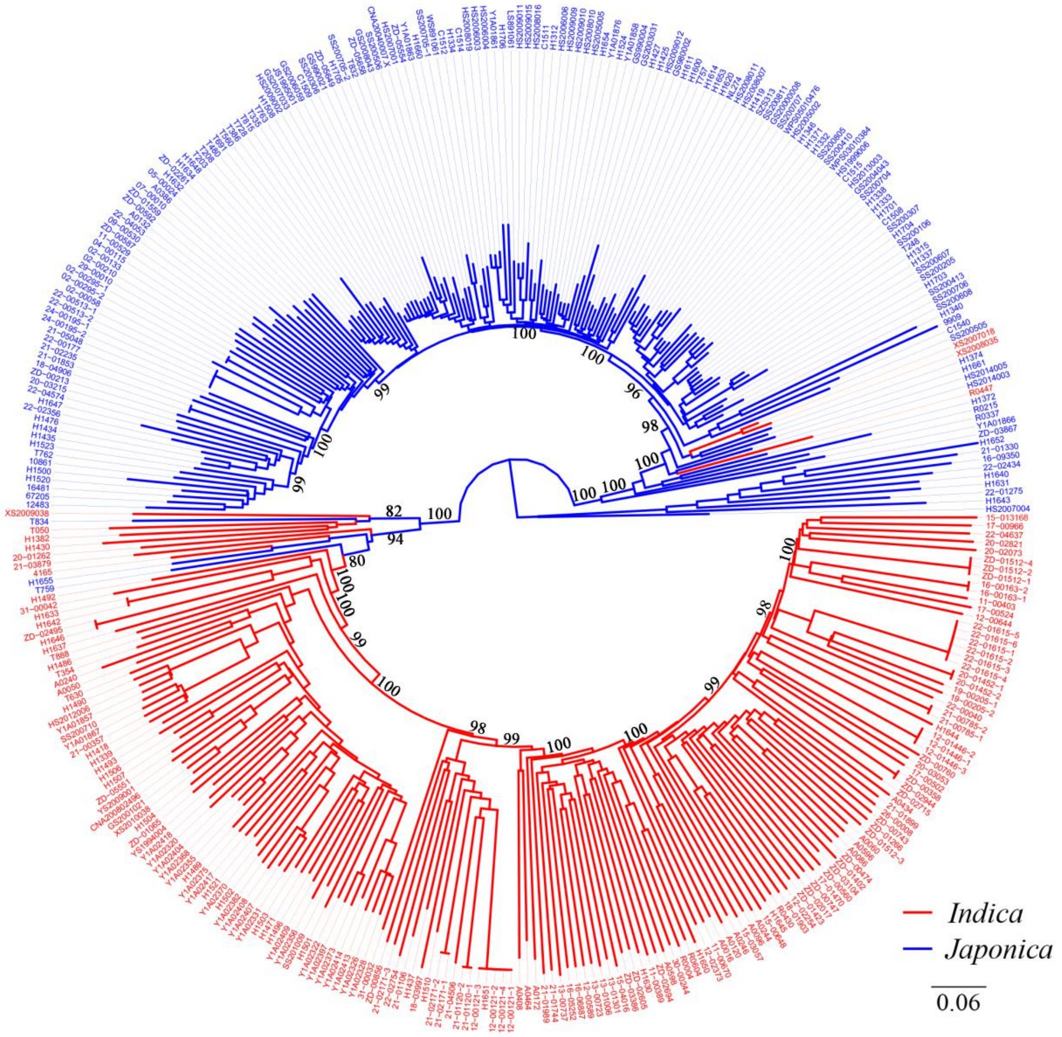

Figure S4. Neighbor-joining tree with accession ID. Neighbor-joining tree reconstructed from whole-genome SNPs of 353 cultivated rice accessions by software PHYLIP 3.52 (Felsenstein, 1993) and displayed by MEGA 5.0 (Tamura *et al*., 2011). Each branch is corresponding to each accession ID. Each accession ID corresponding to the rice accession name is presented in Table S1. The scale bar at the bottom represents genetic distance. Numbers at nodes indicate percentage of 1000 bootstrap replicates.

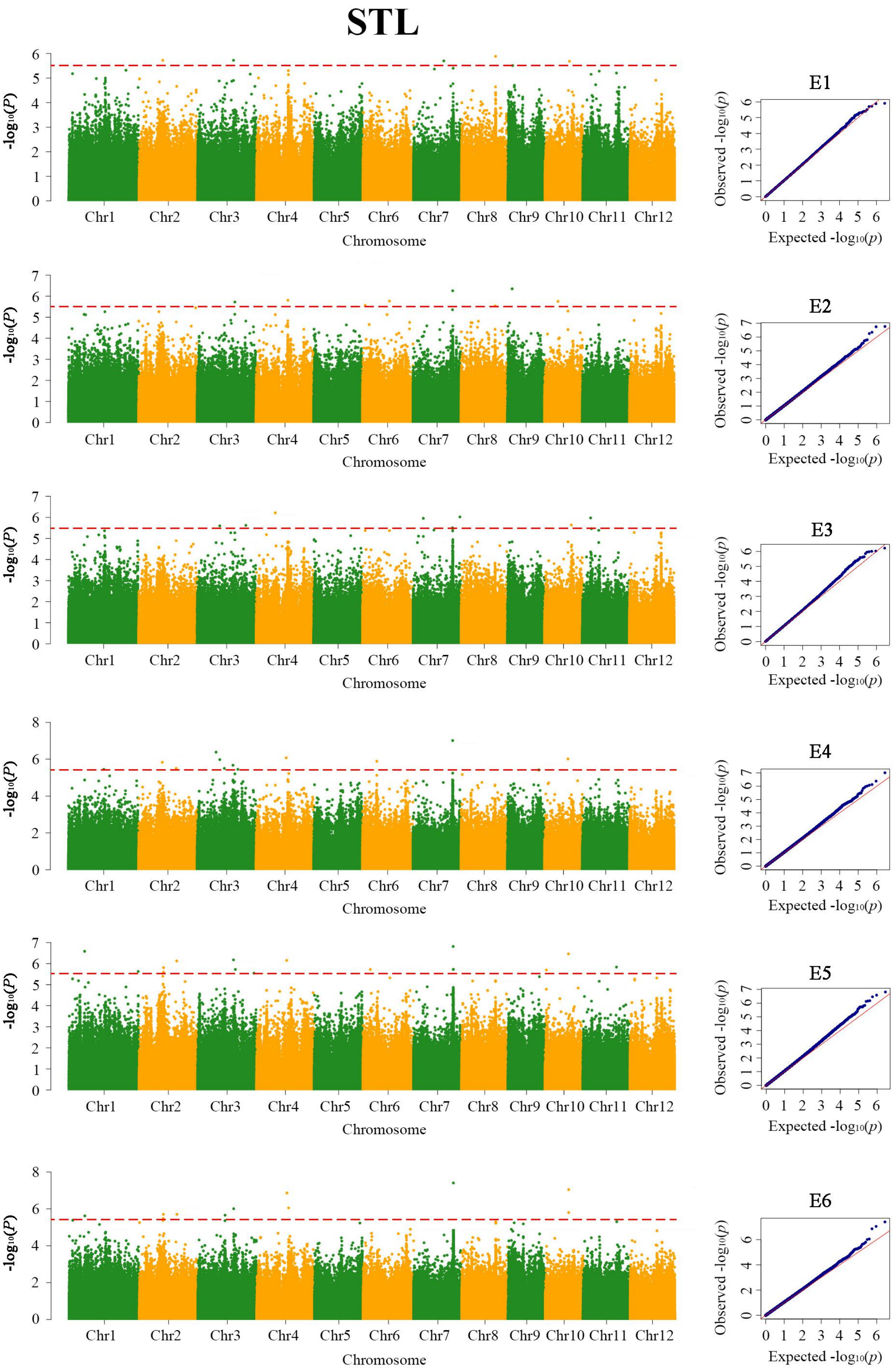

Figure S5. Manhattan plots and quantile-quantile plots depicting GWAS results for the STL trait using a mixed line model in the 353 cultivated rice accessions in each environment.

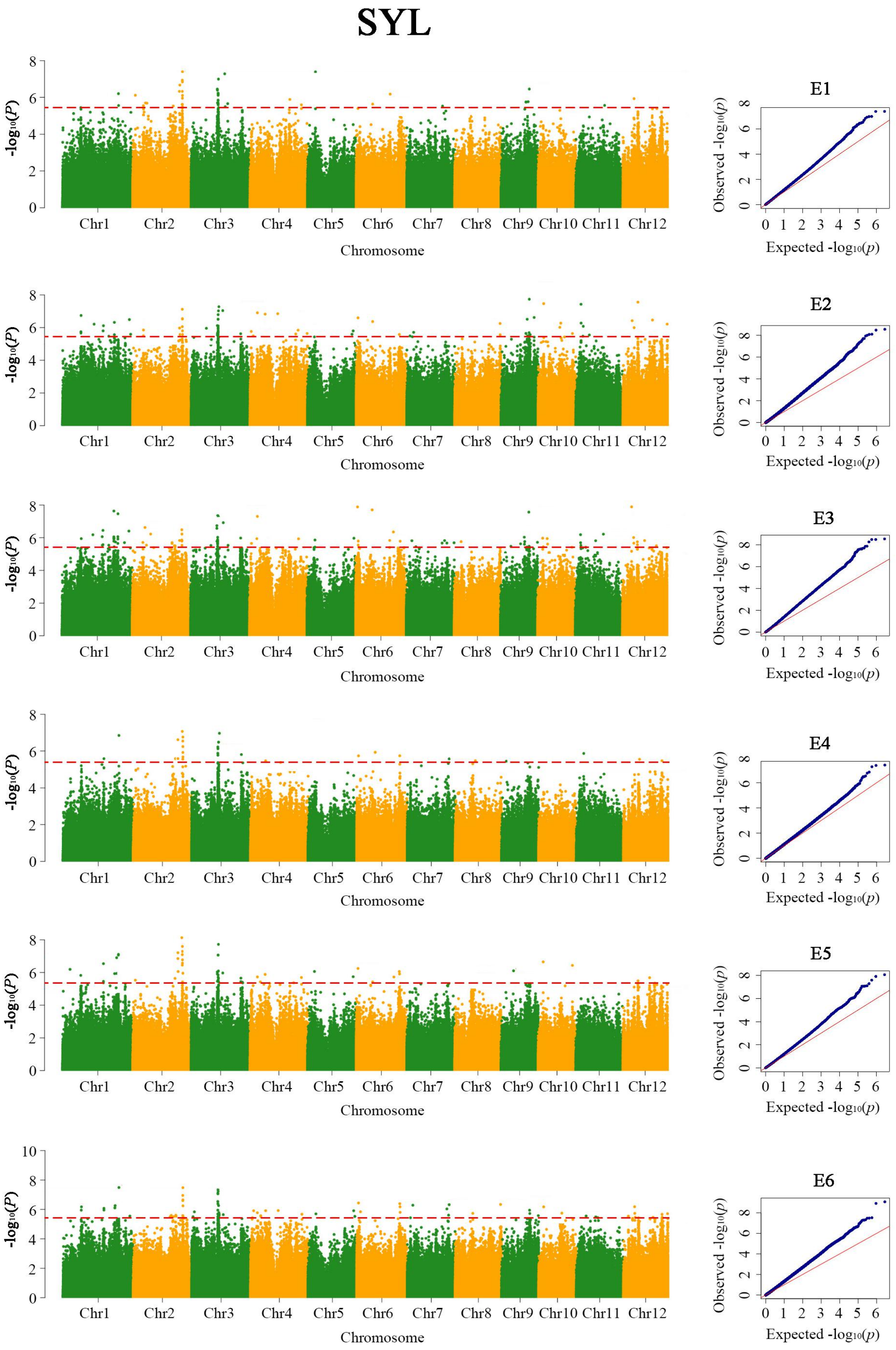

Figure S6. Manhattan plots and quantile-quantile plots depicting GWAS results for the SYL trait using a mixed line model in the 353 cultivated rice accessions in each environment.

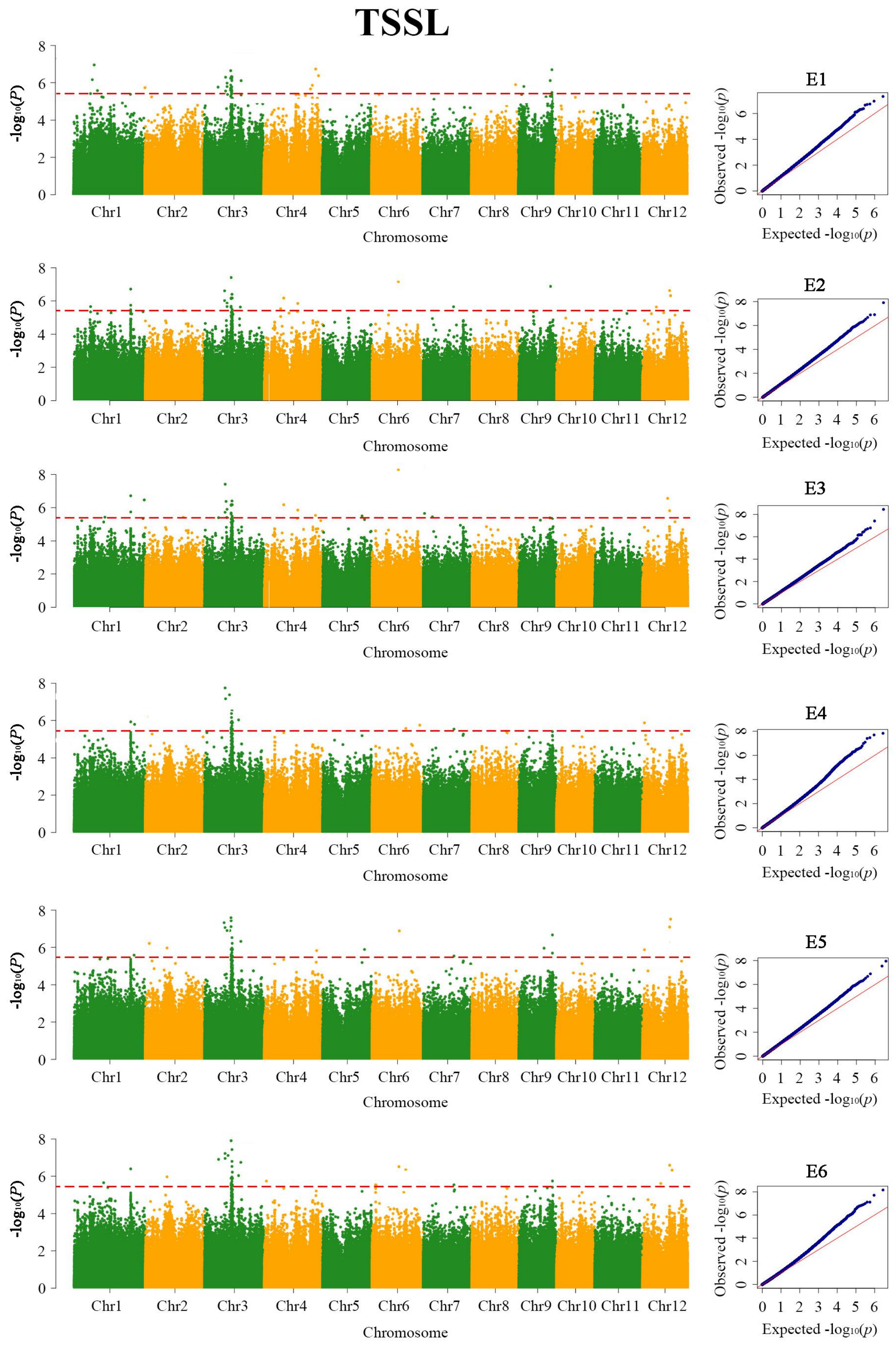

Figure S7. Manhattan plots and quantile-quantile plots depicting GWAS results for the TSSL trait using a mixed line model in the 353 cultivated rice accessions in each environment.

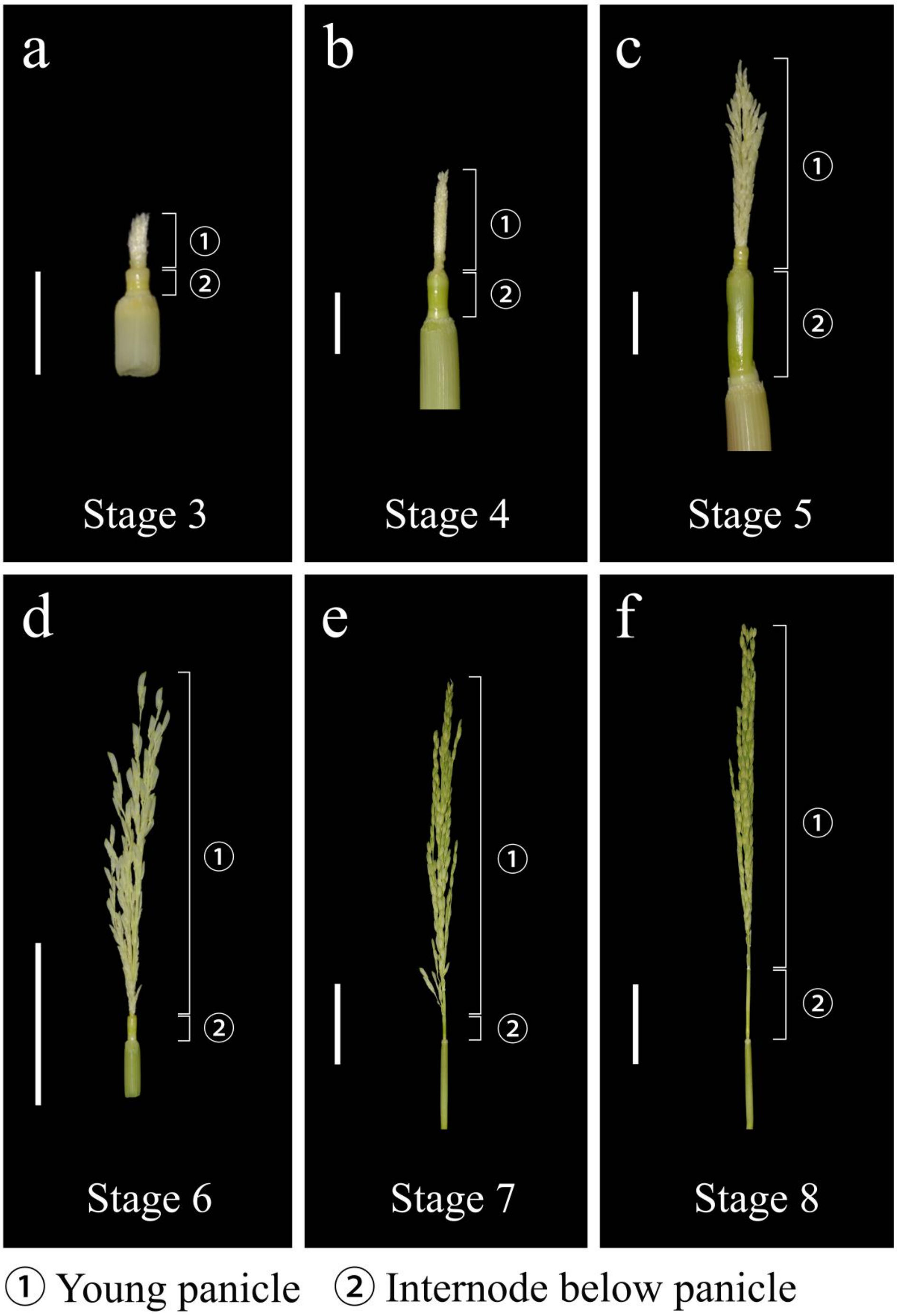

Figure S8. The morphology of rice young panicle at different development stages. (a) Rice young panicle development Stage 3. Scale bar=1cm. (b) Rice young panicle development Stage 4. Scale bar=1cm. (c) Rice young panicle development Stage 5. Scale bar=1cm. (d) Rice young panicle development Stage 6. Scale bar=5cm. (e) Rice young panicle development Stage 7. Scale bar=5cm. (f) Rice young panicle development Stage 8. Scale bar=5cm. The brackets represented young panicle and internode below panicle, respectively.

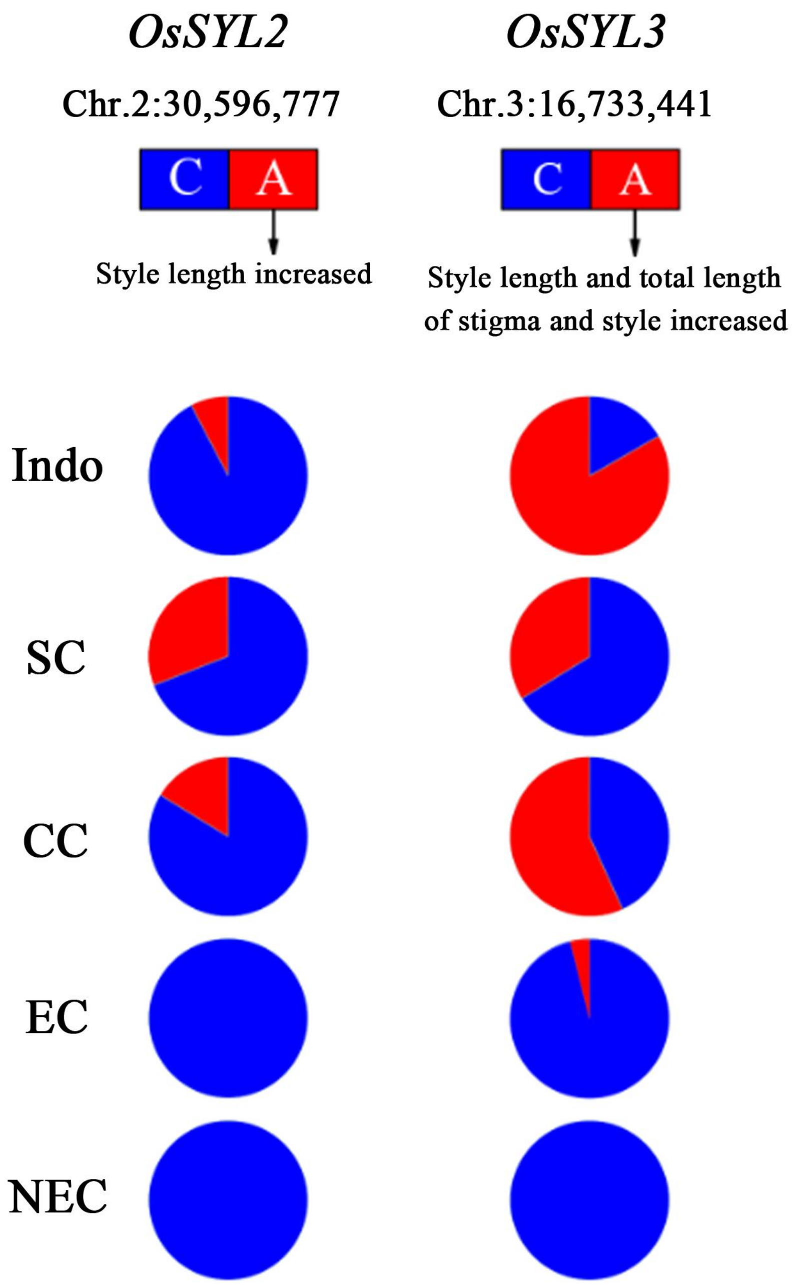

Figure S9. The gene allele frequency differences at the causal polymorphisms of *OsSYL2* and *OsSYL3* in five geographic groups. The type of reference allele is indicated in blue, and the alternative is indicated in red. Indo, Indonesia; SC, southern China; CC, central China; EC, eastern China; NEC, northeastern China. The accessions from Indo were mainly tropical *Japonica* subspecies. The accessions from SC and CC were mainly *Indica* subspecies. The accessions from EC and NEC were mainly temperate *Japonica* subspecies

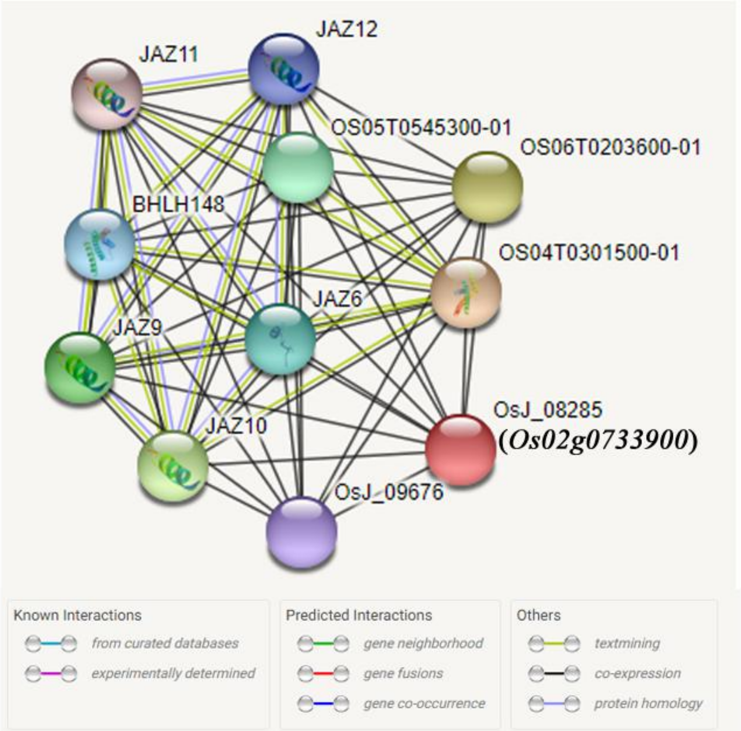

Your Input:

OsJ_08285	*cDNA clone-002-181-F02, full insert sequence; Os02g0733900 protein; Putative uncharacterized protein P0643A10.41; cDNA clone-002-181-F02, full insert sequence (80 aa)*

Predicted Functional Partners:

		Neighborhood	Gene Fusion	Cooccurence	Coexpression	Experiments	Databases	Textmining	[Homology]	Score
OS04T0301500-01	*Os04g0301500 protein; cDNA, clone- J075170K16, full insert sequence (310 aa)*				●					0.634
OS06T0203600-01	*cDNA clone-J023143N19, full insert sequence; Os06g0203600 protein; Putative uncharacterized protein OSJ...*				●					0.630
JAZ10	*Protein TIFY 11b; Repressor of jasmonate responses (187 aa)*				●					0.600
JAZ9	*Protein TIFY 11a; Repressor of jasmonate (JA) responses. Forms a ternary complex with RSS3 and BHLH94 t...*				●					0.585
OS05T0545300-01	*Os05g0545300 protein ; Belongs to the protein kinase superfamily (441 aa)*				●					0.572
JAZ6	*Protein TIFY 10a; Repressor of jasmonate responses (228 aa)*				●					0.558
BHLH148	*Transcription factor BHLH148; May act on an initial response of jasmonate-regulated gene expression toward...*				●					0.518
JAZ12	*Protein TIFY 11d; Repressor of jasmonate (JA) responses (By similarity). May act on an initial response of JA-...*				●					0.487
OsJ_09676	*cDNA clone-001-110-D07, full insert sequence; Expressed protein; Os03g0183500 protein; Putative uncharact...*				●					0.474
JAZ11	*Protein TIFY 11c; Repressor of jasmonate responses (209 aa)*				●					0.471

Figure S10. Protein networks interacting with *OsSYL2*. Network nodes represent proteins. Edges represent protein-protein association.
