# Supplementary material for: OsSYL2 AA, an allele identified by gene‐based association, increases style length in rice (Oryza sativa L.)
Source: Plant J. 2020 Oct 30;104(6):1491–503. doi: 10.1111/tpj.15013 (PMC7821000; doi:10.1111/tpj.15013)
Supplement: Supplementary file 2 — Table S1. Names and origins of 353 rice accessions used for association mapping and the corresponding Q‐values calculated by STRUCTURE software. [file TPJ-104-1491-s002.docx]

**Table S1.** Names and origins of 353 rice accessions used for association mapping and the corresponding Q values calculated by STRUCTURE software.

| Code | Germplasm name | Origin | Latitude | Longitude | Depth | Sourcea | Germplasm IDb | Q value | | Subpopulation |
| --- | --- | --- | --- | --- | --- | --- | --- | --- | --- | --- |
|  |  |  |  |  |  |  |  | Q1 | Q2 |  |
| 1 | Longjing 20 | Heilongjiang, China | 44.04 | 125.42 | 6.8 | this study | HS2007004 | 0.00001 | 0.99999 | Japonica |
| 2 | Huangdao | Guizhou, China | 26.35 | 106.42 | 1.48 | ERS006477 | 22-01275 | 0.00001 | 0.99999 | Japonica |
| 3 | Wuqiang | Jiangsu,China | 34.54 | 118.75 | 1.21 | ERS039280 | H1631 | 0.00001 | 0.99999 | Japonica |
| 4 | Yangdao | Jiangsu,China | 31.32 | 120.62 | 1.36 | ERS039332 | H1640 | 0.00001 | 0.99999 | Japonica |
| 5 | Zigu | Guizhou, China | 26.87 | 104.28 | 1.14 | ERS039314 | 22-02434 | 0.00001 | 0.99999 | Japonica |
| 6 | Hongnuogu | Guizhou,China | 26.49 | 113.77 | 0.66 | ERS039345 | H1652 | 0.00001 | 0.99999 | Japonica |
| 7 | Heizhong | Shanxi,China | 32.82 | 106.25 | 1.01 | ERS039288 | 21-01330 | 0.00001 | 0.99999 | Japonica |
| 8 | Yangfujing 8hao | Jiangsu,China | 34.26 | 117.20 | 5.7 | this study | SS200608 | 0.00001 | 0.99999 | Japonica |
| 9 | Yangfujing 7hao | Jiangsu,China | 34.26 | 117.20 | 5.5 | this study | SS200413 | 0.00001 | 0.99999 | Japonica |
| 10 | Sihao 4040 | Jiangsu,China | 34.26 | 117.20 | 5.8 | this study | H1703 | 0.00001 | 0.99999 | Japonica |
| 11 | Nannongjing 004 | Jiangsu,China | 32.04 | 118.78 | 5.8 | this study | T248 | 0.00001 | 0.99999 | Japonica |
| 12 | Zhendao 99 | Jiangsu,China | 34.26 | 117.20 | 5.8 | this study | SS200106 | 0.00001 | 0.99999 | Japonica |
| 13 | Sihao 4330 | Jiangsu,China | 34.26 | 117.20 | 5.9 | this study | H1704 | 0.00001 | 0.99999 | Japonica |
| 14 | Huifeng 1 | Jiangsu,China | 33.38 | 120.13 | 5.9 | this study | C1508 | 0.00001 | 0.99999 | Japonica |
| 15 | Yandao 8hao | Jiangsu,China | 33.38 | 120.13 | 5.9 | this study | SS200307 | 0.00001 | 0.99999 | Japonica |
| 16 | Sihao 4385 | Jiangsu,China | 33.46 | 118.23 | 5.3 | this study | H1315 | 0.00001 | 0.99999 | Japonica |
| 17 | Shengdao 14 | Jiangsu,China | 34.26 | 117.20 | 5.8 | this study | H1701 | 0.00001 | 0.99999 | Japonica |
| 18 | Wanqu 429bp | Jiangsu,China | 34.26 | 117.20 | 6 | this study | HS2013003 | 0.00001 | 0.99999 | Japonica |
| 19 | Suijing 3hao | Heilongjiang, China | 44.04 | 125.42 | 5.9 | this study | HS1999006 | 0.00001 | 0.99999 | Japonica |
| 20 | Ningjing 2hao | Jiangsu,China | 34.26 | 117.20 | 5.9 | this study | WPS05010476 | 0.00001 | 0.99999 | Japonica |
| 21 | Yanjing 9hao | Jiangsu,China | 34.26 | 117.20 | 5.6 | this study | SS200707 | 0.00001 | 0.99999 | Japonica |
| 22 | 9522B | Jiangsu,China | 31.79 | 119.95 | 6 | this study | GS20000008 | 0.00001 | 0.99999 | Japonica |
| 23 | Yangfujing 4901 | Jiangsu,China | 34.26 | 117.20 | 5.7 | this study | SS200811 | 0.00001 | 0.99999 | Japonica |
| 24 | Xudao 25-7 | Jiangsu,China | 34.26 | 117.20 | 6.1 | this study | H1419 | 0.00001 | 0.99999 | Japonica |
| 25 | Wuyunjing 8hao | Jiangsu,China | 31.78 | 119.95 | 6 | this study | SZS313 | 0.00001 | 0.99999 | Japonica |
| 26 | Ningjinghui 260 | Jiangsu,China | 32.04 | 118.78 | 5 | this study | H1371 | 0.00001 | 0.99999 | Japonica |
| 27 | Qiyunuo 10hao | Jiangsu,China | 34.26 | 117.20 | 5.6 | this study | HS2005002 | 0.00001 | 0.99999 | Japonica |
| 28 | Jingnuo 330 | Anhui,China | 31.86 | 117.27 | 5.7 | this study | H1346 | 0.00001 | 0.99999 | Japonica |
| 29 | Sihao 4141 | Jiangsu,China | 33.46 | 118.23 | 6 | this study | H1332 | 0.00001 | 0.99999 | Japonica |
| 30 | Huaidao 11hao | Jiangsu,China | 33.50 | 119.15 | 6 | this study | SS200805 | 0.00001 | 0.99999 | Japonica |
| 31 | Huaidao 8hao | Jiangsu,China | 33.50 | 119.15 | 5.8 | this study | SS200410 | 0.00001 | 0.99999 | Japonica |
| 32 | Hejing 1hao | Heilongjiang,China | 45.45 | 126.41 | 6 | this study | HS2008007 | 0.00001 | 0.99999 | Japonica |
| 33 | Zhongguo 91 | Japan | 35.68 | 139.69 | 5.9 | this study | NL274 | 0.00001 | 0.99999 | Japonica |
| 34 | Kendao 13 | Heilongjiang,China | 45.45 | 126.41 | 6 | this study | HS2008011 | 0.00001 | 0.99999 | Japonica |
| 35 | Zhongjing 131 | Jiangsu,China | 32.04 | 118.78 | 5.8 | this study | H1620 | 0.00001 | 0.99999 | Japonica |
| 36 | Zaijinjing | Jiangsu,China | 32.04 | 118.78 | 5.8 | this study | H1614 | 0.00001 | 0.99999 | Japonica |
| 37 | Wanjingnuo | Anhui,China | 31.86 | 117.27 | 5.7 | this study | H1653 | 0.00001 | 0.99999 | Japonica |
| 38 | Hongnong 5hao | Jiangsu,China | 31.16 | 120.63 | 6 | this study | T757 | 0.00001 | 0.99999 | Japonica |
| 39 | Wunuoyihao | Jiangsu,China | 44.04 | 125.42 | 5.8 | this study | H1611 | 0.00001 | 0.99999 | Japonica |
| 40 | Jianongnuo 2hao | Jiangsu,China | 32.02 | 118.5 | 5.7 | this study | H1600 | 0.00001 | 0.99999 | Japonica |
| 41 | Kendao 19 | Heilongjiang, China | 44.04 | 125.42 | 8.4 | this study | HS2009012 | 0.00001 | 0.99999 | Japonica |
| 42 | Yujing 6hao | Henan,China | 34.76 | 113.65 | 6 | this study | GS980002 | 0.00001 | 0.99999 | Japonica |
| 43 | Zhongjing 9677 | Jiangsu,China | 32.04 | 118.78 | 6 | this study | C1512 | 0.00001 | 0.99999 | Japonica |
| 44 | IL38 | Japan | 35.68 | 139.69 | 5.9 | this study | ZD-05554 | 0.00001 | 0.99999 | Japonica |
| 45 | RT61 | Japan | 35.68 | 139.69 | 6 | this study | Y1A01863 | 0.00001 | 0.99999 | Japonica |
| 46 | Yueguang | Japan | 35.68 | 139.69 | 5.9 | this study | H1660 | 0.00001 | 0.99999 | Japonica |
| 47 | Longjing 17 | Heilongjiang, China | 44.04 | 125.42 | 5.8 | this study | HS2007001 | 0.00001 | 0.99999 | Japonica |
| 48 | Xudao 4hao | Jiangsu,China | 34.26 | 117.20 | 6 | this study | CNA20040007.X | 0.00001 | 0.99999 | Japonica |
| 49 | Yandao 9hao | Jiangsu,China | 33.38 | 120.13 | 5.9 | this study | SS200506 | 0.00001 | 0.99999 | Japonica |
| 50 | Suyunuo | Jiangsu,China | 34.26 | 117.20 | 6 | this study | T832 | 0.00001 | 0.99999 | Japonica |
| 51 | Zhen9424 | Jiangsu,China | 34.26 | 117.20 | 5.9 | this study | ZD-05658 | 0.00001 | 0.99999 | Japonica |
| 52 | Yangguang 200 | Jiangsu,China | 34.26 | 117.20 | 5.8 | this study | GS2008043 | 0.00001 | 0.99999 | Japonica |
| 53 | Wuyunjing 21hao | Jiangsu,China | 34.26 | 117.20 | 6.1 | this study | SS200705-2 | 0.00001 | 0.99999 | Japonica |
| 54 | Sihao 4280 | Jiangsu,China | 34.26 | 117.20 | 5.8 | this study | H1705 | 0.00001 | 0.99999 | Japonica |
| 55 | Lianjing 2hao | Jiangsu,China | 34.26 | 117.20 | 5.8 | this study | GS990021 | 0.00001 | 0.99999 | Japonica |
| 56 | Yanjing 8hao | Jiangsu,China | 34.26 | 117.20 | 5.8 | this study | ZD-05649 | 0.00001 | 0.99999 | Japonica |
| 57 | Xudao 3hao | Jiangsu,China | 34.26 | 117.20 | 5.9 | this study | SS200306 | 0.00001 | 0.99999 | Japonica |
| 58 | Xudao 5hao | Jiangsu,China | 34.26 | 117.20 | 5.8 | this study | GS2006059 | 0.00001 | 0.99999 | Japonica |
| 59 | Zhengdao 18 | Henan,China | 34.76 | 113.65 | 7.5 | this study | GS2007033 | 0.00001 | 0.99999 | Japonica |
| 60 | Zhongzuo 93 | Jiangsu,China | 34.26 | 117.20 | 5.7 | this study | JS1995001 | 0.00001 | 0.99999 | Japonica |
| 61 | Huifeng 2 | Jiangsu,China | 33.38 | 120.13 | 5.9 | this study | C1509 | 0.00001 | 0.99999 | Japonica |
| 62 | Zhenghan 2hao | Henan,China | 34.76 | 113.65 | 5.1 | this study | GS2003031 | 0.00001 | 0.99999 | Japonica |
| 63 | Qiutianxiaoding | Japan | 35.68 | 139.69 | 5.5 | this study | H1654 | 0.00001 | 0.99999 | Japonica |
| 64 | Longjing 22 | Heilongjiang, China | 44.04 | 125.42 | 5.6 | this study | HS2008010 | 0.00001 | 0.99999 | Japonica |
| 65 | Longjing 25 | Heilongjiang, China | 44.04 | 125.42 | 5.8 | this study | HS2009009 | 0.00001 | 0.99999 | Japonica |
| 66 | Longjing 27 | Heilongjiang, China | 44.04 | 125.42 | 5.7 | this study | HS2009010 | 0.00001 | 0.99999 | Japonica |
| 67 | Songjing 10 | Heilongjiang, China | 41.42 | 119.52 | 5.5 | this study | HS2005005 | 0.00001 | 0.99999 | Japonica |
| 68 | Jindao 12 | Tianjin,China | 39.14 | 117.13 | 5.9 | this study | GS990004 | 0.00001 | 0.99999 | Japonica |
| 69 | Qingkong | Jiangsu,China | 32.04 | 118.78 | 5.7 | this study | Y1A01858 | 0.00001 | 0.99999 | Japonica |
| 70 | Youzhiyueguang | Jiangsu,China | 32.04 | 118.78 | 6 | this study | Y1A01876 | 0.00001 | 0.99999 | Japonica |
| 71 | Kangbingyueguang | Jiangsu,China | 35.68 | 139.69 | 5.8 | this study | H1524 | 0.00001 | 0.99999 | Japonica |
| 72 | Sujing 353 | Jiangsu,China | 31.32 | 120.62 | 6 | this study | C1511 | 0.00001 | 0.99999 | Japonica |
| 73 | Digludao | Jiangsu,China | 33.46 | 118.23 | 5.9 | this study | H1312 | 0.00001 | 0.99999 | Japonica |
| 74 | Dongnong 430 | Heilongjiang, China | 44.04 | 125.42 | 5.9 | this study | HS2009002 | 0.00001 | 0.99999 | Japonica |
| 75 | Ludao | Jiangsu,China | 32.04 | 118.78 | 5.8 | this study | H1508 | 0.00001 | 0.99999 | Japonica |
| 76 | Wanhuangdao | Jiangsu,China | 31.32 | 120.62 | 5.7 | this study | T815 | 0.00001 | 0.99999 | Japonica |
| 77 | Zaoshirihuangdao | Jiangsu,China | 31.32 | 120.62 | 5.8 | this study | T728 | 0.00001 | 0.99999 | Japonica |
| 78 | Luohanhuang | Jiangsu,China | 31.45 | 121.10 | 5.6 | this study | T560 | 0.00001 | 0.99999 | Japonica |
| 79 | Ebusinuodao | Jiangsu,China | 31.45 | 121.10 | 6.2 | this study | T386 | 0.00001 | 0.99999 | Japonica |
| 80 | Shenlenuo | Jiangsu,China | 31.39 | 120.95 | 6.1 | this study | T691 | 0.00001 | 0.99999 | Japonica |
| 81 | Yaxuenuo | Jiangsu,China | 31.45 | 121.10 | 5.9 | this study | T480 | 0.00001 | 0.99999 | Japonica |
| 82 | Xiepihuang | Jiangsu,China | 31.45 | 121.10 | 5.8 | this study | T203 | 0.00001 | 0.99999 | Japonica |
| 83 | Xiaobaidao | Jiangsu,China | 31.32 | 120.62 | 6.1 | this study | T208 | 0.00001 | 0.99999 | Japonica |
| 84 | Wumangyedao | Shanghai,China | 30.75 | 121.33 | 5.7 | this study | T335 | 0.00001 | 0.99999 | Japonica |
| 85 | Liaojing 287 | Liaoning,China | 41.8 | 123.38 | 3.1 | SRX509686 | H1634 | 0.00001 | 0.99999 | Japonica |
| 86 | Liming B | Liaoning,China | 41.8 | 123.24 | 3 | SRX509774 | A0386 | 0.00001 | 0.99999 | Japonica |
| 87 | Weiguo | Liaoning,China | 41.8 | 123.38 | 2.7 | SRX509684 | 05-00024 | 0.00001 | 0.99999 | Japonica |
| 88 | Jindao 1hao | Shanxi,China | 37.52 | 112.34 | 3 | SRX509714 | H1632 | 0.00001 | 0.99999 | Japonica |
| 89 | Zhonghua 8hao | Beijing, China | 39.54 | 116.28 | 3 | SRX509830 | ZD-02261 | 0.00001 | 0.99999 | Japonica |
| 90 | Laoguangtou 83 | Heilongjiang,China | 45.45 | 126.41 | 3 | SRX509674 | 07-00010 | 0.00001 | 0.99999 | Japonica |
| 91 | Zaoshunonghu 6 | Hunan, China | 28.11 | 113 | 3 | SRX509706 | A0132 | 0.00001 | 0.99999 | Japonica |
| 92 | Yangkenuo | Guizhou, China | 26.35 | 106.42 | 3 | SRX509846 | 22-04053 | 0.00001 | 0.99999 | Japonica |
| 93 | Guihuahuang | Jiangsu,China | 32.02 | 118.5 | 3 | SRX509710 | ZD-00587 | 0.00001 | 0.99999 | Japonica |
| 94 | Huangkezaogan | Jiangsu,China | 32.02 | 118.5 | 3 | SRX509733 | 09-00530 | 0.00001 | 0.99999 | Japonica |
| 95 | Sujing 2hao | Jiangsu,China | 32.02 | 118.5 | 3 | SRX509722 | ZD-00592 | 0.00001 | 0.99999 | Japonica |
| 96 | Ximaxian | Yunnan,China | 25.04 | 102.41 | 3 | SRX509769 | 21-05048 | 0.00001 | 0.99999 | Japonica |
| 97 | Putaohuang | Tianjin, China | 39.09 | 117.11 | 2.9 | SRX509847 | 29-00010 | 0.00001 | 0.99999 | Japonica |
| 98 | Gaoyangdiandao | Hebei,China | 38.02 | 114.28 | 3.1 | SRX509740 | 02-00210 | 0.00001 | 0.99999 | Japonica |
| 99 | Longhuamaohu | Hebei,China | 38.02 | 114.28 | 3 | SRX509690 | 02-00133 | 0.00001 | 0.99999 | Japonica |
| 100 | Zhonglou 1hao | Shanxi,China | 37.52 | 112.34 | 2.9 | SRX509692 | 04-00115 | 0.00001 | 0.99999 | Japonica |
| 101 | Feidongtangdao | Anhui, China | 37.51 | 117.18 | 3 | SRX509839 | 11-00529 | 0.00001 | 0.99999 | Japonica |
| 102 | Yelicanghua 2 | Hebei,China | 33.55 | 109.91 | 1.19 | SRX509693 | 02-00295-2 | 0.00001 | 0.99999 | Japonica |
| 103 | Yelicanghua 1 | Hebei,China | 38.02 | 114.28 | 3 | SRX509693 | 02-00295-1 | 0.00001 | 0.99999 | Japonica |
| 104 | Magunuo 1 | Guizhou, China | 26.35 | 106.42 | 3.1 | SRX509835 | 22-00513-1 | 0.00001 | 0.99999 | Japonica |
| 105 | Magunuo 2 | Guizhou, China | 26 | 107.86 | 0.83 | SRX509835 | 22-00513-2 | 0.00001 | 0.99999 | Japonica |
| 106 | Maguzi-2 | Shanxi,China | 34.16 | 108.54 | 3 | SRX509835 | 24-00195-2 | 0.00001 | 0.99999 | Japonica |
| 107 | Maguzi-1 | Shanxi,China | 33.55 | 109.91 | 2.22 | SRX509835 | 24-00195-1 | 0.00001 | 0.99999 | Japonica |
| 108 | Funingzipi | Hebei,China | 38.02 | 114.28 | 3 | SRX509681 | 02-00058 | 0.00001 | 0.99999 | Japonica |
| 109 | Cungu | Shanxi,China | 33.23 | 107.56 | 0.78 | SRX509795 | 22-00177 | 0.003932 | 0.996068 | Japonica |
| 110 | Xiangnuodao | Jiangsu,China | 32.02 | 118.5 | 5.7 | this study | T763 | 0.004946 | 0.995054 | Japonica |
| 111 | Huajing 6hao | Jiangsu,China | 33.50 | 119.15 | 6 | this study | SS200706 | 0.005045 | 0.994955 | Japonica |
| 112 | Dongzheng 1640 | Jiangsu,China | 33.28 | 118.85 | 5.8 | this study | C1515 | 0.005855 | 0.994145 | Japonica |
| 113 | Guangkexiangnuo | Guangxi,China | 22.48 | 108.2 | 3.1 | SRX510180 | 16-09350 | 0.006589 | 0.993411 | Japonica |
| 114 | Wandao 68 | Anhui,China | 31.86 | 117.27 | 5.9 | this study | WPS03010384 | 0.00852 | 0.99148 | Japonica |
| 115 | Yuyannuo | Yunnan,China | 25.04 | 102.41 | 3 | SRX509854 | 21-02235 | 0.00876 | 0.99124 | Japonica |
| 116 | Shanjiugu | Sichuan,China | 30.39 | 104.05 | 3 | SRX509743 | 20-03215 | 0.010095 | 0.989905 | Japonica |
| 117 | Dianjingyou 1hao | Yunnan,China | 25.04 | 102.41 | 3.1 | SRX510054 | H1648 | 0.011013 | 0.988987 | Japonica |
| 118 | Xiangjing 9407 | Jiangsu,China | 32.04 | 118.78 | 5.7 | this study | LS891061 | 0.012999 | 0.987001 | Japonica |
| 119 | Xiushui 115 | Zhejiang, China | 30.14 | 120.09 | 2.9 | SRX509712 | ZD-01559 | 0.014756 | 0.985244 | Japonica |
| 120 | Longnuo 3hao | Heilongjiang, China | 44.04 | 125.42 | 5.8 | this study | HS2009015 | 0.018332 | 0.981668 | Japonica |
| 121 | Zhongjing 212 | Jiangsu,China | 32.04 | 118.78 | 5.8 | this study | WS891061 | 0.018941 | 0.981059 | Japonica |
| 122 | Sihao 4259 | Jiangsu,China | 33.46 | 118.23 | 5.8 | this study | H1334 | 0.0193 | 0.9807 | Japonica |
| 123 | Wanlixian | Hunan, China | 28.11 | 113 | 2.9 | SRX509837 | 18-04906 | 0.019369 | 0.980631 | Japonica |
| 124 | Haobayong 1 | Yunnan,China | 25.04 | 102.41 | 3 | SRX509837 | 21-01853 | 0.019369 | 0.980631 | Japonica |
| 125 | Liusha 1hao | Guangxi,China | 22.48 | 108.2 | 3 | SRX509715 | ZD-00213 | 0.01937 | 0.98063 | Japonica |
| 126 | Longjing 28 | Heilongjiang, China | 44.04 | 125.42 | 5.7 | this study | HS2009011 | 0.019735 | 0.980265 | Japonica |
| 127 | Zhongjing 438 | Jiangsu,China | 32.04 | 118.78 | 5.6 | this study | C1514 | 0.020793 | 0.979207 | Japonica |
| 128 | Haolvguangzhan | Guizhou, China | 26.35 | 106.42 | 3 | SRX510187 | 22-04574 | 0.020987 | 0.979013 | Japonica |
| 129 | 863B | Jiangsu,China | 32.04 | 118.78 | 5.9 | this study | H1425 | 0.02349 | 0.97651 | Japonica |
| 130 | Sihao 4029 | Jiangsu,China | 33.46 | 118.23 | 5.2 | this study | H1333 | 0.02454 | 0.97546 | Japonica |
| 131 | Longdao 6hao | Heilongjiang, China | 44.04 | 125.42 | 5.8 | this study | HS2006004 | 0.031091 | 0.968909 | Japonica |
| 132 | Longdao 8hao | Heilongjiang, China | 44.04 | 125.42 | 5.7 | this study | HS2008019 | 0.031652 | 0.968348 | Japonica |
| 133 | Longdao 5hao | Heilongjiang, China | 44.04 | 125.42 | 5.8 | this study | HS2006003 | 0.032036 | 0.967964 | Japonica |
| 134 | Dongzhengwuyunjing 21 | Jiangsu,China | 33.28 | 118.85 | 5.9 | this study | SS200705-1 | 0.03317 | 0.96683 | Japonica |
| 135 | Feienuo | Guizhou, China | 26.35 | 106.42 | 3.1 | SRX510184 | 22-02356 | 0.03538 | 0.96462 | Japonica |
| 136 | Jindao 1007 | Tianjin,China | 39.14 | 117.13 | 5.6 | this study | GS2004043 | 0.039048 | 0.960952 | Japonica |
| 137 | Lianjing 4hao | Jiangsu,China | 34.59 | 119.16 | 5.9 | this study | SS200704 | 0.039074 | 0.960926 | Japonica |
| 138 | Sihao 4041 | Jiangsu,China | 33.46 | 118.23 | 5.8 | this study | H1338 | 0.044324 | 0.955676 | Japonica |
| 139 | Mudanjiang 28 | Heilongjiang, China | 44.58 | 129.60 | 5.6 | this study | HS2006006 | 0.049027 | 0.950973 | Japonica |
| 140 | Longdun 106 | Heilongjiang, China | 44.04 | 125.42 | 5.8 | this study | HS2008016 | 0.051933 | 0.948067 | Japonica |
| 141 | Nuohangu | Yunnan,China | 25.04 | 102.73 | 5.5 | this study | H1434 | 0.052126 | 0.947874 | Japonica |
| 142 | Yandao 6hao | Jiangsu,China | 33.38 | 120.13 | 5.1 | this study | SS200205 | 0.052237 | 0.947763 | Japonica |
| 143 | Huaidao 9hao | Jiangsu,China | 33.50 | 119.15 | 5.9 | this study | SS200607 | 0.053128 | 0.946872 | Japonica |
| 144 | Sihao 4081 | Jiangsu,China | 33.46 | 118.23 | 5.9 | this study | H1337 | 0.053383 | 0.946617 | Japonica |
| 145 | Lincangwazuhangu | Yunnan,China | 25.04 | 102.73 | 5.7 | this study | H1435 | 0.053526 | 0.946474 | Japonica |
| 146 | Haomake | Yunnan,China | 25.04 | 102.41 | 3 | SRX509843 | H1647 | 0.059274 | 0.940726 | Japonica |
| 147 | Biaojiyongzijing | Jiangsu,China | 32.04 | 118.78 | 5.8 | this study | H1427 | 0.069744 | 0.930256 | Japonica |
| 148 | M1004 | Jiangsu,China | 32.04 | 118.78 | 5.4 | this study | Y1A01861 | 0.070248 | 0.929752 | Japonica |
| 149 | A7444 | Jiangsu,China | 32.04 | 118.78 | 5.6 | this study | H1476 | 0.074845 | 0.925155 | Japonica |
| 150 | Niaw Ma-dan | Indonesia | 6.08 | 94.45 | 5.1 | this study | H1523 | 0.077984 | 0.922016 | Japonica |
| 151 | Xiangwanxian 17 | Hunan,China | 28.21 | 113.00 | 5.7 | this study | XS2008035 | 0.101909 | 0.898091 | Indica |
| 152 | Tijin | Jiangsu,China | 32.04 | 118.78 | 5.5 | this study | H1706 | 0.102659 | 0.897341 | Japonica |
| 153 | Huhui 628 | Hunan, China | 28.11 | 113 | 3 | SRX509687 | R0447 | 0.103335 | 0.896665 | Indica |
| 154 | Huanghuazhan | Hunan,China | 28.21 | 113.00 | 5.9 | this study | XS2007018 | 0.103606 | 0.896394 | Indica |
| 155 | Qutube-n | Indonesia | 6.08 | 94.45 | 5.8 | this study | H1520 | 0.106556 | 0.893444 | Japonica |
| 156 | Cbao | Anhui,China | 31.86 | 117.27 | 5.7 | this study | H1661 | 0.106651 | 0.893349 | Japonica |
| 157 | Ningjinghui 237 | Jiangsu,China | 32.04 | 118.78 | 5.8 | this study | H1374 | 0.108349 | 0.891651 | Japonica |
| 158 | Huajing 5hao | Jiangsu,China | 33.50 | 119.15 | 5.4 | this study | SS200505 | 0.115634 | 0.884366 | Japonica |
| 159 | Longdao 19hao | Heilongjiang, China | 41.42 | 119.52 | 5.7 | this study | HS2014003 | 0.121847 | 0.878153 | Japonica |
| 160 | BULUH BAWU | Indonesia | 6.08 | 94.45 | 5.5 | this study | 16481 | 0.122566 | 0.877434 | Japonica |
| 161 | Sihao 4082 | Jiangsu,China | 33.46 | 118.23 | 5.8 | this study | C1540 | 0.123566 | 0.876434 | Japonica |
| 162 | GUNDIL PUTIH | Indonesia | 6.08 | 94.45 | 6.5 | this study | 67205 | 0.127746 | 0.872254 | Japonica |
| 163 | Longdao 18hao | Heilongjiang, China | 44.04 | 125.42 | 5.8 | this study | HS2014005 | 0.14829 | 0.85171 | Japonica |
| 164 | Nonglinnuo 4hao | Jiangsu,China | 31.16 | 120.63 | 5.9 | this study | T762 | 0.148767 | 0.851233 | Japonica |
| 165 | CAMOR | Indonesia | 6.08 | 94.45 | 5.9 | this study | 10861 | 0.149575 | 0.850425 | Japonica |
| 166 | Gendjah Gempol | Indonesia | 6.08 | 94.45 | 5.8 | this study | 12483 | 0.177007 | 0.822993 | Admixture |
| 167 | Nanton 53 | Indonesia | 6.08 | 94.45 | 5.7 | this study | H1500 | 0.207399 | 0.792601 | Admixture |
| 168 | Ninghui 21 | Jiangsu,China | 32.02 | 118.5 | 3 | SRX509777 | R0337 | 0.211296 | 0.788704 | Admixture |
| 169 | Sihao 4031 | Jiangsu,China | 33.46 | 118.23 | 5.6 | this study | H1340 | 0.235474 | 0.764526 | Admixture |
| 170 | RODJOLELE | Indonesia | 6.08 | 94.45 | 5.3 | this study | 9909 | 0.273839 | 0.726161 | Admixture |
| 171 | Nannongjing 1R | Jiangsu,China | 32.04 | 118.78 | 5.8 | this study | H1372 | 0.279353 | 0.720647 | Admixture |
| 172 | PeiC122 | Hunan, China | 28.11 | 113 | 3 | SRX509709 | R0215 | 0.368026 | 0.631974 | Admixture |
| 173 | Sibeitichao 6 | Beijing, China | 39.9 | 116.4 | 3.1 | SRX510189 | ZD-03867 | 0.374141 | 0.625859 | Admixture |
| 174 | SH189 | Jiangsu,China | 33.46 | 118.23 | 5.8 | this study | Y1A01866 | 0.450445 | 0.549555 | Admixture |
| 175 | Yuzhenxiang | Hunan,China | 28.21 | 113.00 | 5.1 | this study | XS2009038 | 0.560083 | 0.439917 | Admixture |
| 176 | Chiguhong | Jiangsu,China | 31.16 | 120.63 | 5.1 | this study | H1382 | 0.604845 | 0.395155 | Admixture |
| 177 | Baoxintaihuqing | Jiangsu,China | 31.16 | 120.63 | 5.2 | this study | T834 | 0.636419 | 0.363581 | Admixture |
| 178 | Shentai B | Guangdong,China | 21.95 | 110.83 | 5.1 | this study | H1430 | 0.64311 | 0.35689 | Admixture |
| 179 | Malaihong | Jiangsu,China | 32.04 | 118.78 | 5.1 | this study | T050 | 0.693965 | 0.306035 | Admixture |
| 180 | Fanhaopi | Yunnan,China | 25.04 | 102.41 | 3 | SRX509744 | 21-03879 | 0.754214 | 0.245786 | Admixture |
| 181 | BOEGI BOERA | Indonesia | 6.08 | 94.45 | 5.4 | this study | 4165 | 0.760293 | 0.239707 | Admixture |
| 182 | Xibaizhan | Sichuan,China | 30.39 | 104.05 | 3 | SRX509793 | 20-01262 | 0.790921 | 0.209079 | Admixture |
| 183 | Xiangchuanwuxinbaimi | Jiangsu,China | 32.04 | 118.78 | 5.4 | this study | H1655 | 0.793026 | 0.206974 | Admixture |
| 184 | Shengtangqing | Jiangsu,China | 31.64 | 120.74 | 5.3 | this study | T759 | 0.813613 | 0.186387 | Admixture |
| 185 | IR661-1-2 | Philippines | 14.6 | 121 | 3.1 | SRX509822 | H1642 | 0.826401 | 0.173599 | Admixture |
| 186 | IR661-1-1 | Philippines | 14.6 | 121 | 3 | SRX509822 | H1633 | 0.826401 | 0.173599 | Admixture |
| 187 | Menjiadign 2 | Hainan, China | 20.2 | 110.3 | 3 | SRX509782 | 31-00042 | 0.841156 | 0.158844 | Admixture |
| 188 | Jiangpuchangliheimidao | Jiangsu,China | 32.04 | 118.78 | 5.5 | this study | H1492 | 0.847492 | 0.152508 | Indica |
| 189 | C418 | Beijing, China | 39.9 | 116.4 | 3.1 | SRX510012 | H1637 | 0.892113 | 0.107887 | Indica |
| 190 | Menjiagao 1 | Hainan, China | 20.2 | 110.3 | 3 | SRX509752 | 31-00032 | 0.896915 | 0.103085 | Indica |
| 191 | Baoxie-7B | Hunan, China | 28.11 | 113 | 3 | SRX509818 | A0464 | 0.900157 | 0.099843 | Indica |
| 192 | Yuedao 108 | Vietnam | 10.22 | 106.01 | 5.5 | this study | Y1A02355 | 0.907785 | 0.092215 | Indica |
| 193 | Yuedao 107 | Vietnam | 10.22 | 106.01 | 6.2 | this study | Y1A02368 | 0.910116 | 0.089884 | Indica |
| 194 | Zaoshuxiangheimi | Guangdong,China | 23.08 | 113.14 | 1.01 | ERS005992 | H1646 | 0.920778 | 0.079222 | Indica |
| 195 | Zaoshuxianghei | Guangxi,China | 22.48 | 108.2 | 3 | SRX509821 | ZD-02495 | 0.920778 | 0.079222 | Indica |
| 196 | Yuedao 43 | Vietnam | 10.22 | 106.01 | 6.2 | this study | Y1A02404 | 0.928825 | 0.071175 | Indica |
| 197 | Hongjiaozhan | Jiangsu,China | 31.32 | 120.62 | 5.4 | this study | T888 | 0.932842 | 0.067158 | Indica |
| 198 | Xiangxiandao 10hao | Hunan,China | 28.21 | 113.00 | 5.3 | this study | H1486 | 0.935761 | 0.064239 | Indica |
| 199 | L301B | Hunan, China | 28.11 | 113 | 2.8 | SRX509815 | A0096 | 0.946757 | 0.053243 | Indica |
| 200 | Shengyou 2hao | Guangdong,China | 23.05 | 112.44 | 5.3 | this study | YS1994004 | 0.947474 | 0.052526 | Indica |
| 201 | Qiyuexian | Guangxi,China | 22.48 | 108.2 | 3.1 | SRX509760 | 16-06887 | 0.949037 | 0.050963 | Indica |
| 202 | Nongxiang 18 | Hunan,China | 28.21 | 113.00 | 5.4 | this study | XS2010038 | 0.950072 | 0.049928 | Indica |
| 203 | Hongainuo | Guangxi,China | 22.48 | 108.2 | 3 | SRX509756 | 16-05252 | 0.950457 | 0.049543 | Indica |
| 204 | Nongxiang 25 | Hunan,China | 28.21 | 113.00 | 5.4 | this study | GS2001021 | 0.950631 | 0.049369 | Indica |
| 205 | Baikenuo | Jiangsu,China | 31.16 | 120.63 | 5.4 | this study | T354 | 0.950631 | 0.049369 | Indica |
| 206 | Nongxiang 21 | Hunan,China | 28.21 | 113.00 | 5.3 | this study | CNA200802496 | 0.952552 | 0.047448 | Indica |
| 207 | Zigu | Jiangsu,China | 32.04 | 118.78 | 5.4 | this study | H1437 | 0.954765 | 0.045235 | Indica |
| 208 | Jinxibai | Jiangxi,China | 28.41 | 115.52 | 3.1 | SRX509754 | 12-00589 | 0.957844 | 0.042156 | Indica |
| 209 | Lengshuinuo | Yunnan,China | 25.04 | 102.41 | 3.1 | SRX510183 | 21-01989 | 0.958047 | 0.041953 | Indica |
| 210 | Hongmangshajing | Jiangsu,China | 31.39 | 120.95 | 5.6 | this study | T630 | 0.958578 | 0.041422 | Indica |
| 211 | Zimi | Yunnan,China | 25.04 | 102.41 | 3 | SRX509856 | 21-01106 | 0.958752 | 0.041248 | Indica |
| 212 | Arias | Indonesia | 6.08 | 94.45 | 5.6 | this study | H1339 | 0.959773 | 0.040227 | Indica |
| 213 | Zaoxian 240 | Anhui, China | 37.51 | 117.18 | 3 | SRX509861 | ZD-03104 | 0.961993 | 0.038007 | Indica |
| 214 | Qingsiai 16B | Guangdong,China | 23.08 | 113.15 | 3 | SRX509683 | A0172 | 0.963562 | 0.036438 | Indica |
| 215 | Jiangnongzao 1hao | Jiangxi,China | 28.41 | 115.52 | 3 | SRX509775 | A0246 | 0.96413 | 0.03587 | Indica |
| 216 | Minbeiwanxian | Fujian, China | 26.05 | 119.18 | 3 | SRX509852 | 13-00737 | 0.965285 | 0.034715 | Indica |
| 217 | Zhenshan 97B | Zhejiang, China | 30.14 | 120.09 | 3 | SRX509803 | A0240 | 0.966161 | 0.033839 | Indica |
| 218 | Qitougu 1 | Yunnan,China | 25.04 | 102.41 | 3 | SRX509801 | 21-02171-1 | 0.967568 | 0.032432 | Indica |
| 219 | Qitougu 2 | Hunan,China | 29.41 | 111.09 | 0.95 | SRX509801 | 21-02171-2 | 0.967568 | 0.032432 | Indica |
| 220 | Jinbaoyin | Fujian, China | 26.05 | 119.18 | 3 | SRX509755 | 13-00723 | 0.969493 | 0.030507 | Indica |
| 221 | Chuan 5xian | Sichuan,China | 30.67 | 104.06 | 5.4 | this study | H1507 | 0.96956 | 0.03044 | Indica |
| 222 | Xiangdao | Henan,China | 32.62 | 114.38 | 0.88 | SRX509848 | ZD-00856 | 0.969816 | 0.030184 | Indica |
| 223 | Ⅱ-32B | Hunan,China | 28.21 | 113.00 | 5.3 | this study | A0050 | 0.969871 | 0.030129 | Indica |
| 224 | Zaoxian 14 | Anhui, China | 37.51 | 117.18 | 3 | SRX510025 | 11-00670 | 0.970448 | 0.029552 | Indica |
| 225 | Beizinuo | Yunnan,China | 25.04 | 102.41 | 3 | SRX509857 | 18-03997 | 0.970457 | 0.029543 | Indica |
| 226 | Chengnongshuijing | Sichuan,China | 30.39 | 104.05 | 2.8 | SRX509708 | ZD-03386 | 0.971318 | 0.028682 | Indica |
| 227 | Hainanxian R | Hainan,China | 19.52 | 109.57 | 5.5 | this study | H1504 | 0.97142 | 0.02858 | Indica |
| 228 | Dianrui 409B | Yunnan,China | 25.04 | 102.41 | 3 | SRX509685 | A0408 | 0.971848 | 0.028152 | Indica |
| 229 | Jinyou 1hao | Fujian, China | 26.05 | 119.18 | 3 | SRX509707 | ZD-02605 | 0.975298 | 0.024702 | Indica |
| 230 | IR36 | Philippines | 14.6 | 121 | 3.1 | SRX510159 | H1630 | 0.976514 | 0.023486 | Indica |
| 231 | Laozaogu | Yunnan,China | 25.04 | 102.41 | 3.1 | SRX510182 | 21-01744 | 0.977355 | 0.022645 | Indica |
| 232 | Chuan 6xian | Sichuan,China | 30.67 | 104.06 | 5.4 | this study | H1506 | 0.978057 | 0.021943 | Indica |
| 233 | Jinnante B | Hunan, China | 28.11 | 113 | 3 | SRX509680 | A0120 | 0.978395 | 0.021605 | Indica |
| 234 | Aimi | Jiangxi,China | 28.41 | 115.52 | 3 | SRX509836 | 12-02373 | 0.978765 | 0.021235 | Indica |
| 235 | Qitoubaigu 3 | Yunnan,China | 25.04 | 102.41 | 3 | SRX509736 | 21-02171-3 | 0.97924 | 0.02076 | Indica |
| 236 | Yuedao 22 | Vietnam | 10.22 | 106.01 | 5 | this study | Y1A02382 | 0.982133 | 0.017867 | Indica |
| 237 | Xiangai B | Jiangxi,China | 28.41 | 115.52 | 3 | SRX509779 | A0244 | 0.983773 | 0.016227 | Indica |
| 238 | Yizhixiang | Fujian, China | 26.05 | 119.18 | 3 | SRX509859 | 13-01301 | 0.985578 | 0.014422 | Indica |
| 239 | Xiangwanxian 3hao | Hunan, China | 28.11 | 113 | 3 | SRX509704 | ZD-02694 | 0.9859 | 0.0141 | Indica |
| 240 | Taizhongxianxuan 2 | Taiwan, China | 25.03 | 121.31 | 3 | SRX509726 | 30-00244 | 0.986058 | 0.013942 | Indica |
| 241 | Youzhan | Guizhou,China | 26.35 | 106.42 | 3 | SRX509849 | 22-02754 | 0.987516 | 0.012484 | Indica |
| 242 | Sanlicun | Shanxi,China | 34.16 | 108.54 | 3 | SRX509748 | 15-04016 | 0.988043 | 0.011957 | Indica |
| 243 | Yuetai B | Guangdong,China | 23.86 | 113.52 | 5.5 | this study | H1493 | 0.988399 | 0.011601 | Indica |
| 244 | Shufeng 101 | Sichuan,China | 30.39 | 104.05 | 2.7 | SRX509694 | ZD-00760 | 0.991811 | 0.008189 | Indica |
| 245 | Yuedao 24 | Vietnam | 10.22 | 106.01 | 6.2 | this study | Y1A02322 | 0.992901 | 0.007099 | Indica |
| 246 | Ziyeyingxiandao | Guangdong,China | 23.70 | 113.01 | 5.3 | this study | H1489 | 0.994938 | 0.005062 | Indica |
| 247 | 88B | Jiangsu,China | 32.02 | 118.5 | 3 | SRX509688 | A0598 | 0.995626 | 0.004374 | Indica |
| 248 | Qiuqianbai | Anhui, China | 37.51 | 117.18 | 3.1 | SRX510178 | 11-00389 | 0.995899 | 0.004101 | Indica |
| 249 | Yuedao 62 | Vietnam | 10.22 | 106.01 | 6.1 | this study | Y1A02414 | 0.996422 | 0.003578 | Indica |
| 250 | 80B | Hunan, China | 28.11 | 113 | 3 | SRX509772 | A0434 | 0.999149 | 0.000851 | Indica |
| 251 | Fengyouwan 8hao | Hunan,China | 28.21 | 113.00 | 5.4 | this study | YS2009001 | 0.99999 | 0.00001 | Indica |
| 252 | Diantun502xuanzao | Yunnan,China | 25.04 | 102.73 | 5.4 | this study | ZD-05551 | 0.99999 | 0.00001 | Indica |
| 253 | Shuanggui 1hao | Guangdong,China | 23.05 | 112.44 | 5.4 | this study | ZD-01065 | 0.99999 | 0.00001 | Indica |
| 254 | Yuedao 13 | Vietnam | 10.22 | 106.01 | 5.6 | this study | Y1A02320 | 0.99999 | 0.00001 | Indica |
| 255 | Yuedao 68 | Vietnam | 10.22 | 106.01 | 5.2 | this study | Y1A02418 | 0.99999 | 0.00001 | Indica |
| 256 | IR64 | Philippines | 14.6 | 121 | 5.2 | this study | H1502 | 0.99999 | 0.00001 | Indica |
| 257 | Yuedao 49 | Vietnam | 10.22 | 106.01 | 5.7 | this study | Y1A02408 | 0.99999 | 0.00001 | Indica |
| 258 | Yuedao 50 | Vietnam | 10.22 | 106.01 | 5.2 | this study | Y1A02331 | 0.99999 | 0.00001 | Indica |
| 259 | Yuedao 48 | Vietnam | 10.22 | 106.01 | 5.5 | this study | Y1A02407 | 0.99999 | 0.00001 | Indica |
| 260 | Yuedao 3 | Vietnam | 10.22 | 106.01 | 5 | this study | Y1A02370 | 0.99999 | 0.00001 | Indica |
| 261 | TAI-4 | Malaysia | 3.08 | 101.42 | 5.4 | this study | H1521 | 0.99999 | 0.00001 | Indica |
| 262 | Yuedao 66 | Vietnam | 10.22 | 106.01 | 5.2 | this study | Y1A02417 | 0.99999 | 0.00001 | Indica |
| 263 | Yuedao 12 | Vietnam | 10.22 | 106.01 | 6.1 | this study | Y1A02375 | 0.99999 | 0.00001 | Indica |
| 264 | IR20 | Philippines | 14.6 | 121 | 5.4 | this study | H1503 | 0.99999 | 0.00001 | Indica |
| 265 | Yuedao 109 | Vietnam | 10.22 | 106.01 | 6.2 | this study | Y1A02356 | 0.99999 | 0.00001 | Indica |
| 266 | Yuedao 55 | Vietnam | 10.22 | 106.01 | 6 | this study | Y1A02409 | 0.99999 | 0.00001 | Indica |
| 267 | Qimiaoxiang 2hao | Guangdong,China | 23.70 | 113.01 | 5.5 | this study | H1496 | 0.99999 | 0.00001 | Indica |
| 268 | Qing 7 | Jiangsu,China | 33.38 | 120.13 | 5.5 | this study | H1471 | 0.99999 | 0.00001 | Indica |
| 269 | Yuedao 9 | Vietnam | 10.22 | 106.01 | 6.1 | this study | Y1A02373 | 0.99999 | 0.00001 | Indica |
| 270 | Yuedao 37 | Vietnam | 10.22 | 106.01 | 5.5 | this study | Y1A02397 | 0.99999 | 0.00001 | Indica |
| 271 | Yuedao 61 | Vietnam | 10.22 | 106.01 | 6.1 | this study | Y1A02413 | 0.99999 | 0.00001 | Indica |
| 272 | Yuedao 41 | Vietnam | 10.22 | 106.01 | 5.3 | this study | Y1A02328 | 0.99999 | 0.00001 | Indica |
| 273 | Yuedao 32 | Vietnam | 10.22 | 106.01 | 6.1 | this study | Y1A02326 | 0.99999 | 0.00001 | Indica |
| 274 | IR112 | Philippines | 14.6 | 121 | 5.1 | this study | H1501 | 0.99999 | 0.00001 | Indica |
| 275 | Suwujing | Jiangsu,China | 31.78 | 119.95 | 5.4 | this study | SS201009 | 0.99999 | 0.00001 | Indica |
| 276 | LongtepuB | Fujian,China | 26.08 | 119.30 | 5.4 | this study | H1490 | 0.99999 | 0.00001 | Indica |
| 277 | Yimuhu | Jiangsu,China | 34.26 | 117.20 | 5.4 | this study | Y1A01857 | 0.99999 | 0.00001 | Indica |
| 278 | Longdao 14hao | Heilongjiang, China | 44.04 | 125.42 | 5.5 | this study | HS2012006 | 0.99999 | 0.00001 | Indica |
| 279 | Xu91075 | Jiangsu,China | 34.26 | 117.20 | 5.3 | this study | H1418 | 0.99999 | 0.00001 | Indica |
| 280 | Haobuqia | Jiangsu,China | 31.32 | 120.62 | 5.4 | this study | 21-00357 | 0.99999 | 0.00001 | Indica |
| 281 | SHN1 | Jiangsu,China | 33.46 | 118.23 | 5.5 | this study | Y1A01867 | 0.99999 | 0.00001 | Indica |
| 282 | Zhengdao 10hao | Jiangsu,China | 32.20 | 119.44 | 5.3 | this study | SS200710 | 0.99999 | 0.00001 | Indica |
| 283 | Zajiaohaigu | Hainan,China | 19.25 | 109.03 | 5.2 | this study | H1510 | 0.99999 | 0.00001 | Indica |
| 284 | Biwusheng | Yunnan,China | 25.04 | 102.41 | 3 | SRX509858 | 21-04506 | 0.99999 | 0.00001 | Indica |
| 285 | Xianggu 1 | Yunnan,China | 25.04 | 102.41 | 3 | SRX509768 | 21-01120-1 | 0.99999 | 0.00001 | Indica |
| 286 | Xianggu 2 | Yunnan,China | 22.95 | 117.64 | 0.93 | SRX509768 | 21-01120-2 | 0.99999 | 0.00001 | Indica |
| 287 | Honggu 3 | Yunnan,China | 25.04 | 102.41 | 3 | SRX509753 | 12-00121-3 | 0.99999 | 0.00001 | Indica |
| 288 | Laohongdao | Shanxi,China | 34.16 | 108.54 | 3 | ERS005998 | H1651 | 0.99999 | 0.00001 | Indica |
| 289 | Honggu 2 | Shanxi,China | 33.16 | 106.68 | 0.88 | SRX509798 | 12-00121-2 | 0.99999 | 0.00001 | Indica |
| 290 | Honggu 1 | Yunnan,China | 24.69 | 99.15 | 0.66 | ERS006390 | 12-00121-1 | 0.99999 | 0.00001 | Indica |
| 291 | Honggu 4 | Sichuan,China | 30.39 | 104.05 | 3 | SRX509753 | 12-00121-4 | 0.99999 | 0.00001 | Indica |
| 292 | Nanxiongzaoyou | Guangdong,China | 23.08 | 113.15 | 3 | SRX509820 | 15-03057 | 0.99999 | 0.00001 | Indica |
| 293 | Guichao 2hao | Guangdong,China | 23.08 | 113.15 | 3 | SRX509700 | H1645 | 0.99999 | 0.00001 | Indica |
| 294 | Xugunuo | Hunan, China | 28.11 | 113 | 3 | SRX509826 | 18-01903 | 0.99999 | 0.00001 | Indica |
| 295 | 76--1 | Jiangsu,China | 32.02 | 118.5 | 3 | SRX509825 | R0430 | 0.99999 | 0.00001 | Indica |
| 296 | Huangsiguizhan | Guangdong,China | 23.08 | 113.15 | 3 | SRX509703 | 15-00648 | 0.99999 | 0.00001 | Indica |
| 297 | Xiangwanxian 1hao | Hunan, China | 28.11 | 113 | 3 | SRX509828 | ZD-01423 | 0.99999 | 0.00001 | Indica |
| 298 | Taishannuo | Guangdong,China | 23.08 | 113.15 | 3 | SRX509699 | 12-02254 | 0.99999 | 0.00001 | Indica |
| 299 | Huke 3hao | Shanghai,China | 31.14 | 121.29 | 3 | SRX509701 | ZD-02017 | 0.99999 | 0.00001 | Indica |
| 300 | Aimakang | Sichuan,China | 30.39 | 104.05 | 3 | SRX509831 | ZD-00747 | 0.99999 | 0.00001 | Indica |
| 301 | Nanjing 11hao | Jiangsu,China | 32.02 | 118.5 | 3 | SRX509809 | ZD-00560 | 0.99999 | 0.00001 | Indica |
| 302 | Bawangbian 1 | Hubei,China | 30 | 114 | 3 | SRX509717 | 17-01470 | 0.99999 | 0.00001 | Indica |
| 303 | Xiangaizao 10hao | Hunan, China | 28.11 | 113 | 3 | SRX509679 | ZD-01402 | 0.99999 | 0.00001 | Indica |
| 304 | Zhuzhen B | Hunan, China | 28.11 | 113 | 3.1 | SRX509817 | A0060 | 0.99999 | 0.00001 | Indica |
| 305 | Gzhenshan 97B | Sichuan,China | 30.39 | 104.05 | 3 | SRX509819 | A0596 | 0.99999 | 0.00001 | Indica |
| 306 | Chaoyang 1hao | Hunan, China | 28.11 | 113 | 3 | SRX509814 | A0086 | 0.99999 | 0.00001 | Indica |
| 307 | Erjiunan 1hao | Zhejiang, China | 30.14 | 120.09 | 3 | SRX509813 | ZD-00474 | 0.99999 | 0.00001 | Indica |
| 308 | Guangluai 15 | Guangxi,China | 22.48 | 108.2 | 3 | SRX509816 | ZD-01266 | 0.99999 | 0.00001 | Indica |
| 309 | Aijiaonante | Zhejiang,China | 30.14 | 120.09 | 2.9 | SRX509677 | ZD-01512-3 | 0.99999 | 0.00001 | Indica |
| 310 | Chengduai 3hao | Sichuan,China | 30.39 | 104.05 | 3 | SRX509695 | ZD-00743 | 0.99999 | 0.00001 | Indica |
| 311 | Gongju 73 | Yunnan,China | 25.04 | 102.41 | 3 | SRX509697 | 21-01899 | 0.99999 | 0.00001 | Indica |
| 312 | Jiabala | Guangxi,China | 22.48 | 108.2 | 3 | SRX509698 | 26-00008 | 0.99999 | 0.00001 | Indica |
| 313 | Xiangzaoxian 7hao | Hunan, China | 28.11 | 113 | 3 | SRX509771 | ZD-02715 | 0.99999 | 0.00001 | Indica |
| 314 | Chenwan 3hao | Hunan, China | 28.11 | 113 | 3 | SRX509833 | ZD-00358 | 0.99999 | 0.00001 | Indica |
| 315 | Zhenxian 232 | Jiangsu,China | 32.02 | 118.5 | 3 | SRX509833 | ZD-02944 | 0.99999 | 0.00001 | Indica |
| 316 | Benbanggu-2 | Yunnan,China | 25.04 | 102.41 | 3 | SRX509735-2 | 21-00785-2 | 0.99999 | 0.00001 | Indica |
| 317 | Benbanggu-1 | Yunnan,China | 25.04 | 102.41 | 3 | SRX509735-1 | 21-00785-1 | 0.99999 | 0.00001 | Indica |
| 318 | Ersiniu | Guangdong,China | 23.08 | 113.15 | 3 | SRX509731 | H1644 | 0.99999 | 0.00001 | Indica |
| 319 | Zegu | Guizhou, China | 26.35 | 106.42 | 3 | SRX509794 | 22-00040 | 0.99999 | 0.00001 | Indica |
| 320 | Hanmadao 2 | Henan,China | 34.48 | 113.42 | 3 | SRX509705 | 19-00205-2 | 0.99999 | 0.00001 | Indica |
| 321 | Hanmadao 1 | Henan,China | 34.48 | 113.42 | 3 | SRX509738 | 19-00205-1 | 0.99999 | 0.00001 | Indica |
| 322 | Mamagu 1 | Sichuan,China | 30.39 | 104.05 | 3 | SRX509766 | 20-01452-1 | 0.99999 | 0.00001 | Indica |
| 323 | Mamagu 2 | Sichuan,China | 30.39 | 104.05 | 3 | SRX509766 | 20-01452-2 | 0.99999 | 0.00001 | Indica |
| 324 | Maweizhan 4 | Guizhou, China | 26.35 | 106.42 | 3 | SRX509855 | 22-01615-4 | 0.99999 | 0.00001 | Indica |
| 325 | Maweizhan 3 | Yunnan,China | 25.1 | 104.91 | 1.11 | SRX509855 | 22-01615-3 | 0.99999 | 0.00001 | Indica |
| 326 | Maweizhan 2 | Guangdong,China | 25.08 | 113.91 | 0.87 | SRX509855 | 22-01615-2 | 0.99999 | 0.00001 | Indica |
| 327 | Maweizhan 1 | Hunan,China | 24.97 | 111.79 | 0.97 | SRX509855 | 22-01615-1 | 0.99999 | 0.00001 | Indica |
| 328 | Maweizhan 5 | Hubei,China | 30.35 | 114.17 | 0.89 | SRX509855 | 22-01615-5 | 0.99999 | 0.00001 | Indica |
| 329 | Maweizhan 6 | Zhejiang, China | 29.61 | 119.05 | 1.92 | SRX509855 | 22-01615-6 | 0.99999 | 0.00001 | Indica |
| 330 | Hengxianliangchun 2 | Guangxi,China | 22.48 | 108.2 | 3 | SRX509840 | 16-00163-2 | 0.99999 | 0.00001 | Indica |
| 331 | Hengxianliangchun 1 | Guangxi,China | 22.48 | 108.2 | 3 | SRX509840 | 16-00163-1 | 0.99999 | 0.00001 | Indica |
| 332 | Leihuozhan | Anhui, China | 37.51 | 117.18 | 3 | SRX509841 | 11-00403 | 0.99999 | 0.00001 | Indica |
| 333 | Liuyezhan | Hubei,China | 30 | 114 | 3 | SRX509786 | 17-00524 | 0.99999 | 0.00001 | Indica |
| 334 | Zhongnong 4hao | Sichuan,China | 30.39 | 104.05 | 3 | SRX509851 | 20-02821 | 0.99999 | 0.00001 | Indica |
| 335 | Meihuanuo | Sichuan,China | 30.39 | 104.05 | 3 | SRX509749 | 20-02073 | 0.99999 | 0.00001 | Indica |
| 336 | Xiaobaimi | Guizhou, China | 26.35 | 106.42 | 3 | SRX509860 | 22-04637 | 0.99999 | 0.00001 | Indica |
| 337 | Baikehualuo | Guangdong,China | 23.08 | 113.15 | 3 | SRX509785 | 15-013168 | 0.99999 | 0.00001 | Indica |
| 338 | Yienchangtanqingzhan | Hubei,China | 30 | 114 | 3 | SRX509789 | 17-00966 | 0.99999 | 0.00001 | Indica |
| 339 | Nantehao 3 | Hainan,China | 19.36 | 110.1 | 1.11 | SRX509730-3 | ZD-01512-1 | 0.99999 | 0.00001 | Indica |
| 340 | Nantehao 2 | Jiangxi,China | 28.41 | 115.52 | 3 | SRX509730-2 | ZD-01512-4 | 0.99999 | 0.00001 | Indica |
| 341 | Nantehao 1 | Jiangxi,China | 28.41 | 115.52 | 1.12 | SRX509730-1 | ZD-01512-2 | 0.99999 | 0.00001 | Indica |
| 342 | Jiefangxian | Jiangxi,China | 28.41 | 115.52 | 3 | SRX509783 | 12-00644 | 0.99999 | 0.00001 | Indica |
| 343 | Sankecun | Hubei,China | 30.35 | 114.17 | 0.81 | SRX509696 | 20-03053 | 0.99999 | 0.00001 | Indica |
| 344 | Dongtingwanxian | Hubei,China | 30 | 114 | 3.1 | SRX509718 | 17-00502 | 0.99999 | 0.00001 | Indica |
| 345 | Sanbaili-3 | Tianjin, China | 39.09 | 117.11 | 3 | SRX509713-3 | 12-01446-3 | 0.99999 | 0.00001 | Indica |
| 346 | Sanbaili-2 | Tianjin, China | 39.09 | 117.11 | 3 | SRX509713-2 | 12-01446-2 | 0.99999 | 0.00001 | Indica |
| 347 | Sanbaili-1 | Hunan, China | 28.11 | 113 | 3 | SRX509713-1 | 12-01446-1 | 0.99999 | 0.00001 | Indica |
| 348 | Jinnante 43B | Hunan, China | 28.11 | 113 | 3 | SRX509770 | A0016 | 0.99999 | 0.00001 | Indica |
| 349 | Wukezhan | Fujian, China | 26.05 | 119.18 | 3 | SRX509850 | 13-01006 | 0.99999 | 0.00001 | Indica |
| 350 | Gu 154 | Hunan, China | 28.11 | 113 | 3 | SRX509776 | R0004 | 0.99999 | 0.00001 | Indica |
| 351 | 9311 | Hunan, China | 28.11 | 113 | 3 | SRX509806 | H1650 | 0.99999 | 0.00001 | Indica |
| 352 | JWR221 | Jiangsu,China | 32.02 | 118.5 | 3 | SRX509832 | R0604 | 0.99999 | 0.00001 | Indica |
| 353 | Huakeheinuo | Hainan,China | 19.05 | 109.83 | 1.86 | ERS039317 | H1643 | 0.99999 | 0.00001 | Indica |

^a^ ERS and SRX indicated the NCBI sequence Read Archive (SRA) accession number. b The word in black type indicated that these materials were saved by our lab; T showed the materials were from Taihu Lake; H showed that the materials were introduced by Hong; C showed that the materials were always saved by the lab. The word in red type indicated that these materials were from the National germplasm resource base. The word in blue type indicated that these materials were the approved variety; CNA indicated the No. of variety right. GS, the abbreviation of guoshendao. HS, the abbreviation of heishendao. These indicated that the accessions were approved by Heilongjiang province. JS, the abbreviation of jishendao. These indicated that the accessions were approved by Jilin province. LS, the abbreviation of liaoshendao. These indicated that the accessions were approved by Liaoning province. SS, the abbreviation of sushendao. These indicated that the accessions were approved by Jiangsu province. SZS, the abbreviation of suzhongshen. These indicated the accessions were approved by Jiangsu province. WPS, the abbreviation of wanpinshen. These indicated that the accessions were approved by Anhui province. WS, the abbreviation of wanshendao. These indicated that the accessions were approved by Anhui province. XS, the abbreviation of xiangshendao. These indicated that the accessions were approved by Hunan province. YS, the abbreviation of yueshendao. These indicated that the accessions were approved by Guangdong province.
