# Supplementary material for: OsSYL2 AA, an allele identified by gene‐based association, increases style length in rice (Oryza sativa L.)
Source: Plant J. 2020 Oct 30;104(6):1491–503. doi: 10.1111/tpj.15013 (PMC7821000; doi:10.1111/tpj.15013)
Supplement: Supplementary file 3 — Table S2. Basic statistics of the three stigma characteristics in each environment. [file TPJ-104-1491-s003.docx]

**Table S2.** Basic statistics of the three stigma characteristics in each environment.

| Trait | Environment | Mean | Maximum | Minimum | SD | CV/% |
| --- | --- | --- | --- | --- | --- | --- |
| STL/mm | E1 | 1.17 | 2.06 | 0.73 | 0.18 | 15.38 |
|  | E2 | 1.19 | 2.08 | 0.72 | 0.18 | 15.13 |
|  | E3 | 1.16 | 2.08 | 0.74 | 0.17 | 14.66 |
|  | E4 | 1.16 | 1.98 | 0.77 | 0.16 | 13.79 |
|  | E5 | 1.17 | 2.03 | 0.76 | 0.17 | 14.53 |
|  | E6 | 1.17 | 2.01 | 0.74 | 0.17 | 14.53 |
|  | Mean | 1.17 | 2.04 | 0.74 | 0.17 | 14.67 |
|  |  |  |  |  |  |  |
| SYL/mm | E1 | 0.60 | 1.19 | 0.41 | 0.11 | 18.33 |
|  | E2 | 0.62 | 1.24 | 0.43 | 0.12 | 19.35 |
|  | E3 | 0.62 | 1.32 | 0.44 | 0.12 | 19.35 |
|  | E4 | 0.61 | 1.14 | 0.39 | 0.11 | 18.03 |
|  | E5 | 0.63 | 1.31 | 0.43 | 0.12 | 19.05 |
|  | E6 | 0.62 | 1.28 | 0.42 | 0.12 | 19.35 |
|  | Mean | 0.62 | 1.25 | 0.42 | 0.12 | 18.91 |
|  |  |  |  |  |  |  |
| TSSL/mm | E1 | 1.78 | 2.72 | 1.27 | 0.23 | 12.92 |
|  | E2 | 1.80 | 2.68 | 1.31 | 0.24 | 13.33 |
|  | E3 | 1.78 | 2.63 | 1.26 | 0.23 | 12.92 |
|  | E4 | 1.77 | 2.60 | 1.22 | 0.22 | 12.43 |
|  | E5 | 1.79 | 2.68 | 1.30 | 0.23 | 12.85 |
|  | E6 | 1.80 | 2.64 | 1.23 | 0.24 | 13.33 |
|  | Mean | 1.79 | 2.66 | 1.27 | 0.23 | 12.96 |

STL, stigma length; SYL, style length; TSSL, the sum of stigma and style length; SD, standard deviation, which was calculated based on the measured values of the traits from two replicates; CV, coefficient of variation. E1, environment 1, Nanjing 2014; E2, environment 2, Nanjing 2015; E3, environment 3, Nanjing 2016; E4, environment 4, Yuanyang 2014; E5, environment 5, Yuanyang 2015; E6, environment 6, Yuanyang 2016.
