# Supplementary material for: OsSYL2 AA, an allele identified by gene‐based association, increases style length in rice (Oryza sativa L.)
Source: Plant J. 2020 Oct 30;104(6):1491–503. doi: 10.1111/tpj.15013 (PMC7821000; doi:10.1111/tpj.15013)
Supplement: Supplementary file 4 — Table S3. The results of joint analysis of variance for the three stigma characteristics. [file TPJ-104-1491-s004.docx]

**Table S3.** The results of joint analysis of variance for the three stigma characteristics.

| Characteristics | Source of variation | df | SS | MS | *F*-value | *F*_0.05_ | *F*_0.01_ |
| --- | --- | --- | --- | --- | --- | --- | --- |
| STL/mm | Among genotypes | 352 | 120.5250 | 0.3424 | 153.32^**^ | 1.18 | 1.26 |
|  | Among environments | 5 | 0.3012 | 0.0602 | 2.56 | 4.39 | 8.75 |
|  | Genotype × Environment | 1760 | 3.9305 | 0.0022 | 0.94 | 1.12 | 1.17 |
| SYL/mm | Among genotypes | 352 | 53.0352 | 0.1507 | 130.81^**^ | 1.18 | 1.26 |
|  | Among environments | 5 | 0.2252 | 0.0450 | 2.26 | 4.39 | 8.75 |
|  | Genotype × Environment | 1760 | 2.0272 | 0.0012 | 0.90 | 1.12 | 1.17 |
| TSSL/mm | Among genotypes | 352 | 221.7228 | 0.6299 | 178.14^**^ | 1.18 | 1.26 |
|  | Among environments | 5 | 0.5185 | 0.1037 | 3.95 | 4.39 | 8.75 |
|  | Genotype × Environment | 1760 | 6.2231 | 0.0035 | 0.95 | 1.12 | 1.17 |

df, degrees of freedom; SS, sum of squares; MS, mean square. ^**^ Significant differences at *P* < 0.01. STL, stigma length; SYL, style length; TSSL, the sum of stigma and style length.
