# Supplementary material for: OsSYL2 AA, an allele identified by gene‐based association, increases style length in rice (Oryza sativa L.)
Source: Plant J. 2020 Oct 30;104(6):1491–503. doi: 10.1111/tpj.15013 (PMC7821000; doi:10.1111/tpj.15013)
Supplement: Supplementary file 5 — Table S4. The distribution of the significant association single‐nucleotide polymorphism loci detected in the rice population composed of 353 accessions in the six environments. [file TPJ-104-1491-s005.docx]

**Table S4**. The distribution of the significant association SNP loci detected in the rice population composed of 353 accession in the six environments.

| Trait | SNP site | The number of environments detected | E1 | | | E2 | | | E3 | | | E4 | | | E5 | | | E6 | | |
| --- | --- | --- | --- | --- | --- | --- | --- | --- | --- | --- | --- | --- | --- | --- | --- | --- | --- | --- | --- | --- |
|  |  |  | *P* value | R2% | FDR | *P* value | R2% | FDR | *P* value | R2% | FDR | *P* value | R2% | FDR | *P* value | R2% | FDR | *P* value | R2% | FDR |
| STL | 14511886 | 4 | 1.91E-06 |  | 2.27E-06 |  |  |  |  |  |  | 1.47E-06 | 4.01 | 2.86E-06 | 1.97E-06 | 3.88 | 3.08E-06 | 1.25E-06 | 4.06 | 2.07E-06 |
|  | 22114062 | 4 | 2.21E-06 | 3.80 | 3.18E-06 |  |  |  |  |  |  | 2.13E-06 | 3.84 | 3.21E-06 | 6.66E-07 | 4.20 | 1.03E-06 | 9.82E-07 | 4.10 | 1.72E-06 |
|  | 24396164 | 4 |  |  |  | 5.55E-07 | 4.26 | 5.60E-06 |  |  |  | 9.73E-08 | 4.88 | 3.57E-07 | 1.54E-07 | 4.78 | 2.56E-07 | 3.93E-08 | 5.20 | 3.45E-07 |
|  | 14401388 | 5 | 1.95E-06 | 3.90 | 2.73E-06 | 2.10E-06 | 3.85 | 3.20E-06 |  |  |  | 9.67E-07 | 4.10 | 1.79E-06 | 3.46E-07 | 4.39 | 7.69E-07 | 8.95E-08 | 4.93 | 6.90E-07 |
|  |  |  |  |  |  |  |  |  |  |  |  |  |  |  |  |  |  |  |  |  |
| SYL | 11281296 | 4 |  |  |  | 1.79E-07 | 4.71 | 8.01E-07 | 1.86E-06 | 3.90 | 3.24E-06 |  |  |  | 2.20E-06 | 3.83 | 2.64E-06 | 6.77E-07 | 4.20 | 1.36E-06 |
|  | 25139654 | 5 |  |  |  | 7.74E-07 | 4.17 | 2.05E-06 | 3.59E-07 | 4.42 | 1.04E-06 | 2.60E-06 | 3.75 | 3.65E-06 | 2.90E-07 | 4.45 | 7.11E-07 | 8.52E-07 | 4.15 | 1.55E-06 |
|  | 31869936 | 4 |  |  |  | 4.82E-07 | 4.30 | 1.60E-06 | 2.34E-08 | 5.43 | 2.42E-07 |  |  |  | 2.77E-06 | 3.73 | 3.05E-06 | 2.67E-07 | 4.47 | 6.33E-07 |
|  | 34395535 | 5 | 6.27E-07 | 4.20 | 1.62E-06 |  |  |  | 3.46E-08 | 5.28 | 3.62E-07 | 1.42E-07 | 4.80 | 6.35E-07 | 7.84E-08 | 4.96 | 2.54E-07 | 3.28E-08 | 5.29 | 1.27E-07 |
|  | 30510492 | 6 | 4.70E-07 | 4.30 | 1.37E-06 | 8.85E-07 | 4.15 | 2.18E-06 | 3.30E-07 | 4.43 | 9.18E-07 | 2.87E-07 | 4.45 | 9.52E-07 | 8.15E-08 | 4.96 | 3.05E-07 | 1.13E-07 | 4.85 | 4.11E-07 |
|  | 30512579 | 5 | 1.16E-07 | 4.84 | 4.27E-07 |  |  |  | 1.40E-06 | 4.03 | 2.44E-06 | 2.75E-06 | 3.73 | 3.97E-06 | 5.17E-08 | 5.13 | 2.03E-07 | 2.55E-07 | 4.50 | 6.01E-07 |
|  | 30596777 | 6 | 1.44E-07 | 4.80 | 4.87E-06 | 2.85E-07 | 4.45 | 9.94E-07 | 1.62E-06 | 3.93 | 2.81E-06 | 5.22E-08 | 5.12 | 4.76E-07 | 8.62E-09 | 5.85 | 5.08E-08 | 3.36E-08 | 5.28 | 1.58E-07 |
|  | 30606084 | 5 | 4.04E-08 | 5.20 | 8.55E-08 | 3.02E-07 | 4.44 | 1.03E-06 | 1.52E-06 | 4.01 | 2.66E-06 |  |  |  | 2.70E-07 | 4.47 | 6.60E-07 | 2.27E-07 | 4.55 | 5.06E-07 |
|  | 30620061 | 5 | 7.84E-07 | 4.17 | 2.05E-06 |  |  |  | 2.95E-06 | 3.71 | 4.52E-06 | 2.10E-06 | 3.85 | 3.33E-06 | 1.69E-07 | 4.72 | 5.58E-07 | 5.76E-07 | 4.22 | 1.23E-06 |
|  | 16690429 | 4 | 2.39E-06 | 3.78 | 5.98E-06 | 1.73E-06 | 3.91 | 5.74E-06 | 5.00E-08 | 5.12 | 2.37E-07 |  |  |  |  |  |  | 2.31E-06 | 3.79 | 2.75E-06 |
|  | 16692834 | 4 |  |  |  | 1.63E-07 | 4.72 | 7.37E-07 | 1.56E-06 | 3.94 | 2.72E-06 |  |  |  | 2.85E-06 | 3.72 | 3.15E-06 | 2.95E-06 | 3.71 | 3.48E-06 |
|  | 16708049 | 5 | 1.47E-06 | 4.01 | 2.65E-06 | 2.04E-06 | 3.85 | 3.30E-06 | 1.38E-06 | 4.03 | 2.39E-06 | 2.38E-06 | 3.78 | 3.49E-06 | 1.53E-06 | 4.01 | 2.23E-06 |  |  |  |
|  | 16733441 | 4 |  |  |  | 4.55E-08 | 5.19 | 3.84E-07 | 1.32E-06 | 4.05 | 2.37E-06 | 3.94E-08 | 5.20 | 1.59E-07 | 1.22E-08 | 5.75 | 1.02E-07 |  |  |  |
|  | 16881568 | 5 | 1.02E-07 | 4.86 | 9.40E-07 | 9.49E-08 | 4.91 | 5.45E-07 | 3.11E-07 | 4.44 | 8.93E-07 |  |  |  | 8.11E-07 | 4.16 | 1.17E-06 | 1.67E-06 | 3.93 | 2.34E-06 |
|  | 30732321 | 4 |  |  |  | 1.55E-06 | 3.94 | 2.72E-06 | 5.77E-07 | 4.22 | 1.43E-06 | 1.55E-06 | 4.01 | 2.54E-06 | 2.23E-06 | 3.80 | 2.69E-06 |  |  |  |
|  | 9290646 | 4 |  |  |  | 1.51E-07 | 4.78 | 7.05E-07 | 2.32E-08 | 5.43 | 2.17E-07 |  |  |  | 1.32E-06 | 4.05 | 2.08E-06 | 1.23E-06 | 4.06 | 1.99E-06 |
|  | 1153212 | 5 |  |  |  | 2.54E-07 | 4.50 | 9.29E-07 | 1.32E-08 | 5.71 | 1.45E-08 | 1.82E-06 | 3.92 | 3.17E-06 | 5.71E-07 | 4.22 | 9.64E-07 | 3.68E-07 | 4.38 | 8.54E-07 |
|  | 26598751 | 4 |  |  |  |  |  |  | 1.56E-06 | 3.94 | 2.73E-06 | 1.81E-06 | 3.92 | 3.02E-06 | 8.81E-07 | 4.15 | 1.37E-06 | 1.25E-06 | 4.06 | 2.09E-06 |
|  | 17486934 | 4 | 3.52E-07 | 4.42 | 9.40E-07 | 1.32E-08 | 5.71 | 1.92E-07 | 2.75E-08 | 5.35 | 3.38E-07 |  |  |  |  |  |  | 1.91E-06 | 3.90 | 2.53E-06 |
|  | 3094153 | 4 |  |  |  | 2.55E-08 | 5.38 | 2.88E-07 | 1.08E-06 | 4.07 | 1.95E-06 |  |  |  | 2.26E-07 | 4.55 | 6.09E-07 | 6.78E-07 | 4.20 | 1.39E-06 |
|  |  |  |  |  |  |  |  |  |  |  |  |  |  |  |  |  |  |  |  |  |
| TSSL | 16183113 | 6 | 5.73E-07 | 4.22 | 1.25E-06 | 2.24E-07 | 4.55 | 4.17E-07 | 3.87E-08 | 5.20 | 3.33E-07 | 4.55E-08 | 5.19 | 2.01E-07 | 4.99E-08 | 5.18 | 4.62E-07 | 4.66E-08 | 5.19 | 2.65E-07 |
|  | 16184264 | 6 | 2.51E-06 | 3.74 | 3.75E-06 | 2.99E-06 | 3.71 | 3.96E-06 | 2.10E-06 | 3.79 | 2.67E-06 | 1.00E-07 | 4.90 | 4.10E-07 | 6.70E-08 | 5.01 | 6.36E-07 | 7.36E-08 | 4.96 | 4.64E-07 |
|  | 16197239 | 5 | 2.25E-06 | 3.79 | 3.63E-06 |  |  |  | 7.23E-07 | 4.18 | 2.00E-06 | 7.93E-08 | 4.94 | 3.12E-07 | 8.84E-07 | 4.15 | 2.02E-06 | 4.66E-08 | 5.19 | 2.65E-07 |
|  | 16197256 | 5 | 1.92E-06 | 3.90 | 3.00E-06 |  |  |  | 2.41E-06 | 3.78 | 3.17E-06 | 1.52E-07 | 4.79 | 6.00E-07 | 1.13E-06 | 4.07 | 2.43E-06 | 2.00E-07 | 4.60 | 8.61E-07 |
|  | 16202578 | 6 | 1.33E-06 | 4.05 | 2.31E-06 | 1.28E-06 | 4.06 | 1.88E-06 | 6.70E-07 | 4.20 | 1.83E-06 | 2.38E-07 | 4.53 | 9.00E-07 | 6.08E-08 | 5.08 | 5.20E-07 | 4.07E-08 | 5.24 | 1.99E-07 |
|  | 16256300 | 5 | 2.23E-06 | 3.79 | 3.51E-06 | 2.42E-06 | 3.78 | 3.13E-06 |  |  |  | 3.23E-07 | 4.44 | 1.12E-06 | 4.65E-07 | 4.30 | 1.33E-06 | 2.31E-06 | 3.79 | 4.11E-06 |
|  | 16258184 | 4 |  |  |  |  |  |  | 2.29E-06 | 3.79 | 3.00E-06 | 2.36E-07 | 4.53 | 8.02E-07 | 4.22E-07 | 4.32 | 1.27E-06 | 5.41E-08 | 5.11 | 3.31E-07 |
|  | 16686373 | 6 | 2.98E-06 | 3.71 | 4.10E-06 | 3.13E-06 | 3.68 | 4.06E-06 | 6.67E-07 | 4.20 | 1.33E-06 | 2.15E-07 | 4.57 | 7.13E-07 | 9.40E-08 | 4.91 | 8.09E-07 | 5.43E-08 | 5.11 | 3.97E-07 |
|  | 16691998 | 5 | 4.85E-07 | 4.30 | 1.00E-06 | 1.10E-06 | 4.07 | 1.67E-06 |  |  |  | 1.30E-06 | 4.05 | 2.10E-06 | 4.07E-08 | 5.24 | 3.47E-07 | 2.53E-07 | 4.50 | 9.27E-07 |
|  | 16720463 | 4 | 2.22E-06 | 3.79 | 3.38E-06 | 1.41E-06 | 4.03 | 2.29E-06 |  |  |  | 2.53E-06 | 3.74 | 3.60E-06 | 3.10E-06 | 3.70 | 4.34E-06 |  |  |  |
|  | 16733441 | 4 | 2.51E-06 | 3.74 | 3.75E-06 |  |  |  |  |  |  | 1.15E-07 | 4.84 | 5.00E-07 | 2.93E-08 | 5.33 | 2.31E-07 | 5.43E-08 | 5.11 | 3.97E-07 |
|  | 16878104 | 5 | 6.07E-07 | 4.20 | 1.38E-06 | 2.82E-06 | 3.72 | 3.44E-06 | 6.68E-07 | 4.20 | 1.50E-06 | 1.22E-06 | 4.06 | 1.80E-06 |  |  |  | 1.45E-07 | 4.80 | 7.28E-07 |
|  | 17019509 | 4 | 4.87E-07 | 4.30 | 1.13E-06 | 1.29E-06 | 4.06 | 1.98E-06 |  |  |  |  |  |  | 3.31E-07 | 4.44 | 9.83E-07 | 1.23E-07 | 4.83 | 6.62E-07 |
|  | 16692729 | 4 |  |  |  | 1.30E-07 | 4.82 | 3.13E-07 | 2.93E-08 | 5.33 | 2.93E-08 |  |  |  | 4.99E-08 | 5.18 | 4.62E-07 | 5.57E-07 | 4.26 | 1.32E-06 |
|  | 20459617 | 4 | 2.00E-07 | 4.67 | 5.00E-07 | 2.13E-07 | 4.57 | 4.17E-07 |  |  |  |  |  |  | 2.06E-06 | 3.85 | 3.47E-06 | 1.66E-06 | 3.93 | 2.91E-06 |
|  | 16580802 | 4 |  |  |  | 5.22E-07 | 4.24 | 8.33E-07 | 1.53E-06 | 4.01 | 2.33E-06 |  |  |  | 6.55E-08 | 5.01 | 5.78E-07 | 3.14E-07 | 4.44 | 1.13E-06 |
|  | 16585045 | 4 |  |  |  | 5.90E-07 | 4.22 | 9.38E-07 | 2.35E-07 | 4.55 | 8.33E-07 |  |  |  | 4.84E-08 | 5.19 | 4.05E-07 | 7.08E-07 | 4.18 | 1.59E-06 |

STL, stigma length; SYL, style length; TSSL, the sum of stigma and style length; E1, environment 1, 2014 in Nanjing; E2, environment 2, 2015 in Nanjing; E3, environment 3, 2016 in Nanjing; E4, environment 4, 2014 in Yuanyang; E5, environment 5, 2014 in Yuanyang; E6, environment 6, 2014 in Yuanyang
