# Supplementary material for: OsSYL2 AA, an allele identified by gene‐based association, increases style length in rice (Oryza sativa L.)
Source: Plant J. 2020 Oct 30;104(6):1491–503. doi: 10.1111/tpj.15013 (PMC7821000; doi:10.1111/tpj.15013)
Supplement: Supplementary file 6 — Table S5. The single‐nucleotide polymorphism information in the 16.69–16.87 Mb candidate region for style length and the sum of stigma and style length traits. [file TPJ-104-1491-s006.docx]

**Table S5**. The SNP information in 16.69-16.87Mb candidate region for style length and the sum of stigma and style length traits.

| Gene ID | MSU ID | SNP Location | Reference | Alteratiive | Region | Variation type | Associated signal in GWAS (P-value) | Gene-based association with traits |
| --- | --- | --- | --- | --- | --- | --- | --- | --- |
|  |  |  |  |  |  |  |  |  |
| Os03g0406900 | LOC_Os03g29340 | 3:16690189 | C | T | upstream |  |  |  |
| Os03g0406900 | LOC_Os03g29340 | 3:16690303 | G | A | upstream |  |  |  |
| Os03g0406900 | LOC_Os03g29340 | 3:16690335 | C | T | upstream |  |  |  |
| Os03g0406900 | LOC_Os03g29340 | 3:16690397 | C | T | upstream |  |  |  |
| Os03g0406900 | LOC_Os03g29340 | 3:16690429 | G | A | upstream |  |  |  |
| Os03g0406900 | LOC_Os03g29340 | 3:16690586 | C | T | upstream |  |  |  |
| Os03g0406900 | LOC_Os03g29340 | 3:16690628 | C | T | upstream |  |  |  |
| Os03g0406900 | LOC_Os03g29340 | 3:16690657 | C | A | upstream |  |  |  |
| Os03g0406900 | LOC_Os03g29340 | 3:16690775 | A | T | upstream |  |  |  |
| Os03g0406900 | LOC_Os03g29340 | 3:16690940 | T | C | upstream |  |  |  |
| Os03g0406900 | LOC_Os03g29340 | 3:16691264 | C | T | upstream |  |  |  |
| Os03g0406900 | LOC_Os03g29340 | 3:16691270 | T | C | upstream |  |  |  |
| Os03g0406900 | LOC_Os03g29340 | 3:16691867 | A | G | upstream |  |  |  |
| Os03g0406900 | LOC_Os03g29340 | 3:16691922 | G | T | upstream |  |  |  |
| Os03g0406900 | LOC_Os03g29340 | 3:16691963 | G | A | upstream |  |  |  |
| Os03g0406900 | LOC_Os03g29340 | 3:16691998 | G | T | upstream |  | 1.04E-06 |  |
| Os03g0406900 | LOC_Os03g29340 | 3:16692163 | C | T | upstream |  |  |  |
| Os03g0406900 | LOC_Os03g29340 | 3:16692196 | C | A | upstream |  |  |  |
| Os03g0406900 | LOC_Os03g29340 | 3:16692244 | T | G | upstream |  |  |  |
| Os03g0406900 | LOC_Os03g29340 | 3:16692351 | A | G | upstream |  |  |  |
| Os03g0406900 | LOC_Os03g29340 | 3:16692380 | A | C | upstream |  |  |  |
| Os03g0406900 | LOC_Os03g29340 | 3:16692429 | G | T | upstream |  |  |  |
| Os03g0406900 | LOC_Os03g29340 | 3:16692443 | A | G | upstream |  |  |  |
| Os03g0406900 | LOC_Os03g29340 | 3:16692487 | T | A | upstream |  |  |  |
| Os03g0406900 | LOC_Os03g29340 | 3:16692499 | T | A | upstream |  |  |  |
| Os03g0406900 | LOC_Os03g29340 | 3:16692659 | T | C | 5_prime_UTR |  |  |  |
| Os03g0406900 | LOC_Os03g29340 | 3:16692988 | A | G | 5_prime_UTR |  |  |  |
| Os03g0406900 | LOC_Os03g29340 | 3:16694097 | G | C | exonic | nonsynonymous |  |  |
| Os03g0406900 | LOC_Os03g29340 | 3:16696073 | T | G | exonic | synonymous |  |  |
| Os03g0406900 | LOC_Os03g29340 | 3:16696853 | T | C | downstream |  |  |  |
| Os03g0406900 | LOC_Os03g29340 | 3:16697063 | T | C | downstream |  |  |  |
| Os03g0406900 | LOC_Os03g29340 | 3:16697295 | C | A | downstream |  |  |  |
| Os03g0406900 | LOC_Os03g29340 | 3:16697417 | C | T | downstream |  |  |  |
| Os03g0406900 | LOC_Os03g29340 | 3:16697922 | T | A | downstream |  |  |  |
| Os03g0406900 | LOC_Os03g29340 | 3:16698018 | A | G | downstream |  |  |  |
| Os03g0406900 | LOC_Os03g29340 | 3:16698457 | C | T | downstream |  |  |  |
| Os03g0406900 | LOC_Os03g29340 | 3:16698473 | G | T | downstream |  |  |  |
| Os03g0406900 | LOC_Os03g29340 | 3:16698644 | C | T | downstream |  |  |  |
| Os03g0406900 | LOC_Os03g29340 | 3:16698667 | T | A | downstream |  |  |  |
| Os03g0406900 | LOC_Os03g29340 | 3:16698809 | C | G | downstream |  |  |  |
| Os03g0406900 | LOC_Os03g29340 | 3:16699476 | G | T | downstream |  |  |  |
| Os03g0406900 | LOC_Os03g29340 | 3:16699633 | G | T | downstream |  |  |  |
| Os03g0406900 | LOC_Os03g29340 | 3:16700094 | A | G | downstream |  |  |  |
| Os03g0406900 | LOC_Os03g29340 | 3:16700140 | C | G | downstream |  |  |  |
| Os03g0406900 | LOC_Os03g29340 | 3:16700638 | A | G | downstream |  |  |  |
| Os03g0407000 | LOC_Os03g29350 | 3:16696465 | G | A | 3_prime_UTR |  |  |  |
| Os03g0407000 | LOC_Os03g29350 | 3:16697605 | C | A | exonic | synonymous |  |  |
| Os03g0407000 | LOC_Os03g29350 | 3:16699913 | A | G | exonic | nonsynonymous |  |  |
| Os03g0407000 | LOC_Os03g29350 | 3:16700676 | A | G | exonic | synonymous |  |  |
| Os03g0407000 | LOC_Os03g29350 | 3:16700951 | G | C | exonic | nonsynonymous |  |  |
| Os03g0407000 | LOC_Os03g29350 | 3:16701692 | T | G | upstream |  |  |  |
| Os03g0407000 | LOC_Os03g29350 | 3:16701950 | G | A | upstream |  |  |  |
| Os03g0407000 | LOC_Os03g29350 | 3:16702037 | A | C | upstream |  |  |  |
| Os03g0407000 | LOC_Os03g29350 | 3:16702081 | A | G | upstream |  |  |  |
| Os03g0407000 | LOC_Os03g29350 | 3:16702084 | G | A | upstream |  |  |  |
| Os03g0407000 | LOC_Os03g29350 | 3:16702095 | C | A | upstream |  |  |  |
| Os03g0407000 | LOC_Os03g29350 | 3:16702105 | G | T | upstream |  |  |  |
| Os03g0407000 | LOC_Os03g29350 | 3:16702315 | A | C | upstream |  |  |  |
| Os03g0407000 | LOC_Os03g29350 | 3:16702543 | T | A | upstream |  |  |  |
| Os03g0407000 | LOC_Os03g29350 | 3:16703094 | G | A | upstream |  |  |  |
| Os03g0407000 | LOC_Os03g29350 | 3:16703232 | C | A | upstream |  |  |  |
| Os03g0407000 | LOC_Os03g29350 | 3:16703615 | C | T | upstream |  |  |  |
| Os03g0407000 | LOC_Os03g29350 | 3:16703703 | G | C | upstream |  |  |  |
| Os03g0407000 | LOC_Os03g29350 | 3:16703998 | C | T | upstream |  |  |  |
| Os03g0407000 | LOC_Os03g29350 | 3:16704606 | A | G | upstream |  |  |  |
| Os03g0407000 | LOC_Os03g29350 | 3:16704730 | A | G | upstream |  |  |  |
| Os03g0407000 | LOC_Os03g29350 | 3:16705165 | T | A | upstream |  |  |  |
| Os03g0407000 | LOC_Os03g29350 | 3:16705168 | A | T | upstream |  |  |  |
| Os03g0407000 | LOC_Os03g29350 | 3:16705256 | C | A | upstream |  |  |  |
| Os03g0407000 | LOC_Os03g29350 | 3:16705293 | C | T | upstream |  |  |  |
| Os03g0407000 | LOC_Os03g29350 | 3:16705394 | A | T | upstream |  |  |  |
| Os03g0407000 | LOC_Os03g29350 | 3:16705635 | G | A | upstream |  |  |  |
| Os03g0407000 | LOC_Os03g29350 | 3:16705674 | T | G | upstream |  |  |  |
| Os03g0407000 | LOC_Os03g29350 | 3:16705740 | T | C | upstream |  |  |  |
| Os03g0407000 | LOC_Os03g29350 | 3:16705800 | T | C | upstream |  |  |  |
| Os03g0407000 | LOC_Os03g29350 | 3:16705835 | A | G | upstream |  |  |  |
| Os03g0407000 | LOC_Os03g29350 | 3:16705836 | C | T | upstream |  |  |  |
| Os03g0407000 | LOC_Os03g29350 | 3:16705883 | G | A | upstream |  |  |  |
| Os03g0407000 | LOC_Os03g29350 | 3:16705901 | T | A | upstream |  |  |  |
| Os03g0407050 | LOC_Os03g29340 | 3:16692706 | C | T | upstream |  |  |  |
| Os03g0407050 | LOC_Os03g29340 | 3:16692834 | A | G | upstream |  | 1.59E-06 |  |
| Os03g0407050 | LOC_Os03g29340 | 3:16693461 | G | A | upstream |  |  |  |
| Os03g0407050 | LOC_Os03g29340 | 3:16693508 | T | G | upstream |  |  |  |
| Os03g0407050 | LOC_Os03g29340 | 3:16693675 | T | A | upstream |  |  |  |
| Os03g0407050 | LOC_Os03g29340 | 3:16693930 | A | G | upstream |  |  |  |
| Os03g0407050 | LOC_Os03g29340 | 3:16693989 | A | G | upstream |  |  |  |
| Os03g0407050 | LOC_Os03g29340 | 3:16694469 | G | A | upstream |  |  |  |
| Os03g0407050 | LOC_Os03g29340 | 3:16694528 | G | C | upstream |  |  |  |
| Os03g0407050 | LOC_Os03g29340 | 3:16694597 | T | C | upstream |  |  |  |
| Os03g0407050 | LOC_Os03g29340 | 3:16694613 | A | G | upstream |  |  |  |
| Os03g0407050 | LOC_Os03g29340 | 3:16694624 | A | C | upstream |  |  |  |
| Os03g0407050 | LOC_Os03g29340 | 3:16694694 | G | A | upstream |  |  |  |
| Os03g0407050 | LOC_Os03g29340 | 3:16694739 | T | A | upstream |  |  |  |
| Os03g0407050 | LOC_Os03g29340 | 3:16694830 | C | T | upstream |  |  |  |
| Os03g0407050 | LOC_Os03g29340 | 3:16694993 | T | A | upstream |  |  |  |
| Os03g0407050 | LOC_Os03g29340 | 3:16695507 | T | C | upstream |  |  |  |
| Os03g0407050 | LOC_Os03g29340 | 3:16695517 | T | C | upstream |  |  |  |
| Os03g0407050 | LOC_Os03g29340 | 3:16701539 | C | A | intron |  |  |  |
| Os03g0407050 | LOC_Os03g29340 | 3:16706222 | T | C | downstream |  |  |  |
| Os03g0407050 | LOC_Os03g29340 | 3:16707592 | A | G | downstream |  |  |  |
| Os03g0407050 | LOC_Os03g29340 | 3:16707808 | G | A | downstream |  |  |  |
| Os03g0407050 | LOC_Os03g29340 | 3:16707862 | A | G | downstream |  |  |  |
| Os03g0407050 | LOC_Os03g29340 | 3:16707897 | A | T | downstream |  |  |  |
| Os03g0407050 | LOC_Os03g29340 | 3:16707960 | T | C | downstream |  |  |  |
| Os03g0407050 | LOC_Os03g29340 | 3:16708049 | A | G | downstream |  |  |  |
| Os03g0407050 | LOC_Os03g29340 | 3:16708131 | C | T | downstream |  |  |  |
| Os03g0407050 | LOC_Os03g29340 | 3:16708194 | A | C | downstream |  |  |  |
| Os03g0407050 | LOC_Os03g29340 | 3:16708413 | C | T | downstream |  |  |  |
| Os03g0407050 | LOC_Os03g29340 | 3:16708465 | C | T | downstream |  |  |  |
| Os03g0407050 | LOC_Os03g29340 | 3:16708478 | G | A | downstream |  |  |  |
| Os03g0407050 | LOC_Os03g29340 | 3:16708508 | A | C | downstream |  |  |  |
| Os03g0407050 | LOC_Os03g29340 | 3:16709253 | C | T | downstream |  |  |  |
| Os03g0407050 | LOC_Os03g29340 | 3:16709274 | C | T | downstream |  |  |  |
| Os03g0407050 | LOC_Os03g29340 | 3:16709378 | A | T | downstream |  |  |  |
| Os03g0407050 | LOC_Os03g29340 | 3:16709383 | C | T | downstream |  |  |  |
| Os03g0407050 | LOC_Os03g29340 | 3:16709385 | A | T | downstream |  |  |  |
| Os03g0407050 | LOC_Os03g29340 | 3:16709430 | A | C | downstream |  |  |  |
| Os03g0407050 | LOC_Os03g29340 | 3:16709460 | C | A | downstream |  |  |  |
| Os03g0407050 | LOC_Os03g29340 | 3:16709468 | G | A | downstream |  |  |  |
| Os03g0407050 | LOC_Os03g29340 | 3:16709473 | T | A | downstream |  |  |  |
| Os03g0407050 | LOC_Os03g29340 | 3:16709532 | G | A | downstream |  |  |  |
| Os03g0407050 | LOC_Os03g29340 | 3:16709538 | T | C | downstream |  |  |  |
| Os03g0407050 | LOC_Os03g29340 | 3:16709540 | G | A | downstream |  |  |  |
| Os03g0407100 | LOC_Os03g29360 | 3:16710411 | A | T | upstream |  |  |  |
| Os03g0407100 | LOC_Os03g29360 | 3:16710471 | C | A | upstream |  |  |  |
| Os03g0407100 | LOC_Os03g29360 | 3:16710551 | C | T | upstream |  |  |  |
| Os03g0407100 | LOC_Os03g29360 | 3:16711077 | T | C | upstream |  |  |  |
| Os03g0407100 | LOC_Os03g29360 | 3:16711170 | G | A | upstream |  |  |  |
| Os03g0407100 | LOC_Os03g29360 | 3:16711171 | G | A | upstream |  |  |  |
| Os03g0407100 | LOC_Os03g29360 | 3:16711211 | G | A | upstream |  |  |  |
| Os03g0407100 | LOC_Os03g29360 | 3:16711224 | C | T | upstream |  |  |  |
| Os03g0407100 | LOC_Os03g29360 | 3:16711292 | C | T | upstream |  |  |  |
| Os03g0407100 | LOC_Os03g29360 | 3:16711383 | C | T | upstream |  |  |  |
| Os03g0407100 | LOC_Os03g29360 | 3:16711582 | T | A | upstream |  |  |  |
| Os03g0407100 | LOC_Os03g29360 | 3:16711614 | A | G | upstream |  |  |  |
| Os03g0407100 | LOC_Os03g29360 | 3:16711656 | T | C | upstream |  |  |  |
| Os03g0407100 | LOC_Os03g29360 | 3:16711688 | G | C | upstream |  |  |  |
| Os03g0407100 | LOC_Os03g29360 | 3:16711725 | C | T | upstream |  |  |  |
| Os03g0407100 | LOC_Os03g29360 | 3:16711885 | T | C | upstream |  |  |  |
| Os03g0407100 | LOC_Os03g29360 | 3:16712178 | C | A | upstream |  |  |  |
| Os03g0407100 | LOC_Os03g29360 | 3:16712202 | A | G | upstream |  |  |  |
| Os03g0407100 | LOC_Os03g29360 | 3:16712203 | C | T | upstream |  |  |  |
| Os03g0407100 | LOC_Os03g29360 | 3:16712300 | C | T | upstream |  |  |  |
| Os03g0407100 | LOC_Os03g29360 | 3:16712346 | C | T | upstream |  |  |  |
| Os03g0407100 | LOC_Os03g29360 | 3:16712470 | A | G | upstream |  |  |  |
| Os03g0407100 | LOC_Os03g29360 | 3:16712539 | C | A | upstream |  |  |  |
| Os03g0407100 | LOC_Os03g29360 | 3:16712610 | G | A | upstream |  |  |  |
| Os03g0407100 | LOC_Os03g29360 | 3:16712619 | G | A | upstream |  |  |  |
| Os03g0407100 | LOC_Os03g29360 | 3:16712650 | T | C | upstream |  |  |  |
| Os03g0407100 | LOC_Os03g29360 | 3:16712661 | C | T | upstream |  |  |  |
| Os03g0407100 | LOC_Os03g29360 | 3:16712662 | C | T | upstream |  |  |  |
| Os03g0407100 | LOC_Os03g29360 | 3:16712664 | C | T | upstream |  |  |  |
| Os03g0407100 | LOC_Os03g29360 | 3:16712761 | A | T | upstream |  |  |  |
| Os03g0407100 | LOC_Os03g29360 | 3:16712765 | C | T | upstream |  |  |  |
| Os03g0407100 | LOC_Os03g29360 | 3:16712810 | A | C | upstream |  |  |  |
| Os03g0407100 | LOC_Os03g29360 | 3:16712910 | G | C | upstream |  |  |  |
| Os03g0407100 | LOC_Os03g29360 | 3:16712982 | T | C | upstream |  |  |  |
| Os03g0407100 | LOC_Os03g29360 | 3:16713093 | C | T | upstream |  |  |  |
| Os03g0407100 | LOC_Os03g29360 | 3:16713164 | A | G | upstream |  |  |  |
| Os03g0407100 | LOC_Os03g29360 | 3:16713187 | G | A | upstream |  |  |  |
| Os03g0407100 | LOC_Os03g29360 | 3:16713193 | G | A | upstream |  |  |  |
| Os03g0407100 | LOC_Os03g29360 | 3:16713226 | A | G | upstream |  |  |  |
| Os03g0407100 | LOC_Os03g29360 | 3:16713301 | G | A | upstream |  |  |  |
| Os03g0407100 | LOC_Os03g29360 | 3:16713302 | C | A | upstream |  |  |  |
| Os03g0407100 | LOC_Os03g29360 | 3:16713327 | T | G | upstream |  |  |  |
| Os03g0407100 | LOC_Os03g29360 | 3:16713478 | C | A | upstream |  |  |  |
| Os03g0407100 | LOC_Os03g29360 | 3:16713519 | C | A | upstream |  |  |  |
| Os03g0407100 | LOC_Os03g29360 | 3:16713591 | T | A | upstream |  |  |  |
| Os03g0407100 | LOC_Os03g29360 | 3:16713630 | T | C | upstream |  |  |  |
| Os03g0407100 | LOC_Os03g29360 | 3:16713685 | A | G | upstream |  |  |  |
| Os03g0407100 | LOC_Os03g29360 | 3:16713722 | G | A | upstream |  |  |  |
| Os03g0407100 | LOC_Os03g29360 | 3:16713781 | G | A | upstream |  |  |  |
| Os03g0407100 | LOC_Os03g29360 | 3:16713855 | T | C | upstream |  |  |  |
| Os03g0407100 | LOC_Os03g29360 | 3:16713916 | G | A | upstream |  |  |  |
| Os03g0407100 | LOC_Os03g29360 | 3:16714665 | G | A | upstream |  |  |  |
| Os03g0407100 | LOC_Os03g29360 | 3:16714798 | A | G | upstream |  |  |  |
| Os03g0407100 | LOC_Os03g29360 | 3:16714912 | T | G | upstream |  |  |  |
| Os03g0407100 | LOC_Os03g29360 | 3:16714963 | G | T | upstream |  |  |  |
| Os03g0407100 | LOC_Os03g29360 | 3:16715200 | A | G | upstream |  |  |  |
| Os03g0407100 | LOC_Os03g29360 | 3:16715212 | T | C | upstream |  |  |  |
| Os03g0407100 | LOC_Os03g29360 | 3:16715980 | G | T | downstream |  |  |  |
| Os03g0407100 | LOC_Os03g29360 | 3:16716401 | C | A | downstream |  |  |  |
| Os03g0407100 | LOC_Os03g29360 | 3:16716619 | T | A | downstream |  |  |  |
| Os03g0407100 | LOC_Os03g29360 | 3:16716976 | A | T | downstream |  |  |  |
| Os03g0407100 | LOC_Os03g29360 | 3:16717176 | A | G | downstream |  |  |  |
| Os03g0407100 | LOC_Os03g29360 | 3:16717258 | A | G | downstream |  |  |  |
| Os03g0407100 | LOC_Os03g29360 | 3:16717323 | T | G | downstream |  |  |  |
| Os03g0407100 | LOC_Os03g29360 | 3:16717380 | C | A | downstream |  |  |  |
| Os03g0407100 | LOC_Os03g29360 | 3:16717401 | G | C | downstream |  |  |  |
| Os03g0407100 | LOC_Os03g29360 | 3:16717406 | A | G | downstream |  |  |  |
| Os03g0407100 | LOC_Os03g29360 | 3:16717443 | T | C | downstream |  |  |  |
| Os03g0407100 | LOC_Os03g29360 | 3:16717452 | G | A | downstream |  |  |  |
| Os03g0407100 | LOC_Os03g29360 | 3:16717611 | T | C | downstream |  |  |  |
| Os03g0407100 | LOC_Os03g29360 | 3:16717631 | A | C | downstream |  |  |  |
| Os03g0407100 | LOC_Os03g29360 | 3:16717789 | A | T | downstream |  |  |  |
| Os03g0407100 | LOC_Os03g29360 | 3:16717796 | A | T | downstream |  |  |  |
| Os03g0407100 | LOC_Os03g29360 | 3:16717862 | G | A | downstream |  |  |  |
| Os03g0407100 | LOC_Os03g29360 | 3:16717923 | G | A | downstream |  | 1.75E-06 |  |
| Os03g0407100 | LOC_Os03g29360 | 3:16717925 | T | C | downstream |  | 1.81E-06 |  |
| Os03g0407100 | LOC_Os03g29360 | 3:16718104 | C | T | downstream |  |  |  |
| Os03g0407100 | LOC_Os03g29360 | 3:16718155 | T | C | downstream |  |  |  |
| Os03g0407100 | LOC_Os03g29360 | 3:16718224 | C | A | downstream |  |  |  |
| Os03g0407100 | LOC_Os03g29360 | 3:16718368 | G | T | downstream |  |  |  |
| Os03g0407100 | LOC_Os03g29360 | 3:16718475 | A | T | downstream |  |  |  |
| Os03g0407100 | LOC_Os03g29360 | 3:16718588 | G | A | downstream |  |  |  |
| Os03g0407100 | LOC_Os03g29360 | 3:16718617 | G | A | downstream |  |  |  |
| Os03g0407100 | LOC_Os03g29360 | 3:16718625 | C | T | downstream |  |  |  |
| Os03g0407100 | LOC_Os03g29360 | 3:16718672 | A | G | downstream |  |  |  |
| Os03g0407100 | LOC_Os03g29360 | 3:16718846 | T | C | downstream |  |  |  |
| Os03g0407100 | LOC_Os03g29360 | 3:16718856 | G | A | downstream |  |  |  |
| Os03g0407100 | LOC_Os03g29360 | 3:16718865 | C | A | downstream |  |  |  |
| Os03g0407100 | LOC_Os03g29360 | 3:16718934 | C | T | downstream |  |  |  |
| Os03g0407100 | LOC_Os03g29360 | 3:16718953 | T | G | downstream |  |  |  |
| Os03g0407100 | LOC_Os03g29360 | 3:16719028 | C | A | downstream |  |  |  |
| Os03g0407100 | LOC_Os03g29360 | 3:16719032 | C | T | downstream |  | 1.78E-06 |  |
| Os03g0407100 | LOC_Os03g29360 | 3:16719056 | G | A | downstream |  |  |  |
| Os03g0407100 | LOC_Os03g29360 | 3:16719193 | G | C | downstream |  |  |  |
| Os03g0407100 | LOC_Os03g29360 | 3:16719368 | G | A | downstream |  |  |  |
| Os03g0407100 | LOC_Os03g29360 | 3:16719419 | T | A | downstream |  |  |  |
| Os03g0407100 | LOC_Os03g29360 | 3:16719451 | G | A | downstream |  |  |  |
| Os03g0407100 | LOC_Os03g29360 | 3:16719480 | A | G | downstream |  |  |  |
| Os03g0407100 | LOC_Os03g29360 | 3:16719531 | C | G | downstream |  |  |  |
| Os03g0407100 | LOC_Os03g29360 | 3:16719628 | T | C | downstream |  |  |  |
| Os03g0407100 | LOC_Os03g29360 | 3:16719646 | G | A | downstream |  |  |  |
| Os03g0407100 | LOC_Os03g29360 | 3:16719708 | C | G | downstream |  |  |  |
| Os03g0407100 | LOC_Os03g29360 | 3:16719744 | T | C | downstream |  |  |  |
| Os03g0407100 | LOC_Os03g29360 | 3:16719814 | T | C | downstream |  |  |  |
| Os03g0407100 | LOC_Os03g29360 | 3:16720103 | T | A | downstream |  |  |  |
| Os03g0407100 | LOC_Os03g29360 | 3:16720151 | C | T | downstream |  |  |  |
| Os03g0407100 | LOC_Os03g29360 | 3:16720396 | C | A | downstream |  |  |  |
| Os03g0407100 | LOC_Os03g29360 | 3:16720463 | A | T | downstream |  | 1.13E-08 |  |
| Os03g0407100 | LOC_Os03g29360 | 3:16720589 | C | T | downstream |  |  |  |
| Os03g0407400 | None | 3:16724594 | G | A | downstream |  |  |  |
| Os03g0407400 | None | 3:16724624 | A | G | downstream |  |  |  |
| Os03g0407400 | None | 3:16724690 | G | A | downstream |  |  |  |
| Os03g0407400 | None | 3:16724730 | A | G | downstream |  |  |  |
| Os03g0407400 | None | 3:16724810 | A | T | downstream |  |  |  |
| Os03g0407400 | None | 3:16724816 | C | T | downstream |  |  |  |
| Os03g0407400 | None | 3:16725157 | T | C | downstream |  | 5.33E-07 |  |
| Os03g0407400 | None | 3:16725232 | C | T | downstream |  |  |  |
| Os03g0407400 | None | 3:16725235 | G | A | downstream |  |  |  |
| Os03g0407400 | None | 3:16725263 | T | C | downstream |  |  |  |
| Os03g0407400 | None | 3:16725383 | G | A | downstream |  |  |  |
| Os03g0407400 | None | 3:16725480 | G | A | downstream |  |  |  |
| Os03g0407400 | None | 3:16725674 | A | T | downstream |  |  |  |
| Os03g0407400 | None | 3:16725700 | C | T | downstream |  |  |  |
| Os03g0407400 | None | 3:16725709 | T | C | downstream |  |  |  |
| Os03g0407400 | None | 3:16726556 | T | C | downstream |  |  |  |
| Os03g0407400 | None | 3:16726613 | C | T | downstream |  |  |  |
| Os03g0407400 | None | 3:16726643 | A | G | downstream |  |  |  |
| Os03g0407400 | None | 3:16726687 | G | A | downstream |  |  |  |
| Os03g0407400 | None | 3:16726697 | A | G | downstream |  |  |  |
| Os03g0407400 | None | 3:16726742 | C | T | downstream |  |  |  |
| Os03g0407400 | None | 3:16726766 | T | C | downstream |  |  |  |
| Os03g0407400 | None | 3:16726768 | G | A | downstream |  |  |  |
| Os03g0407400 | None | 3:16726790 | G | A | downstream |  |  |  |
| Os03g0407400 | None | 3:16726928 | A | T | downstream |  |  |  |
| Os03g0407400 | None | 3:16726980 | T | A | downstream |  |  |  |
| Os03g0407400 | None | 3:16727052 | G | A | downstream |  |  |  |
| Os03g0407400 | None | 3:16727112 | A | C | downstream |  |  |  |
| Os03g0407400 | None | 3:16727284 | G | T | downstream |  |  |  |
| Os03g0407400 | None | 3:16727307 | C | T | downstream |  |  |  |
| Os03g0407400 | None | 3:16727310 | G | A | downstream |  |  |  |
| Os03g0407400 | None | 3:16727324 | C | T | downstream |  |  |  |
| Os03g0407400 | None | 3:16727339 | G | A | downstream |  |  |  |
| Os03g0407400 | None | 3:16727355 | C | T | downstream |  |  |  |
| Os03g0407400 | None | 3:16727363 | C | T | downstream |  |  |  |
| Os03g0407400 | None | 3:16727471 | T | C | downstream |  |  |  |
| Os03g0407400 | None | 3:16728210 | C | T | downstream |  |  |  |
| Os03g0407400 | None | 3:16728216 | A | G | downstream |  |  |  |
| Os03g0407400 | None | 3:16728241 | C | T | downstream |  |  |  |
| Os03g0407400 | None | 3:16728246 | G | A | downstream |  |  |  |
| Os03g0407400 | None | 3:16728293 | C | T | downstream |  |  |  |
| Os03g0407400 | None | 3:16728308 | T | C | downstream |  |  |  |
| Os03g0407400 | None | 3:16728347 | A | T | downstream |  |  |  |
| Os03g0407400 | None | 3:16728414 | C | T | downstream |  |  |  |
| Os03g0407400 | None | 3:16728422 | A | G | downstream |  |  |  |
| Os03g0407400 | None | 3:16728550 | C | T | downstream |  |  |  |
| Os03g0407400 | None | 3:16728561 | T | C | downstream |  |  |  |
| Os03g0407400 | None | 3:16728674 | C | A | downstream |  |  |  |
| Os03g0407400 | None | 3:16728680 | C | T | downstream |  |  |  |
| Os03g0407400 | None | 3:16728820 | T | A | downstream |  |  |  |
| Os03g0407400 | None | 3:16728832 | G | A | downstream |  |  |  |
| Os03g0407400 | None | 3:16728884 | G | A | downstream |  |  |  |
| Os03g0407400 | None | 3:16728961 | A | G | downstream |  |  |  |
| Os03g0407400 | None | 3:16728987 | C | T | downstream |  |  |  |
| Os03g0407400 | None | 3:16729009 | A | T | downstream |  |  |  |
| Os03g0407400 | None | 3:16729083 | A | G | downstream |  |  |  |
| Os03g0407400 | None | 3:16729220 | C | G | downstream |  |  |  |
| Os03g0407400 | None | 3:16729261 | T | C | downstream |  |  |  |
| Os03g0407400 | None | 3:16729263 | C | T | downstream |  |  |  |
| Os03g0407400 | None | 3:16729325 | T | C | downstream |  |  |  |
| Os03g0407400 | None | 3:16729492 | A | G | downstream |  |  |  |
| Os03g0407400 | None | 3:16730184 | C | G | intron |  |  |  |
| Os03g0407400 | None | 3:16730185 | A | G | intron |  |  |  |
| Os03g0407400 | None | 3:16730370 | A | G | intron |  |  |  |
| Os03g0407400 | None | 3:16730450 | A | G | intron |  |  |  |
| Os03g0407400 | None | 3:16730746 | A | G | intron |  |  |  |
| Os03g0407400 | None | 3:16730953 | C | T | intron |  |  |  |
| Os03g0407400 | None | 3:16731043 | T | C | intron |  |  |  |
| Os03g0407400 | None | 3:16731092 | G | A | intron |  |  |  |
| Os03g0407400 | None | 3:16731155 | G | A | intron |  |  |  |
| Os03g0407400 | None | 3:16731162 | G | A | intron |  |  |  |
| Os03g0407400 | None | 3:16731202 | G | A | intron |  |  |  |
| Os03g0407400 | None | 3:16731232 | G | A | intron |  |  |  |
| Os03g0407400 | None | 3:16731270 | T | C | intron |  |  |  |
| Os03g0407400 | None | 3:16731513 | T | C | intron |  |  |  |
| Os03g0407400 | None | 3:16732192 | A | T | intron |  |  |  |
| Os03g0407400 | None | 3:16732368 | T | C | intron |  |  |  |
| Os03g0407400 | None | 3:16732377 | T | C | intron |  |  |  |
| Os03g0407400 | None | 3:16732415 | G | T | intron |  |  |  |
| Os03g0407400 | None | 3:16732523 | T | C | intron |  |  |  |
| Os03g0407400 | None | 3:16732528 | T | A | intron |  |  |  |
| Os03g0407400 | None | 3:16732760 | G | T | intron |  |  |  |
| Os03g0407400 | None | 3:16732765 | A | G | intron |  | 1.64E-06 |  |
| Os03g0407400 | None | 3:16732967 | T | G | intron |  |  |  |
| Os03g0407400 | None | 3:16732973 | A | G | intron |  |  |  |
| Os03g0407400 | None | 3:16733111 | T | C | intron |  |  |  |
| Os03g0407400 | None | 3:16733441 | G | T | stop_gained |  | 7.51E-08 | 2.20E-16 |
| Os03g0407400 | None | 3:16733773 | T | A | intron |  |  |  |
| Os03g0407400 | None | 3:16733833 | G | C | intron |  |  |  |
| Os03g0407400 | None | 3:16733868 | C | T | intron |  |  |  |
| Os03g0407400 | None | 3:16733872 | T | G | intron |  |  |  |
| Os03g0407400 | None | 3:16734064 | T | C | intron |  |  |  |
| Os03g0407400 | None | 3:16734121 | A | C | intron |  |  |  |
| Os03g0407400 | None | 3:16734333 | C | A | intron |  |  |  |
| Os03g0407400 | None | 3:16734524 | A | G | intron |  |  |  |
| Os03g0407400 | None | 3:16734618 | T | C | intron |  |  |  |
| Os03g0407400 | None | 3:16734646 | G | A | intron |  |  |  |
| Os03g0407400 | None | 3:16734715 | T | C | intron |  |  |  |
| Os03g0407400 | None | 3:16734791 | A | G | intron |  |  |  |
| Os03g0407400 | None | 3:16734889 | A | C | intron |  |  |  |
| Os03g0407400 | None | 3:16735110 | G | T | upstream |  |  |  |
| Os03g0407400 | None | 3:16735437 | C | G | upstream |  |  |  |
| Os03g0407400 | None | 3:16735605 | A | C | upstream |  |  |  |
| Os03g0407400 | None | 3:16735690 | G | A | upstream |  |  |  |
| Os03g0407400 | None | 3:16735719 | T | G | upstream |  |  |  |
| Os03g0407400 | None | 3:16735756 | G | A | upstream |  |  |  |
| Os03g0407400 | None | 3:16735870 | T | C | upstream |  |  |  |
| Os03g0407400 | None | 3:16735872 | T | A | upstream |  |  |  |
| Os03g0407400 | None | 3:16735902 | G | A | upstream |  |  |  |
| Os03g0407400 | None | 3:16735925 | G | C | upstream |  |  |  |
| Os03g0407400 | None | 3:16735998 | T | C | upstream |  |  |  |
| Os03g0407400 | None | 3:16736049 | T | C | upstream |  |  |  |
| Os03g0407400 | None | 3:16736089 | A | T | upstream |  |  |  |
| Os03g0407400 | None | 3:16736782 | C | A | upstream |  |  |  |
| Os03g0407400 | None | 3:16736817 | G | A | upstream |  |  |  |
| Os03g0407400 | None | 3:16736820 | G | A | upstream |  |  |  |
| Os03g0407400 | None | 3:16736874 | A | T | upstream |  |  |  |
| Os03g0407400 | None | 3:16736876 | A | G | upstream |  |  |  |
| Os03g0407400 | None | 3:16737022 | C | A | upstream |  |  |  |
| Os03g0407400 | None | 3:16737085 | G | A | upstream |  |  |  |
| Os03g0407400 | None | 3:16737094 | T | G | upstream |  |  |  |
| Os03g0407400 | None | 3:16737307 | A | G | upstream |  |  |  |
| Os03g0407400 | None | 3:16737323 | C | T | upstream |  |  |  |
| Os03g0407400 | None | 3:16737397 | A | G | upstream |  |  |  |
| Os03g0407400 | None | 3:16737591 | C | T | upstream |  |  |  |
| Os03g0407400 | None | 3:16737823 | C | T | upstream |  |  |  |
| Os03g0407900 | LOC_Os03g29410 | 3:16749354 | G | A | 3_prime_UTR |  |  |  |
| Os03g0407900 | LOC_Os03g29410 | 3:16749454 | C | T | 3_prime_UTR |  |  |  |
| Os03g0407900 | LOC_Os03g29410 | 3:16749510 | C | G | exonic | nonsynonymous |  |  |
| Os03g0407900 | LOC_Os03g29410 | 3:16749678 | C | T | exonic | nonsynonymous |  |  |
| Os03g0407900 | LOC_Os03g29410 | 3:16749801 | T | C | exonic | nonsynonymous |  |  |
| Os03g0407900 | LOC_Os03g29410 | 3:16749802 | T | G | exonic | nonsynonymous |  |  |
| Os03g0407900 | LOC_Os03g29410 | 3:16749840 | A | G | exonic | nonsynonymous |  |  |
| Os03g0407900 | LOC_Os03g29410 | 3:16749884 | C | T | exonic | synonymous |  |  |
| Os03g0407900 | LOC_Os03g29410 | 3:16749956 | A | G | exonic | synonymous |  |  |
| Os03g0407900 | LOC_Os03g29410 | 3:16750033 | A | G | intron |  |  |  |
| Os03g0407900 | LOC_Os03g29410 | 3:16750223 | C | T | exonic | nonsynonymous |  |  |
| Os03g0407900 | LOC_Os03g29410 | 3:16750312 | C | A | intron |  |  |  |
| Os03g0407900 | LOC_Os03g29410 | 3:16750313 | C | A | intron |  |  |  |
| Os03g0407900 | LOC_Os03g29410 | 3:16750348 | G | A | intron |  |  |  |
| Os03g0407900 | LOC_Os03g29410 | 3:16751279 | C | T | intron |  |  |  |
| Os03g0407900 | LOC_Os03g29410 | 3:16751315 | T | C | intron |  |  |  |
| Os03g0407900 | LOC_Os03g29410 | 3:16751851 | T | C | intron |  |  |  |
| Os03g0407900 | LOC_Os03g29410 | 3:16751964 | T | C | intron |  |  |  |
| Os03g0407900 | LOC_Os03g29410 | 3:16752072 | T | A | intron |  |  |  |
| Os03g0407900 | LOC_Os03g29410 | 3:16753120 | A | G | intron |  |  |  |
| Os03g0407900 | LOC_Os03g29410 | 3:16753132 | C | T | intron |  |  |  |
| Os03g0407900 | LOC_Os03g29410 | 3:16753472 | A | C | intron |  |  |  |
| Os03g0407900 | LOC_Os03g29410 | 3:16754026 | G | A | exonic | synonymous |  |  |
| Os03g0407900 | LOC_Os03g29410 | 3:16754120 | C | T | 5_prime_UTR |  |  |  |
| Os03g0407900 | LOC_Os03g29410 | 3:16754148 | C | A | 5_prime_UTR |  |  |  |
| Os03g0407900 | LOC_Os03g29410 | 3:16754246 | T | G | upstream |  |  |  |
| Os03g0407900 | LOC_Os03g29410 | 3:16754424 | T | C | upstream |  |  |  |
| Os03g0407900 | LOC_Os03g29410 | 3:16754449 | G | C | upstream |  |  |  |
| Os03g0407900 | LOC_Os03g29410 | 3:16754512 | T | C | upstream |  |  |  |
| Os03g0407900 | LOC_Os03g29410 | 3:16754731 | C | T | upstream |  |  |  |
| Os03g0407900 | LOC_Os03g29410 | 3:16755198 | T | C | upstream |  |  |  |
| Os03g0407900 | LOC_Os03g29410 | 3:16756624 | C | T | upstream |  |  |  |
| Os03g0407900 | LOC_Os03g29410 | 3:16756727 | A | C | upstream |  |  |  |
| Os03g0407900 | LOC_Os03g29410 | 3:16756838 | G | A | upstream |  |  |  |
| Os03g0407900 | LOC_Os03g29410 | 3:16756921 | G | A | upstream |  |  |  |
| Os03g0407900 | LOC_Os03g29410 | 3:16756977 | C | T | upstream |  |  |  |
| Os03g0407900 | LOC_Os03g29410 | 3:16756983 | G | A | upstream |  |  |  |
| Os03g0407900 | LOC_Os03g29410 | 3:16757066 | C | T | upstream |  |  |  |
| Os03g0407900 | LOC_Os03g29410 | 3:16757079 | C | T | upstream |  |  |  |
| Os03g0407900 | LOC_Os03g29410 | 3:16757107 | C | A | upstream |  |  |  |
| Os03g0407900 | LOC_Os03g29410 | 3:16757207 | A | C | upstream |  |  |  |
| Os03g0407900 | LOC_Os03g29410 | 3:16757231 | C | T | upstream |  |  |  |
| Os03g0407900 | LOC_Os03g29410 | 3:16757261 | G | A | upstream |  |  |  |
| Os03g0407900 | LOC_Os03g29410 | 3:16757297 | G | A | upstream |  |  |  |
| Os03g0407900 | LOC_Os03g29410 | 3:16757322 | T | C | upstream |  |  |  |
| Os03g0407900 | LOC_Os03g29410 | 3:16757542 | T | C | upstream |  |  |  |
| Os03g0407900 | LOC_Os03g29410 | 3:16757563 | T | C | upstream |  |  |  |
| Os03g0407900 | LOC_Os03g29410 | 3:16757565 | T | A | upstream |  |  |  |
| Os03g0407900 | LOC_Os03g29410 | 3:16757595 | G | A | upstream |  |  |  |
| Os03g0407900 | LOC_Os03g29410 | 3:16757648 | T | C | upstream |  |  |  |
| Os03g0407900 | LOC_Os03g29410 | 3:16757675 | C | T | upstream |  |  |  |
| Os03g0407900 | LOC_Os03g29410 | 3:16757720 | G | A | upstream |  |  |  |
| Os03g0407900 | LOC_Os03g29410 | 3:16757804 | T | A | upstream |  |  |  |
| Os03g0407900 | LOC_Os03g29410 | 3:16757893 | C | T | upstream |  |  |  |
| Os03g0407900 | LOC_Os03g29410 | 3:16757898 | G | T | upstream |  |  |  |
| Os03g0407900 | LOC_Os03g29410 | 3:16758256 | A | G | upstream |  |  |  |
| Os03g0407900 | LOC_Os03g29410 | 3:16758294 | G | T | upstream |  |  |  |
| Os03g0407900 | LOC_Os03g29410 | 3:16758349 | A | T | upstream |  |  |  |
| Os03g0407900 | LOC_Os03g29410 | 3:16758402 | T | C | upstream |  |  |  |
| Os03g0407900 | LOC_Os03g29410 | 3:16758450 | G | C | upstream |  |  |  |
| Os03g0407900 | LOC_Os03g29410 | 3:16758459 | G | A | upstream |  |  |  |
| Os03g0407900 | LOC_Os03g29410 | 3:16758780 | A | C | upstream |  |  |  |
| Os03g0407900 | LOC_Os03g29410 | 3:16758787 | C | A | upstream |  |  |  |
| Os03g0407900 | LOC_Os03g29410 | 3:16758967 | A | C | upstream |  |  |  |
| Os03g0408101 | None | 3:16744754 | T | C | upstream |  |  |  |
| Os03g0408101 | None | 3:16744798 | G | T | upstream |  |  |  |
| Os03g0408101 | None | 3:16744944 | G | T | upstream |  |  |  |
| Os03g0408101 | None | 3:16745038 | T | C | upstream |  |  |  |
| Os03g0408101 | None | 3:16745115 | G | T | upstream |  |  |  |
| Os03g0408101 | None | 3:16746064 | G | A | upstream |  |  |  |
| Os03g0408101 | None | 3:16746075 | A | G | upstream |  |  |  |
| Os03g0408101 | None | 3:16746080 | G | T | upstream |  |  |  |
| Os03g0408101 | None | 3:16746172 | G | A | upstream |  |  |  |
| Os03g0408101 | None | 3:16746349 | T | C | upstream |  |  |  |
| Os03g0408101 | None | 3:16746417 | A | T | upstream |  |  |  |
| Os03g0408101 | None | 3:16746791 | A | G | upstream |  |  |  |
| Os03g0408101 | None | 3:16746802 | T | C | upstream |  |  |  |
| Os03g0408101 | None | 3:16747053 | C | T | upstream |  |  |  |
| Os03g0408101 | None | 3:16747062 | C | T | upstream |  |  |  |
| Os03g0408101 | None | 3:16747072 | A | G | upstream |  |  |  |
| Os03g0408101 | None | 3:16747120 | C | G | upstream |  |  |  |
| Os03g0408101 | None | 3:16747529 | C | T | upstream |  |  |  |
| Os03g0408101 | None | 3:16747580 | A | G | upstream |  |  |  |
| Os03g0408101 | None | 3:16747592 | A | G | upstream |  |  |  |
| Os03g0408101 | None | 3:16747632 | T | C | upstream |  |  |  |
| Os03g0408101 | None | 3:16747823 | A | G | upstream |  |  |  |
| Os03g0408101 | None | 3:16747868 | C | T | upstream |  |  |  |
| Os03g0408101 | None | 3:16748135 | T | C | upstream |  |  |  |
| Os03g0408101 | None | 3:16748168 | G | T | upstream |  |  |  |
| Os03g0408101 | None | 3:16748185 | C | T | upstream |  |  |  |
| Os03g0408101 | None | 3:16748214 | C | T | upstream |  |  |  |
| Os03g0408101 | None | 3:16748245 | A | G | upstream |  |  |  |
| Os03g0408101 | None | 3:16748266 | C | T | upstream |  |  |  |
| Os03g0408101 | None | 3:16748288 | C | A | upstream |  |  |  |
| Os03g0408101 | None | 3:16748344 | T | C | upstream |  |  |  |
| Os03g0408101 | None | 3:16748484 | G | A | upstream |  |  |  |
| Os03g0408101 | None | 3:16748598 | C | T | upstream |  |  |  |
| Os03g0408101 | None | 3:16748649 | G | A | upstream |  |  |  |
| Os03g0408101 | None | 3:16748782 | G | A | upstream |  |  |  |
| Os03g0408101 | None | 3:16748820 | T | C | upstream |  |  |  |
| Os03g0408101 | None | 3:16748824 | G | A | upstream |  |  |  |
| Os03g0408101 | None | 3:16748886 | C | T | upstream |  |  |  |
| Os03g0408101 | None | 3:16748993 | A | G | upstream |  |  |  |
| Os03g0408101 | None | 3:16749068 | T | C | upstream |  |  |  |
| Os03g0408101 | None | 3:16749170 | G | T | upstream |  |  |  |
| Os03g0408101 | None | 3:16749258 | A | G | upstream |  |  |  |
| Os03g0408101 | None | 3:16749296 | G | A | upstream |  |  |  |
| Os03g0408101 | None | 3:16750064 | T | C | 5_prime_UTR |  |  |  |
| Os03g0408101 | None | 3:16750902 | T | G | 5_prime_UTR |  |  |  |
| Os03g0408101 | None | 3:16753383 | A | G | exonic | nonsynonymous |  |  |
| Os03g0408300 | LOC_Os03g29460 | 3:16782194 | A | G | upstream |  |  |  |
| Os03g0408300 | LOC_Os03g29460 | 3:16782207 | G | A | upstream |  |  |  |
| Os03g0408300 | LOC_Os03g29460 | 3:16782310 | T | G | upstream |  |  |  |
| Os03g0408300 | LOC_Os03g29460 | 3:16782409 | C | T | upstream |  |  |  |
| Os03g0408300 | LOC_Os03g29460 | 3:16782413 | T | C | upstream |  |  |  |
| Os03g0408300 | LOC_Os03g29460 | 3:16782429 | T | G | upstream |  |  |  |
| Os03g0408300 | LOC_Os03g29460 | 3:16782456 | G | A | upstream |  |  |  |
| Os03g0408300 | LOC_Os03g29460 | 3:16782774 | G | A | upstream |  |  |  |
| Os03g0408300 | LOC_Os03g29460 | 3:16782785 | A | C | upstream |  |  |  |
| Os03g0408300 | LOC_Os03g29460 | 3:16783112 | G | A | upstream |  |  |  |
| Os03g0408300 | LOC_Os03g29460 | 3:16783315 | A | T | upstream |  |  |  |
| Os03g0408300 | LOC_Os03g29460 | 3:16783340 | A | G | upstream |  |  |  |
| Os03g0408300 | LOC_Os03g29460 | 3:16783416 | C | T | upstream |  |  |  |
| Os03g0408300 | LOC_Os03g29460 | 3:16783418 | A | G | upstream |  |  |  |
| Os03g0408300 | LOC_Os03g29460 | 3:16783495 | A | G | upstream |  |  |  |
| Os03g0408300 | LOC_Os03g29460 | 3:16783496 | T | C | upstream |  |  |  |
| Os03g0408300 | LOC_Os03g29460 | 3:16783679 | T | C | upstream |  |  |  |
| Os03g0408300 | LOC_Os03g29460 | 3:16783772 | A | G | upstream |  |  |  |
| Os03g0408300 | LOC_Os03g29460 | 3:16783783 | C | T | upstream |  |  |  |
| Os03g0408300 | LOC_Os03g29460 | 3:16783853 | T | C | upstream |  |  |  |
| Os03g0408300 | LOC_Os03g29460 | 3:16783857 | A | G | upstream |  |  |  |
| Os03g0408300 | LOC_Os03g29460 | 3:16783968 | G | A | upstream |  |  |  |
| Os03g0408300 | LOC_Os03g29460 | 3:16783977 | G | A | upstream |  |  |  |
| Os03g0408300 | LOC_Os03g29460 | 3:16784005 | C | T | upstream |  |  |  |
| Os03g0408300 | LOC_Os03g29460 | 3:16784259 | A | T | upstream |  |  |  |
| Os03g0408300 | LOC_Os03g29460 | 3:16784292 | A | G | upstream |  |  |  |
| Os03g0408300 | LOC_Os03g29460 | 3:16784433 | G | C | upstream |  |  |  |
| Os03g0408300 | LOC_Os03g29460 | 3:16786030 | A | C | upstream |  |  |  |
| Os03g0408300 | LOC_Os03g29460 | 3:16786099 | C | G | upstream |  |  |  |
| Os03g0408300 | LOC_Os03g29460 | 3:16786501 | A | T | upstream |  |  |  |
| Os03g0408300 | LOC_Os03g29460 | 3:16786700 | T | C | upstream |  |  |  |
| Os03g0408300 | LOC_Os03g29460 | 3:16786702 | A | C | upstream |  |  |  |
| Os03g0408300 | LOC_Os03g29460 | 3:16786960 | C | T | upstream |  |  |  |
| Os03g0408300 | LOC_Os03g29460 | 3:16786961 | C | T | upstream |  |  |  |
| Os03g0408300 | LOC_Os03g29460 | 3:16787790 | T | G | 3_prime_UTR |  |  |  |
| Os03g0408300 | LOC_Os03g29460 | 3:16788313 | G | A | downstream |  |  |  |
| Os03g0408300 | LOC_Os03g29460 | 3:16788584 | C | T | downstream |  |  |  |
| Os03g0408300 | LOC_Os03g29460 | 3:16788588 | C | T | downstream |  |  |  |
| Os03g0408300 | LOC_Os03g29460 | 3:16788735 | A | T | downstream |  |  |  |
| Os03g0408300 | LOC_Os03g29460 | 3:16789014 | G | C | downstream |  |  |  |
| Os03g0408300 | LOC_Os03g29460 | 3:16789092 | T | C | downstream |  |  |  |
| Os03g0408300 | LOC_Os03g29460 | 3:16789189 | T | C | downstream |  |  |  |
| Os03g0408401 | None | 3:16788124 | A | T | upstream |  |  |  |
| Os03g0408401 | None | 3:16788147 | A | T | upstream |  |  |  |
| Os03g0408401 | None | 3:16788201 | A | T | upstream |  |  |  |
| Os03g0408500 | LOC_Os03g29470 | 3:16789370 | G | A | upstream |  |  |  |
| Os03g0408500 | LOC_Os03g29470 | 3:16789793 | T | G | upstream |  |  |  |
| Os03g0408500 | LOC_Os03g29470 | 3:16789845 | G | T | upstream |  |  |  |
| Os03g0408500 | LOC_Os03g29470 | 3:16789932 | G | A | upstream |  |  |  |
| Os03g0408500 | LOC_Os03g29470 | 3:16789935 | C | T | upstream |  |  |  |
| Os03g0408500 | LOC_Os03g29470 | 3:16790404 | C | A | upstream |  |  |  |
| Os03g0408500 | LOC_Os03g29470 | 3:16790408 | C | G | upstream |  |  |  |
| Os03g0408500 | LOC_Os03g29470 | 3:16790430 | A | C | upstream |  |  |  |
| Os03g0408500 | LOC_Os03g29470 | 3:16790433 | G | T | upstream |  |  |  |
| Os03g0408500 | LOC_Os03g29470 | 3:16790514 | A | G | upstream |  |  |  |
| Os03g0408500 | LOC_Os03g29470 | 3:16790564 | G | T | upstream |  |  |  |
| Os03g0408500 | LOC_Os03g29470 | 3:16790702 | A | C | upstream |  |  |  |
| Os03g0408500 | LOC_Os03g29470 | 3:16791091 | A | G | upstream |  |  |  |
| Os03g0408500 | LOC_Os03g29470 | 3:16791407 | A | C | upstream |  |  |  |
| Os03g0408500 | LOC_Os03g29470 | 3:16791470 | A | T | upstream |  |  |  |
| Os03g0408500 | LOC_Os03g29470 | 3:16791508 | T | A | upstream |  |  |  |
| Os03g0408500 | LOC_Os03g29470 | 3:16791587 | C | T | upstream |  |  |  |
| Os03g0408500 | LOC_Os03g29470 | 3:16791603 | C | G | upstream |  |  |  |
| Os03g0408500 | LOC_Os03g29470 | 3:16791667 | G | A | upstream |  |  |  |
| Os03g0408500 | LOC_Os03g29470 | 3:16791759 | G | C | upstream |  |  |  |
| Os03g0408500 | LOC_Os03g29470 | 3:16791839 | C | T | upstream |  |  |  |
| Os03g0408500 | LOC_Os03g29470 | 3:16791947 | A | G | upstream |  |  |  |
| Os03g0408500 | LOC_Os03g29470 | 3:16792030 | C | G | upstream |  |  |  |
| Os03g0408500 | LOC_Os03g29470 | 3:16792066 | G | A | upstream |  |  |  |
| Os03g0408500 | LOC_Os03g29470 | 3:16792084 | C | T | upstream |  |  |  |
| Os03g0408500 | LOC_Os03g29470 | 3:16792116 | T | C | upstream |  |  |  |
| Os03g0408500 | LOC_Os03g29470 | 3:16792168 | G | T | upstream |  |  |  |
| Os03g0408500 | LOC_Os03g29470 | 3:16792244 | A | G | upstream |  |  |  |
| Os03g0408500 | LOC_Os03g29470 | 3:16792318 | T | C | upstream |  |  |  |
| Os03g0408500 | LOC_Os03g29470 | 3:16792408 | T | C | upstream |  |  |  |
| Os03g0408500 | LOC_Os03g29470 | 3:16792436 | G | A | upstream |  |  |  |
| Os03g0408500 | LOC_Os03g29470 | 3:16792548 | A | G | upstream |  |  |  |
| Os03g0408500 | LOC_Os03g29470 | 3:16792557 | G | A | upstream |  |  |  |
| Os03g0408500 | LOC_Os03g29470 | 3:16792639 | C | T | upstream |  |  |  |
| Os03g0408500 | LOC_Os03g29470 | 3:16792667 | G | A | upstream |  |  |  |
| Os03g0408500 | LOC_Os03g29470 | 3:16792734 | G | A | upstream |  |  |  |
| Os03g0408500 | LOC_Os03g29470 | 3:16792772 | G | A | upstream |  |  |  |
| Os03g0408500 | LOC_Os03g29470 | 3:16792969 | A | G | upstream |  |  |  |
| Os03g0408500 | LOC_Os03g29470 | 3:16793083 | T | C | upstream |  |  |  |
| Os03g0408500 | LOC_Os03g29470 | 3:16793290 | C | A | upstream |  |  |  |
| Os03g0408500 | LOC_Os03g29470 | 3:16793456 | C | A | upstream |  |  |  |
| Os03g0408500 | LOC_Os03g29470 | 3:16793499 | C | G | upstream |  |  |  |
| Os03g0408500 | LOC_Os03g29470 | 3:16793519 | A | G | upstream |  |  |  |
| Os03g0408500 | LOC_Os03g29470 | 3:16793546 | T | G | upstream |  |  |  |
| Os03g0408500 | LOC_Os03g29470 | 3:16793679 | T | C | upstream |  |  |  |
| Os03g0408500 | LOC_Os03g29470 | 3:16793721 | G | A | upstream |  |  |  |
| Os03g0408500 | LOC_Os03g29470 | 3:16793994 | G | A | upstream |  |  |  |
| Os03g0408500 | LOC_Os03g29470 | 3:16795529 | T | A | intron |  |  |  |
| Os03g0408500 | LOC_Os03g29470 | 3:16795575 | G | C | intron |  |  |  |
| Os03g0408500 | LOC_Os03g29470 | 3:16795708 | G | A | intron |  |  |  |
| Os03g0408500 | LOC_Os03g29470 | 3:16796158 | A | T | intron |  |  |  |
| Os03g0408500 | LOC_Os03g29470 | 3:16796250 | A | T | intron |  |  |  |
| Os03g0408500 | LOC_Os03g29470 | 3:16796356 | T | G | intron |  |  |  |
| Os03g0408500 | LOC_Os03g29470 | 3:16796387 | T | C | intron |  |  |  |
| Os03g0408500 | LOC_Os03g29470 | 3:16796828 | C | T | intron |  |  |  |
| Os03g0408500 | LOC_Os03g29470 | 3:16797160 | C | T | intron |  |  |  |
| Os03g0408500 | LOC_Os03g29470 | 3:16797654 | C | T | intron |  |  |  |
| Os03g0408500 | LOC_Os03g29470 | 3:16797922 | T | C | intron |  |  |  |
| Os03g0408500 | LOC_Os03g29470 | 3:16798262 | A | T | intron |  |  |  |
| Os03g0408500 | LOC_Os03g29470 | 3:16798320 | G | A | intron |  |  |  |
| Os03g0408500 | LOC_Os03g29470 | 3:16798432 | T | G | intron |  |  |  |
| Os03g0408500 | LOC_Os03g29470 | 3:16799045 | T | C | 3_prime_UTR |  |  |  |
| Os03g0408500 | LOC_Os03g29470 | 3:16799341 | G | A | 3_prime_UTR |  |  |  |
| Os03g0408500 | LOC_Os03g29470 | 3:16799368 | C | T | 3_prime_UTR |  |  |  |
| Os03g0408500 | LOC_Os03g29470 | 3:16800217 | G | A | downstream |  |  |  |
| Os03g0408500 | LOC_Os03g29470 | 3:16800371 | C | T | downstream |  |  |  |
| Os03g0408500 | LOC_Os03g29470 | 3:16800435 | T | C | downstream |  |  |  |
| Os03g0408500 | LOC_Os03g29470 | 3:16800443 | G | A | downstream |  |  |  |
| Os03g0408500 | LOC_Os03g29470 | 3:16800490 | T | C | downstream |  |  |  |
| Os03g0408500 | LOC_Os03g29470 | 3:16800568 | C | T | downstream |  |  |  |
| Os03g0408500 | LOC_Os03g29470 | 3:16800575 | C | A | downstream |  |  |  |
| Os03g0408500 | LOC_Os03g29470 | 3:16800811 | G | C | downstream |  |  |  |
| Os03g0408500 | LOC_Os03g29470 | 3:16800913 | T | C | downstream |  |  |  |
| Os03g0408500 | LOC_Os03g29470 | 3:16800922 | C | T | downstream |  |  |  |
| Os03g0408500 | LOC_Os03g29470 | 3:16800936 | C | T | downstream |  |  |  |
| Os03g0408500 | LOC_Os03g29470 | 3:16801124 | T | C | downstream |  |  |  |
| Os03g0408500 | LOC_Os03g29470 | 3:16801523 | A | G | downstream |  |  |  |
| Os03g0408500 | LOC_Os03g29470 | 3:16801528 | T | C | downstream |  |  |  |
| Os03g0408500 | LOC_Os03g29470 | 3:16801613 | A | C | downstream |  |  |  |
| Os03g0408500 | LOC_Os03g29470 | 3:16801624 | C | A | downstream |  |  |  |
| Os03g0408500 | LOC_Os03g29470 | 3:16802190 | G | A | downstream |  |  |  |
| Os03g0408500 | LOC_Os03g29470 | 3:16802210 | C | T | downstream |  |  |  |
| Os03g0408500 | LOC_Os03g29470 | 3:16802414 | G | A | downstream |  |  |  |
| Os03g0408500 | LOC_Os03g29470 | 3:16802648 | G | T | downstream |  |  |  |
| Os03g0408500 | LOC_Os03g29470 | 3:16802794 | T | C | downstream |  |  |  |
| Os03g0408500 | LOC_Os03g29470 | 3:16803029 | G | A | downstream |  |  |  |
| Os03g0408500 | LOC_Os03g29470 | 3:16803135 | T | C | downstream |  |  |  |
| Os03g0408500 | LOC_Os03g29470 | 3:16803217 | C | T | downstream |  |  |  |
| Os03g0408500 | LOC_Os03g29470 | 3:16803297 | C | T | downstream |  |  |  |
| Os03g0408500 | LOC_Os03g29470 | 3:16803597 | G | C | downstream |  |  |  |
| Os03g0408500 | LOC_Os03g29470 | 3:16803815 | A | G | downstream |  |  |  |
| Os03g0408500 | LOC_Os03g29470 | 3:16803818 | A | T | downstream |  |  |  |
| Os03g0408500 | LOC_Os03g29470 | 3:16803825 | G | A | downstream |  |  |  |
| Os03g0408500 | LOC_Os03g29470 | 3:16803953 | C | T | downstream |  |  |  |
| Os03g0408600 | LOC_Os03g29480 | 3:16805854 | C | G | exonic | nonsynonymous |  |  |
| Os03g0408600 | LOC_Os03g29480 | 3:16805935 | C | A | upstream |  |  |  |
| Os03g0408600 | LOC_Os03g29480 | 3:16806199 | C | T | upstream |  |  |  |
| Os03g0408600 | LOC_Os03g29480 | 3:16806404 | T | C | upstream |  |  |  |
| Os03g0408600 | LOC_Os03g29480 | 3:16806529 | G | C | upstream |  |  |  |
| Os03g0408600 | LOC_Os03g29480 | 3:16806647 | G | C | upstream |  |  |  |
| Os03g0408600 | LOC_Os03g29480 | 3:16806747 | T | C | upstream |  |  |  |
| Os03g0408600 | LOC_Os03g29480 | 3:16806835 | T | C | upstream |  |  |  |
| Os03g0408600 | LOC_Os03g29480 | 3:16806891 | G | A | upstream |  |  |  |
| Os03g0408600 | LOC_Os03g29480 | 3:16806894 | C | T | upstream |  |  |  |
| Os03g0408600 | LOC_Os03g29480 | 3:16806980 | C | T | upstream |  |  |  |
| Os03g0408600 | LOC_Os03g29480 | 3:16807064 | A | T | upstream |  |  |  |
| Os03g0408600 | LOC_Os03g29480 | 3:16807288 | T | C | upstream |  |  |  |
| Os03g0408600 | LOC_Os03g29480 | 3:16807321 | C | T | upstream |  |  |  |
| Os03g0408600 | LOC_Os03g29480 | 3:16807331 | A | G | upstream |  |  |  |
| Os03g0408600 | LOC_Os03g29480 | 3:16807398 | T | C | upstream |  |  |  |
| Os03g0408600 | LOC_Os03g29480 | 3:16807441 | A | G | upstream |  |  |  |
| Os03g0408600 | LOC_Os03g29480 | 3:16807610 | G | A | upstream |  |  |  |
| Os03g0408600 | LOC_Os03g29480 | 3:16807770 | A | C | upstream |  | 8.49E-07 |  |
| Os03g0408600 | LOC_Os03g29480 | 3:16807874 | C | G | upstream |  |  |  |
| Os03g0408600 | LOC_Os03g29480 | 3:16807899 | C | T | upstream |  |  |  |
| Os03g0408600 | LOC_Os03g29480 | 3:16807904 | A | G | upstream |  |  |  |
| Os03g0408600 | LOC_Os03g29480 | 3:16807958 | C | T | upstream |  |  |  |
| Os03g0408600 | LOC_Os03g29480 | 3:16808037 | G | T | upstream |  |  |  |
| Os03g0408600 | LOC_Os03g29480 | 3:16808088 | C | G | upstream |  |  |  |
| Os03g0408600 | LOC_Os03g29480 | 3:16808402 | C | A | upstream |  |  |  |
| Os03g0408600 | LOC_Os03g29480 | 3:16808724 | A | T | upstream |  |  |  |
| Os03g0408600 | LOC_Os03g29480 | 3:16809861 | C | T | upstream |  |  |  |
| Os03g0408600 | LOC_Os03g29480 | 3:16809890 | G | A | upstream |  |  |  |
| Os03g0408600 | LOC_Os03g29480 | 3:16809927 | G | A | upstream |  |  |  |
| Os03g0408600 | LOC_Os03g29480 | 3:16809967 | T | C | upstream |  |  |  |
| Os03g0408600 | LOC_Os03g29480 | 3:16810148 | A | G | upstream |  |  |  |
| Os03g0408600 | LOC_Os03g29480 | 3:16810243 | C | T | upstream |  |  |  |
| Os03g0408600 | LOC_Os03g29480 | 3:16810349 | T | C | upstream |  |  |  |
| Os03g0408600 | LOC_Os03g29480 | 3:16810442 | T | A | upstream |  |  |  |
| Os03g0408600 | LOC_Os03g29480 | 3:16810830 | A | T | upstream |  |  |  |
| Os03g0409100 | LOC_Os03g29540 | 3:16831760 | C | T | downstream |  |  |  |
| Os03g0409100 | LOC_Os03g29540 | 3:16831839 | C | T | downstream |  |  |  |
| Os03g0409100 | LOC_Os03g29540 | 3:16832130 | A | G | downstream |  |  |  |
| Os03g0409100 | LOC_Os03g29540 | 3:16832163 | G | A | downstream |  |  |  |
| Os03g0409100 | LOC_Os03g29540 | 3:16832300 | C | T | downstream |  |  |  |
| Os03g0409100 | LOC_Os03g29540 | 3:16833255 | A | G | downstream |  |  |  |
| Os03g0409100 | LOC_Os03g29540 | 3:16833326 | G | C | downstream |  |  |  |
| Os03g0409100 | LOC_Os03g29540 | 3:16833428 | A | C | downstream |  |  |  |
| Os03g0409100 | LOC_Os03g29540 | 3:16833463 | A | G | downstream |  |  |  |
| Os03g0409100 | LOC_Os03g29540 | 3:16833499 | G | T | downstream |  |  |  |
| Os03g0409100 | LOC_Os03g29540 | 3:16833632 | C | A | downstream |  |  |  |
| Os03g0409100 | LOC_Os03g29540 | 3:16833665 | G | A | downstream |  |  |  |
| Os03g0409100 | LOC_Os03g29540 | 3:16833737 | C | T | downstream |  |  |  |
| Os03g0409100 | LOC_Os03g29540 | 3:16833848 | G | A | downstream |  |  |  |
| Os03g0409100 | LOC_Os03g29540 | 3:16833882 | A | G | downstream |  |  |  |
| Os03g0409100 | LOC_Os03g29540 | 3:16834258 | G | A | downstream |  |  |  |
| Os03g0409100 | LOC_Os03g29540 | 3:16834275 | G | A | downstream |  |  |  |
| Os03g0409100 | LOC_Os03g29540 | 3:16834395 | T | C | downstream |  |  |  |
| Os03g0409100 | LOC_Os03g29540 | 3:16834460 | G | A | downstream |  |  |  |
| Os03g0409100 | LOC_Os03g29540 | 3:16834570 | T | A | downstream |  |  |  |
| Os03g0409100 | LOC_Os03g29540 | 3:16834573 | G | A | downstream |  |  |  |
| Os03g0409100 | LOC_Os03g29540 | 3:16834653 | A | C | downstream |  |  |  |
| Os03g0409100 | LOC_Os03g29540 | 3:16834657 | T | G | downstream |  |  |  |
| Os03g0409100 | LOC_Os03g29540 | 3:16834685 | A | G | downstream |  |  |  |
| Os03g0409100 | LOC_Os03g29540 | 3:16834732 | A | G | downstream |  |  |  |
| Os03g0409100 | LOC_Os03g29540 | 3:16834737 | G | A | downstream |  |  |  |
| Os03g0409100 | LOC_Os03g29540 | 3:16834840 | A | G | downstream |  |  |  |
| Os03g0409100 | LOC_Os03g29540 | 3:16834865 | G | T | downstream |  |  |  |
| Os03g0409100 | LOC_Os03g29540 | 3:16835104 | A | G | downstream |  |  |  |
| Os03g0409100 | LOC_Os03g29540 | 3:16835110 | G | A | downstream |  |  |  |
| Os03g0409100 | LOC_Os03g29540 | 3:16835171 | C | T | downstream |  |  |  |
| Os03g0409100 | LOC_Os03g29540 | 3:16835360 | A | G | downstream |  |  |  |
| Os03g0409100 | LOC_Os03g29540 | 3:16835391 | C | T | downstream |  |  |  |
| Os03g0409100 | LOC_Os03g29540 | 3:16835416 | C | T | downstream |  |  |  |
| Os03g0409100 | LOC_Os03g29540 | 3:16835528 | C | T | downstream |  |  |  |
| Os03g0409100 | LOC_Os03g29540 | 3:16835628 | T | G | downstream |  |  |  |
| Os03g0409100 | LOC_Os03g29540 | 3:16835868 | C | T | downstream |  |  |  |
| Os03g0409100 | LOC_Os03g29540 | 3:16835940 | G | A | downstream |  |  |  |
| Os03g0409100 | LOC_Os03g29540 | 3:16836048 | C | T | downstream |  |  |  |
| Os03g0409100 | LOC_Os03g29540 | 3:16836180 | T | C | downstream |  |  |  |
| Os03g0409100 | LOC_Os03g29540 | 3:16836210 | G | A | downstream |  |  |  |
| Os03g0409100 | LOC_Os03g29540 | 3:16836258 | C | T | downstream |  |  |  |
| Os03g0409100 | LOC_Os03g29540 | 3:16836271 | C | T | downstream |  |  |  |
| Os03g0409100 | LOC_Os03g29540 | 3:16836412 | T | A | downstream |  |  |  |
| Os03g0409100 | LOC_Os03g29540 | 3:16836490 | C | T | downstream |  |  |  |
| Os03g0409100 | LOC_Os03g29540 | 3:16836522 | A | G | downstream |  |  |  |
| Os03g0409100 | LOC_Os03g29540 | 3:16836628 | G | T | 3_prime_UTR |  |  |  |
| Os03g0409100 | LOC_Os03g29540 | 3:16837841 | C | G | exonic | nonsynonymous |  |  |
| Os03g0409100 | LOC_Os03g29540 | 3:16838056 | G | A | 5_prime_UTR |  |  |  |
| Os03g0409100 | LOC_Os03g29540 | 3:16838674 | C | T | 5_prime_UTR |  |  |  |
| Os03g0409100 | LOC_Os03g29540 | 3:16839001 | T | C | 5_prime_UTR |  |  |  |
| Os03g0409100 | LOC_Os03g29540 | 3:16839202 | A | G | 5_prime_UTR |  |  |  |
| Os03g0409100 | LOC_Os03g29540 | 3:16839609 | A | G | 5_prime_UTR |  |  |  |
| Os03g0409100 | LOC_Os03g29540 | 3:16839657 | C | T | 5_prime_UTR |  |  |  |
| Os03g0409100 | LOC_Os03g29540 | 3:16839688 | C | T | 5_prime_UTR |  |  |  |
| Os03g0409100 | LOC_Os03g29540 | 3:16839702 | C | G | 5_prime_UTR |  |  |  |
| Os03g0409100 | LOC_Os03g29540 | 3:16839860 | C | T | 5_prime_UTR |  |  |  |
| Os03g0409100 | LOC_Os03g29540 | 3:16839877 | G | A | 5_prime_UTR |  |  |  |
| Os03g0409100 | LOC_Os03g29540 | 3:16839992 | A | G | 5_prime_UTR |  |  |  |
| Os03g0409100 | LOC_Os03g29540 | 3:16840024 | C | T | 5_prime_UTR |  |  |  |
| Os03g0409100 | LOC_Os03g29540 | 3:16840055 | A | G | 5_prime_UTR |  |  |  |
| Os03g0409100 | LOC_Os03g29540 | 3:16840231 | C | T | 5_prime_UTR |  |  |  |
| Os03g0409100 | LOC_Os03g29540 | 3:16840245 | A | G | 5_prime_UTR |  |  |  |
| Os03g0409100 | LOC_Os03g29540 | 3:16840375 | A | G | 5_prime_UTR |  |  |  |
| Os03g0409100 | LOC_Os03g29540 | 3:16840381 | T | C | 5_prime_UTR |  |  |  |
| Os03g0409100 | LOC_Os03g29540 | 3:16840727 | A | T | 5_prime_UTR |  |  |  |
| Os03g0409100 | LOC_Os03g29540 | 3:16840795 | A | T | upstream |  |  |  |
| Os03g0409100 | LOC_Os03g29540 | 3:16842769 | T | C | upstream |  |  |  |
| Os03g0409100 | LOC_Os03g29540 | 3:16842798 | G | C | upstream |  |  |  |
| Os03g0409100 | LOC_Os03g29540 | 3:16843069 | T | G | upstream |  |  |  |
| Os03g0409100 | LOC_Os03g29540 | 3:16843210 | A | T | upstream |  |  |  |
| Os03g0409100 | LOC_Os03g29540 | 3:16843214 | C | T | upstream |  |  |  |
| Os03g0409100 | LOC_Os03g29540 | 3:16843306 | T | A | upstream |  |  |  |
| Os03g0409400 | LOC_Os03g29570 | 3:16857608 | T | C | exonic | nonsynonymous |  |  |
| Os03g0409400 | LOC_Os03g29570 | 3:16857666 | T | C | intron |  |  |  |
| Os03g0409400 | LOC_Os03g29570 | 3:16857726 | G | A | intron |  |  |  |
| Os03g0409400 | LOC_Os03g29570 | 3:16858546 | G | C | intron |  |  |  |
| Os03g0409400 | LOC_Os03g29570 | 3:16859040 | T | G | intron |  |  |  |
| Os03g0409400 | LOC_Os03g29570 | 3:16859041 | G | A | intron |  |  |  |
| Os03g0409400 | LOC_Os03g29570 | 3:16859055 | C | T | intron |  |  |  |
| Os03g0409400 | LOC_Os03g29570 | 3:16859140 | A | T | intron |  |  |  |
| Os03g0409400 | LOC_Os03g29570 | 3:16859213 | A | C | intron |  |  |  |
| Os03g0409400 | LOC_Os03g29570 | 3:16859313 | C | T | intron |  |  |  |
| Os03g0409400 | LOC_Os03g29570 | 3:16860881 | C | A | intron |  |  |  |
| Os03g0409400 | LOC_Os03g29570 | 3:16860965 | T | G | intron |  |  |  |
| Os03g0409400 | LOC_Os03g29570 | 3:16861320 | T | C | intron |  |  |  |
| Os03g0409400 | LOC_Os03g29570 | 3:16861432 | A | C | intron |  |  |  |
| Os03g0409400 | LOC_Os03g29570 | 3:16861484 | A | G | intron |  |  |  |
| Os03g0409400 | LOC_Os03g29570 | 3:16862715 | G | A | upstream |  |  |  |
| Os03g0409400 | LOC_Os03g29570 | 3:16862921 | A | G | upstream |  |  |  |
| Os03g0409400 | LOC_Os03g29570 | 3:16863040 | A | C | upstream |  |  |  |
| Os03g0409400 | LOC_Os03g29570 | 3:16863106 | G | A | upstream |  |  |  |
| Os03g0409400 | LOC_Os03g29570 | 3:16863128 | T | G | upstream |  |  |  |
| Os03g0409400 | LOC_Os03g29570 | 3:16863175 | G | A | upstream |  |  |  |
| Os03g0409400 | LOC_Os03g29570 | 3:16863291 | C | A | upstream |  |  |  |
| Os03g0409400 | LOC_Os03g29570 | 3:16863506 | C | T | upstream |  |  |  |
| Os03g0409400 | LOC_Os03g29570 | 3:16863508 | T | G | upstream |  |  |  |
| Os03g0409400 | LOC_Os03g29570 | 3:16865123 | G | C | upstream |  |  |  |
| Os03g0409400 | LOC_Os03g29570 | 3:16865169 | C | T | upstream |  |  |  |
| Os03g0409400 | LOC_Os03g29570 | 3:16865333 | C | A | upstream |  |  |  |
| Os03g0409400 | LOC_Os03g29570 | 3:16865335 | A | T | upstream |  |  |  |
| Os03g0409400 | LOC_Os03g29570 | 3:16865511 | G | A | upstream |  |  |  |
| Os03g0409400 | LOC_Os03g29570 | 3:16865578 | C | T | upstream |  |  |  |
| Os03g0409400 | LOC_Os03g29570 | 3:16865665 | C | T | upstream |  |  |  |
| Os03g0409400 | LOC_Os03g29570 | 3:16865666 | C | T | upstream |  |  |  |
| Os03g0409400 | LOC_Os03g29570 | 3:16865735 | G | A | upstream |  |  |  |
| Os03g0409400 | LOC_Os03g29570 | 3:16865736 | C | T | upstream |  |  |  |
| Os03g0409400 | LOC_Os03g29570 | 3:16865751 | A | C | upstream |  |  |  |
| Os03g0409400 | LOC_Os03g29570 | 3:16865836 | A | G | upstream |  |  |  |
| Os03g0409400 | LOC_Os03g29570 | 3:16865866 | G | A | upstream |  |  |  |
| Os03g0409400 | LOC_Os03g29570 | 3:16865937 | C | T | upstream |  |  |  |
| Os03g0409400 | LOC_Os03g29570 | 3:16865940 | T | C | upstream |  |  |  |
| Os03g0409400 | LOC_Os03g29570 | 3:16865956 | C | T | upstream |  |  |  |
| Os03g0409400 | LOC_Os03g29570 | 3:16865999 | T | C | upstream |  |  |  |
| Os03g0409400 | LOC_Os03g29570 | 3:16866003 | T | C | upstream |  |  |  |
| Os03g0409400 | LOC_Os03g29570 | 3:16866114 | T | A | upstream |  |  |  |
| Os03g0409400 | LOC_Os03g29570 | 3:16866156 | G | A | upstream |  |  |  |
| Os03g0409400 | LOC_Os03g29570 | 3:16866178 | G | A | upstream |  |  |  |
| Os03g0409400 | LOC_Os03g29570 | 3:16866218 | T | A | upstream |  |  |  |
| Os03g0409400 | LOC_Os03g29570 | 3:16866253 | T | C | upstream |  |  |  |
| Os03g0409400 | LOC_Os03g29570 | 3:16866254 | C | T | upstream |  |  |  |
| Os03g0409400 | LOC_Os03g29570 | 3:16866259 | C | T | upstream |  |  |  |
| Os03g0409400 | LOC_Os03g29570 | 3:16866267 | T | C | upstream |  |  |  |
| Os03g0409400 | LOC_Os03g29570 | 3:16866392 | G | A | upstream |  |  |  |
| Os03g0409400 | LOC_Os03g29570 | 3:16866404 | C | T | upstream |  |  |  |
| Os03g0409400 | LOC_Os03g29570 | 3:16866526 | G | T | upstream |  |  |  |
| Os03g0409400 | LOC_Os03g29570 | 3:16866547 | A | G | upstream |  |  |  |
| Os03g0409400 | LOC_Os03g29570 | 3:16866568 | C | T | upstream |  |  |  |
| Os03g0409400 | LOC_Os03g29570 | 3:16866569 | G | A | upstream |  |  |  |
| Os03g0409400 | LOC_Os03g29570 | 3:16866630 | C | G | upstream |  |  |  |
| Os03g0409400 | LOC_Os03g29570 | 3:16866632 | A | C | upstream |  |  |  |
| Os03g0409400 | LOC_Os03g29570 | 3:16867052 | C | A | upstream |  |  |  |
| Os03g0409400 | LOC_Os03g29570 | 3:16867053 | T | C | upstream |  |  |  |
| Os03g0409400 | LOC_Os03g29570 | 3:16867054 | C | T | upstream |  |  |  |
| Os03g0409400 | LOC_Os03g29570 | 3:16867188 | A | C | upstream |  |  |  |
| Os03g0409400 | LOC_Os03g29570 | 3:16867616 | C | A | upstream |  |  |  |
| Os03g0409500 | None | 3:16855936 | G | A | upstream |  |  |  |
| Os03g0409500 | None | 3:16855971 | C | A | upstream |  |  |  |
| Os03g0409500 | None | 3:16856077 | G | C | upstream |  |  |  |
| Os03g0409500 | None | 3:16856151 | G | C | upstream |  |  |  |
| Os03g0409500 | None | 3:16856167 | G | T | upstream |  |  |  |
| Os03g0409500 | None | 3:16856271 | T | G | upstream |  |  |  |
| Os03g0409500 | None | 3:16856475 | C | T | upstream |  |  |  |
| Os03g0409500 | None | 3:16856647 | A | C | upstream |  |  |  |
| Os03g0409500 | None | 3:16856752 | A | C | upstream |  |  |  |
| Os03g0409500 | None | 3:16856810 | G | T | upstream |  |  |  |
| Os03g0409500 | None | 3:16856935 | C | A | upstream |  |  |  |
| Os03g0409500 | None | 3:16856978 | T | C | upstream |  |  |  |
| Os03g0409500 | None | 3:16857557 | G | A | exonic | nonsynonymous |  |  |
| Os03g0409600 | LOC_Os03g29584 | 3:16861738 | G | T | upstream |  |  |  |
| Os03g0409600 | LOC_Os03g29584 | 3:16861796 | A | G | upstream |  |  |  |
| Os03g0409600 | LOC_Os03g29584 | 3:16861941 | C | T | upstream |  |  |  |
| Os03g0409600 | LOC_Os03g29584 | 3:16861963 | A | T | upstream |  |  |  |
| Os03g0409600 | LOC_Os03g29584 | 3:16861991 | C | A | upstream |  |  |  |
| Os03g0409600 | LOC_Os03g29584 | 3:16862051 | G | A | upstream |  |  |  |
| Os03g0409600 | LOC_Os03g29584 | 3:16862291 | A | G | upstream |  |  |  |
| Os03g0409600 | LOC_Os03g29584 | 3:16862386 | G | A | upstream |  |  |  |
| Os03g0409600 | LOC_Os03g29584 | 3:16862395 | A | G | upstream |  |  |  |
| Os03g0409600 | LOC_Os03g29584 | 3:16866746 | C | T | 5_prime_UTR |  |  |  |
| Os03g0409600 | LOC_Os03g29584 | 3:16866751 | C | G | 5_prime_UTR |  |  |  |
| Os03g0409600 | LOC_Os03g29584 | 3:16866957 | G | A | 5_prime_UTR |  |  |  |
| Os03g0409600 | LOC_Os03g29584 | 3:16867021 | C | T | 5_prime_UTR |  |  |  |
| Os03g0409600 | LOC_Os03g29584 | 3:16867236 | A | C | 5_prime_UTR |  |  |  |
| Os03g0409600 | LOC_Os03g29584 | 3:16867237 | C | T | 5_prime_UTR |  |  |  |
| Os03g0409600 | LOC_Os03g29584 | 3:16867537 | C | T | intron |  |  |  |
| Os03g0409600 | LOC_Os03g29584 | 3:16867687 | C | T | intron |  |  |  |
| Os03g0409600 | LOC_Os03g29584 | 3:16867705 | A | G | intron |  |  |  |
| Os03g0409600 | LOC_Os03g29584 | 3:16867717 | C | T | intron |  |  |  |
| Os03g0409600 | LOC_Os03g29584 | 3:16867726 | T | C | intron |  |  |  |
| Os03g0409600 | LOC_Os03g29584 | 3:16867751 | G | A | intron |  |  |  |
| Os03g0409600 | LOC_Os03g29584 | 3:16867754 | G | A | intron |  |  |  |
| Os03g0409600 | LOC_Os03g29584 | 3:16867797 | T | G | intron |  |  |  |
| Os03g0409600 | LOC_Os03g29584 | 3:16867798 | C | T | intron |  |  |  |
| Os03g0409600 | LOC_Os03g29584 | 3:16867801 | C | T | intron |  |  |  |
| Os03g0409600 | LOC_Os03g29584 | 3:16867809 | C | T | intron |  |  |  |
| Os03g0409600 | LOC_Os03g29584 | 3:16867903 | A | G | 5_prime_UTR |  |  |  |
| Os03g0409600 | LOC_Os03g29584 | 3:16867925 | G | A | 5_prime_UTR |  |  |  |
| Os03g0409600 | LOC_Os03g29584 | 3:16867928 | G | T | 5_prime_UTR |  |  |  |
| Os03g0409600 | LOC_Os03g29584 | 3:16867935 | T | C | 5_prime_UTR |  |  |  |
| Os03g0409600 | LOC_Os03g29584 | 3:16867961 | G | T | 5_prime_UTR |  |  |  |
| Os03g0409600 | LOC_Os03g29584 | 3:16867997 | T | A | 5_prime_UTR |  |  |  |
| Os03g0409600 | LOC_Os03g29584 | 3:16868076 | G | A | 5_prime_UTR |  |  |  |
| Os03g0409600 | LOC_Os03g29584 | 3:16868088 | T | A | 5_prime_UTR |  |  |  |
| Os03g0409600 | LOC_Os03g29584 | 3:16868096 | A | C | 5_prime_UTR |  |  |  |
| Os03g0409600 | LOC_Os03g29584 | 3:16868098 | G | A | 5_prime_UTR |  |  |  |
| Os03g0409600 | LOC_Os03g29584 | 3:16868112 | C | A | 5_prime_UTR |  |  |  |
| Os03g0409600 | LOC_Os03g29584 | 3:16868143 | C | T | 5_prime_UTR |  |  |  |
| Os03g0409600 | LOC_Os03g29584 | 3:16868209 | A | G | exonic | nonsynonymous |  |  |
| Os03g0409600 | LOC_Os03g29584 | 3:16868269 | C | T | exonic | nonsynonymous |  |  |
| Os03g0409600 | LOC_Os03g29584 | 3:16868305 | A | G | exonic | nonsynonymous |  |  |
| Os03g0409600 | LOC_Os03g29584 | 3:16868354 | C | T | exonic | synonymous |  |  |
| Os03g0409600 | LOC_Os03g29584 | 3:16868362 | A | G | exonic | nonsynonymous |  |  |
| Os03g0409600 | LOC_Os03g29584 | 3:16868390 | T | C | intron |  |  |  |
| Os03g0409600 | LOC_Os03g29584 | 3:16868418 | G | T | intron |  |  |  |
| Os03g0409600 | LOC_Os03g29584 | 3:16868479 | A | G | intron |  |  |  |
| Os03g0409600 | LOC_Os03g29584 | 3:16868508 | A | T | intron |  |  |  |
| Os03g0409600 | LOC_Os03g29584 | 3:16868554 | G | T | intron |  |  |  |
| Os03g0409600 | LOC_Os03g29584 | 3:16868695 | T | A | intron |  |  |  |
| Os03g0409600 | LOC_Os03g29584 | 3:16868704 | G | T | intron |  |  |  |
| Os03g0409600 | LOC_Os03g29584 | 3:16868799 | G | A | intron |  |  |  |
| Os03g0409600 | LOC_Os03g29584 | 3:16868806 | G | A | intron |  |  |  |
| Os03g0409600 | LOC_Os03g29584 | 3:16868894 | T | C | intron |  |  |  |
| Os03g0409600 | LOC_Os03g29584 | 3:16868919 | T | C | intron |  |  |  |
| Os03g0409600 | LOC_Os03g29584 | 3:16869013 | G | T | exonic | nonsynonymous |  |  |
| Os03g0409600 | LOC_Os03g29584 | 3:16869049 | A | G | 3_prime_UTR |  |  |  |
| Os03g0409600 | LOC_Os03g29584 | 3:16869072 | G | A | 3_prime_UTR |  |  |  |
| Os03g0409600 | LOC_Os03g29584 | 3:16869102 | T | C | 3_prime_UTR |  |  |  |
| Os03g0409600 | LOC_Os03g29584 | 3:16869162 | C | T | 3_prime_UTR |  |  |  |
| Os03g0409600 | LOC_Os03g29584 | 3:16869172 | T | C | 3_prime_UTR |  |  |  |
| Os03g0409600 | LOC_Os03g29584 | 3:16869173 | A | T | 3_prime_UTR |  |  |  |
| Os03g0409600 | LOC_Os03g29584 | 3:16869256 | G | T | downstream |  |  |  |
| Os03g0409600 | LOC_Os03g29584 | 3:16869272 | G | A | downstream |  |  |  |
| Os03g0409600 | LOC_Os03g29584 | 3:16869312 | A | G | downstream |  |  |  |
| Os03g0409600 | LOC_Os03g29584 | 3:16869328 | C | T | downstream |  |  |  |
| Os03g0409600 | LOC_Os03g29584 | 3:16869368 | T | A | downstream |  |  |  |
| Os03g0409600 | LOC_Os03g29584 | 3:16869449 | T | C | downstream |  |  |  |
| Os03g0409600 | LOC_Os03g29584 | 3:16869529 | G | A | downstream |  |  |  |
| Os03g0409600 | LOC_Os03g29584 | 3:16869571 | A | C | downstream |  |  |  |
| Os03g0409600 | LOC_Os03g29584 | 3:16869599 | G | T | downstream |  |  |  |
| Os03g0409600 | LOC_Os03g29584 | 3:16869645 | C | T | downstream |  |  |  |
| Os03g0409600 | LOC_Os03g29584 | 3:16869647 | G | T | downstream |  |  |  |
| Os03g0409600 | LOC_Os03g29584 | 3:16869652 | T | C | downstream |  |  |  |
| Os03g0409600 | LOC_Os03g29584 | 3:16869805 | C | T | downstream |  |  |  |
| Os03g0409600 | LOC_Os03g29584 | 3:16869954 | G | T | downstream |  |  |  |
