# Supplementary material for: OsSYL2 AA, an allele identified by gene‐based association, increases style length in rice (Oryza sativa L.)
Source: Plant J. 2020 Oct 30;104(6):1491–503. doi: 10.1111/tpj.15013 (PMC7821000; doi:10.1111/tpj.15013)
Supplement: Supplementary file 7 — Table S6. Candidate gene annotation in the linkage disequilibrium region 16.69–16.87 Mb associated with style length and the sum of stigma and style length traits. [file TPJ-104-1491-s007.docx]

**Table S6.** Candidate gene annotation in the LD region 16.69-16.87Mb associated with style length and the sum of stigma and style length traits.

| Number | Gene ID | MSU ID | Position | Annotation |
| --- | --- | --- | --- | --- |
| 1 | Os03g0406900 | LOC_Os03g29340 | 16,692,517-16,696,367 | domain of unknown function domain containing protein |
| 2 | Os03g0407000 | LOC_Os03g29350 | 16,696,295-16,700,996 | von Willebrand factor, type A domain containing protein |
| 3 | Os03g0407050 | LOC_Os03g29340 | 16,696,508-16,704,564 | hypothetical protein |
| 4 | Os03g0407100 | LOC_Os03g29360 | 16,715,256-16,715,939 | expressed protein |
| 5 | Os03g0407400 | None | 16,729,501-16,735,109 | TNFR/NGFR family cysteine-rich domain, Regulator of grain size and organ size |
| 6 | Os03g0407900 | LOC_Os03g29410 | 16,749,318-16,754,223 | serine/threonine protein kinase-like |
| 7 | Os03g0408101 | None | 16,749,739-16,753,479 | hypothetical protein |
| 8 | Os03g0408300 | LOC_Os03g29460 | 16,787,183-16,787,911 | 60S ribosomal protein |
| 9 | Os03g0408401 | None | 16,788,230-16,789,167 | non-protein coding transcript |
| 10 | Os03g0408500 | LOC_Os03g29470 | 16,794,238-16,799,483 | transcription initiation factor IID |
| 11 | Os03g0408600 | LOC_Os03g29480 | 16,804,298-16,805,869 | GRAS-domain transcription factor, Strigolactone (SL) biosynthesis |
| 12 | Os03g0409100 | LOC_Os03g29540 | 16,836,622-16,840,754 | ATP-dependent protease |
| 13 | Os03g0409400 | LOC_Os03g29570 | 16,856,520-16,862,685 | mps one binder kinase activator-like 1A |
| 14 | Os03g0409500 | None | 16,857,192-16,861,667 | hypothetical protein |
| 15 | Os03g0409600 | LOC_Os03g29584 | 16,866,702-16,869,195 | expressed protein |
