# Supplementary material for: OsSYL2 AA, an allele identified by gene‐based association, increases style length in rice (Oryza sativa L.)
Source: Plant J. 2020 Oct 30;104(6):1491–503. doi: 10.1111/tpj.15013 (PMC7821000; doi:10.1111/tpj.15013)
Supplement: Supplementary file 8 — Table S7. The single‐nucleotide polymorphism information in the 30.45–30.65 Mb candidate region for style length trait. [file TPJ-104-1491-s008.docx]

**Table S7**. The SNP information in the 30.45-30.65Mb candidate region for style length trait.

| RAP ID | MSU ID | SNP location | Reference | Alterative | Region | Variation type | Associated signal in GWAS (P-value) | Gene-based association with traits |
| --- | --- | --- | --- | --- | --- | --- | --- | --- |
|  |  |  |  |  |  |  |  |  |
| Os02g0730775 | None | 2:30450197 | T | C | upstream |  |  |  |
| Os02g0730775 | None | 2:30450208 | G | A | upstream |  |  |  |
| Os02g0730775 | None | 2:30450237 | C | T | upstream |  |  |  |
| Os02g0730775 | None | 2:30450476 | A | G | upstream |  |  |  |
| Os02g0730775 | None | 2:30450728 | G | A | upstream |  |  |  |
| Os02g0730775 | None | 2:30450881 | T | G | upstream |  |  |  |
| Os02g0730775 | None | 2:30450900 | A | G | upstream |  |  |  |
| Os02g0730775 | None | 2:30451119 | G | A | upstream |  |  |  |
| Os02g0730775 | None | 2:30451177 | C | T | upstream |  |  |  |
| Os02g0730775 | None | 2:30451271 | A | G | upstream |  |  |  |
| Os02g0730775 | None | 2:30451374 | A | G | upstream |  |  |  |
| Os02g0730775 | None | 2:30451470 | T | C | upstream |  |  |  |
| Os02g0730775 | None | 2:30451516 | A | G | upstream |  |  |  |
| Os02g0730775 | None | 2:30451754 | C | T | upstream |  |  |  |
| Os02g0730775 | None | 2:30451912 | T | G | upstream |  |  |  |
| Os02g0730775 | None | 2:30452151 | T | A | upstream |  |  |  |
| Os02g0730900 | LOC_Os02g49830 | 2:30453067 | C | G | upstream |  |  |  |
| Os02g0730900 | LOC_Os02g49830 | 2:30453361 | T | A | upstream |  |  |  |
| Os02g0730900 | LOC_Os02g49830 | 2:30453600 | T | A | upstream |  |  |  |
| Os02g0730900 | LOC_Os02g49830 | 2:30453845 | A | G | upstream |  |  |  |
| Os02g0730900 | LOC_Os02g49830 | 2:30454090 | T | A | upstream |  |  |  |
| Os02g0730900 | LOC_Os02g49830 | 2:30454092 | A | T | upstream |  |  |  |
| Os02g0730900 | LOC_Os02g49830 | 2:30454334 | T | A | upstream |  |  |  |
| Os02g0731050 | None | 2:30454734 | C | T | upstream |  |  |  |
| Os02g0731050 | None | 2:30454817 | A | T | upstream |  |  |  |
| Os02g0731050 | None | 2:30455006 | C | T | upstream |  |  |  |
| Os02g0731050 | None | 2:30455028 | A | G | upstream |  |  |  |
| Os02g0731050 | None | 2:30455036 | C | G | upstream |  |  |  |
| Os02g0731050 | None | 2:30455100 | T | A | upstream |  |  |  |
| Os02g0731050 | None | 2:30455315 | G | A | upstream |  |  |  |
| Os02g0731050 | None | 2:30455436 | C | T | upstream |  |  |  |
| Os02g0731050 | None | 2:30455444 | C | T | upstream |  |  |  |
| Os02g0731050 | None | 2:30455484 | C | T | upstream |  |  |  |
| Os02g0731050 | None | 2:30455525 | C | T | upstream |  |  |  |
| Os02g0731050 | None | 2:30455565 | T | C | upstream |  |  |  |
| Os02g0731050 | None | 2:30455592 | G | T | upstream |  |  |  |
| Os02g0731050 | None | 2:30455634 | A | G | upstream |  |  |  |
| Os02g0731050 | None | 2:30455693 | G | A | upstream |  |  |  |
| Os02g0731050 | None | 2:30455747 | T | G | upstream |  |  |  |
| Os02g0731050 | None | 2:30455893 | C | T | upstream |  |  |  |
| Os02g0731050 | None | 2:30455910 | C | T | upstream |  |  |  |
| Os02g0731050 | None | 2:30455927 | C | T | upstream |  |  |  |
| Os02g0731050 | None | 2:30455939 | G | A | upstream |  |  |  |
| Os02g0731050 | None | 2:30455943 | C | T | upstream |  |  |  |
| Os02g0731050 | None | 2:30456099 | G | A | upstream |  |  |  |
| Os02g0731200 | LOC_Os02g49840 | 2:30459741 | G | A | intron |  | 2.37E-06 |  |
| Os02g0731200 | LOC_Os02g49840 | 2:30459765 | C | T | intron |  |  |  |
| Os02g0731200 | LOC_Os02g49840 | 2:30460568 | C | T | intron |  |  |  |
| Os02g0731200 | LOC_Os02g49840 | 2:30460957 | T | C | intron |  |  |  |
| Os02g0731200 | LOC_Os02g49840 | 2:30461268 | T | A | intron |  |  |  |
| Os02g0731200 | LOC_Os02g49840 | 2:30462130 | A | G | intron |  |  |  |
| Os02g0731200 | LOC_Os02g49840 | 2:30462691 | A | G | 3_prime_UTR |  |  |  |
| Os02g0731300 | None | 2:30457457 | C | A | downstream |  |  |  |
| Os02g0731300 | None | 2:30457729 | A | G | downstream |  |  |  |
| Os02g0731300 | None | 2:30457741 | A | C | downstream |  |  |  |
| Os02g0731300 | None | 2:30457848 | A | T | downstream |  |  |  |
| Os02g0731300 | None | 2:30458703 | A | G | downstream |  |  |  |
| Os02g0731300 | None | 2:30458749 | T | A | downstream |  |  |  |
| Os02g0731300 | None | 2:30458797 | T | G | downstream |  |  |  |
| Os02g0731300 | None | 2:30458854 | G | A | downstream |  |  |  |
| Os02g0731300 | None | 2:30458886 | A | G | downstream |  |  |  |
| Os02g0731300 | None | 2:30458941 | G | A | downstream |  |  |  |
| Os02g0731300 | None | 2:30458970 | G | C | downstream |  |  |  |
| Os02g0731300 | None | 2:30459130 | C | G | downstream |  |  |  |
| Os02g0731300 | None | 2:30459136 | A | G | downstream |  |  |  |
| Os02g0731300 | None | 2:30459274 | G | A | downstream |  |  |  |
| Os02g0731300 | None | 2:30464445 | G | C | upstream |  |  |  |
| Os02g0731300 | None | 2:30464676 | C | T | upstream |  |  |  |
| Os02g0731300 | None | 2:30465008 | C | T | upstream |  |  |  |
| Os02g0731300 | None | 2:30465056 | G | A | upstream |  |  |  |
| Os02g0731300 | None | 2:30465075 | C | T | upstream |  |  |  |
| Os02g0731300 | None | 2:30465536 | T | A | upstream |  |  |  |
| Os02g0731300 | None | 2:30465867 | G | A | upstream |  |  |  |
| Os02g0731300 | None | 2:30465900 | G | A | upstream |  |  |  |
| Os02g0731300 | None | 2:30465920 | T | G | upstream |  |  |  |
| Os02g0731300 | None | 2:30465955 | C | T | upstream |  |  |  |
| Os02g0731300 | None | 2:30465959 | C | T | upstream |  |  |  |
| Os02g0731300 | None | 2:30466134 | T | C | upstream |  |  |  |
| Os02g0731300 | None | 2:30466245 | G | A | upstream |  |  |  |
| Os02g0731300 | None | 2:30466456 | C | T | upstream |  |  |  |
| Os02g0731300 | None | 2:30466469 | C | T | upstream |  |  |  |
| Os02g0731300 | None | 2:30466546 | G | A | upstream |  |  |  |
| Os02g0731300 | None | 2:30466626 | A | G | upstream |  |  |  |
| Os02g0731300 | None | 2:30466705 | G | A | upstream |  |  |  |
| Os02g0731300 | None | 2:30466714 | C | A | upstream |  |  |  |
| Os02g0731300 | None | 2:30467294 | A | C | upstream |  |  |  |
| Os02g0731300 | None | 2:30467512 | C | T | upstream |  |  |  |
| Os02g0731300 | None | 2:30467943 | T | C | upstream |  |  |  |
| Os02g0731300 | None | 2:30468552 | G | A | upstream |  |  |  |
| Os02g0731400 | LOC_Os02g49850 | 2:30462791 | G | T | upstream |  |  |  |
| Os02g0731400 | LOC_Os02g49850 | 2:30463089 | G | A | upstream |  |  |  |
| Os02g0731400 | LOC_Os02g49850 | 2:30463410 | A | G | upstream |  |  |  |
| Os02g0731400 | LOC_Os02g49850 | 2:30463983 | C | G | upstream |  |  |  |
| Os02g0731400 | LOC_Os02g49850 | 2:30463991 | C | A | upstream |  |  |  |
| Os02g0731400 | LOC_Os02g49850 | 2:30468227 | C | A | 3_prime_UTR |  |  |  |
| Os02g0731400 | LOC_Os02g49850 | 2:30468228 | T | A | 3_prime_UTR |  |  |  |
| Os02g0731500 | LOC_Os02g49860 | 2:30468924 | A | G | 3_prime_UTR |  |  |  |
| Os02g0731500 | LOC_Os02g49860 | 2:30469725 | T | G | exonic | synonymous |  |  |
| Os02g0731500 | LOC_Os02g49860 | 2:30469995 | C | T | exonic | synonymous |  |  |
| Os02g0731500 | LOC_Os02g49860 | 2:30470111 | C | T | upstream |  |  |  |
| Os02g0731500 | LOC_Os02g49860 | 2:30470380 | A | C | upstream |  |  |  |
| Os02g0731500 | LOC_Os02g49860 | 2:30470415 | G | A | upstream |  |  |  |
| Os02g0731500 | LOC_Os02g49860 | 2:30470422 | T | G | upstream |  |  |  |
| Os02g0731500 | LOC_Os02g49860 | 2:30470446 | A | G | upstream |  |  |  |
| Os02g0731500 | LOC_Os02g49860 | 2:30470496 | A | G | upstream |  |  |  |
| Os02g0731500 | LOC_Os02g49860 | 2:30470594 | C | T | upstream |  |  |  |
| Os02g0731500 | LOC_Os02g49860 | 2:30470650 | A | G | upstream |  |  |  |
| Os02g0731500 | LOC_Os02g49860 | 2:30470652 | G | A | upstream |  |  |  |
| Os02g0731500 | LOC_Os02g49860 | 2:30470784 | C | T | upstream |  |  |  |
| Os02g0731500 | LOC_Os02g49860 | 2:30470823 | C | T | upstream |  |  |  |
| Os02g0731500 | LOC_Os02g49860 | 2:30471036 | T | G | upstream |  |  |  |
| Os02g0731500 | LOC_Os02g49860 | 2:30471060 | G | A | upstream |  |  |  |
| Os02g0731500 | LOC_Os02g49860 | 2:30471104 | A | G | upstream |  |  |  |
| Os02g0731500 | LOC_Os02g49860 | 2:30471182 | C | T | upstream |  |  |  |
| Os02g0731500 | LOC_Os02g49860 | 2:30471183 | C | A | upstream |  |  |  |
| Os02g0731500 | LOC_Os02g49860 | 2:30471266 | C | A | upstream |  |  |  |
| Os02g0731500 | LOC_Os02g49860 | 2:30472168 | T | C | upstream |  |  |  |
| Os02g0731500 | LOC_Os02g49860 | 2:30472600 | C | T | upstream |  |  |  |
| Os02g0731500 | LOC_Os02g49860 | 2:30473137 | G | A | upstream |  |  |  |
| Os02g0731500 | LOC_Os02g49860 | 2:30473143 | A | C | upstream |  |  |  |
| Os02g0731500 | LOC_Os02g49860 | 2:30473335 | C | T | upstream |  |  |  |
| Os02g0731500 | LOC_Os02g49860 | 2:30473349 | T | G | upstream |  |  |  |
| Os02g0731500 | LOC_Os02g49860 | 2:30473399 | C | T | upstream |  |  |  |
| Os02g0731500 | LOC_Os02g49860 | 2:30473521 | C | A | upstream |  |  |  |
| Os02g0731500 | LOC_Os02g49860 | 2:30473607 | T | C | upstream |  |  |  |
| Os02g0731500 | LOC_Os02g49860 | 2:30473609 | G | A | upstream |  |  |  |
| Os02g0731500 | LOC_Os02g49860 | 2:30473634 | G | A | upstream |  |  |  |
| Os02g0731600 | LOC_Os02g49870 | 2:30469376 | A | G | upstream |  |  |  |
| Os02g0731600 | LOC_Os02g49870 | 2:30472459 | A | G | exonic | nonsynonymous |  |  |
| Os02g0731600 | LOC_Os02g49870 | 2:30472500 | C | T | exonic | synonymous |  |  |
| Os02g0731700 | LOC_Os02g49880 | 2:30473912 | T | C | 3_prime_UTR |  |  |  |
| Os02g0731700 | LOC_Os02g49880 | 2:30475862 | G | A | upstream |  |  |  |
| Os02g0731700 | LOC_Os02g49880 | 2:30476235 | A | G | upstream |  |  |  |
| Os02g0731700 | LOC_Os02g49880 | 2:30476293 | C | T | upstream |  |  |  |
| Os02g0731700 | LOC_Os02g49880 | 2:30476413 | T | G | upstream |  |  |  |
| Os02g0731700 | LOC_Os02g49880 | 2:30476816 | T | G | upstream |  |  |  |
| Os02g0731700 | LOC_Os02g49880 | 2:30476953 | T | G | upstream |  |  |  |
| Os02g0731700 | LOC_Os02g49880 | 2:30476984 | A | G | upstream |  |  |  |
| Os02g0731700 | LOC_Os02g49880 | 2:30477086 | A | T | upstream |  |  |  |
| Os02g0731700 | LOC_Os02g49880 | 2:30477468 | C | T | upstream |  |  |  |
| Os02g0731700 | LOC_Os02g49880 | 2:30477800 | A | G | upstream |  |  |  |
| Os02g0731700 | LOC_Os02g49880 | 2:30477991 | T | A | upstream |  |  |  |
| Os02g0731700 | LOC_Os02g49880 | 2:30478421 | G | C | upstream |  |  |  |
| Os02g0731700 | LOC_Os02g49880 | 2:30479001 | C | T | upstream |  |  |  |
| Os02g0731700 | LOC_Os02g49880 | 2:30479036 | T | G | upstream |  |  |  |
| Os02g0731700 | LOC_Os02g49880 | 2:30479127 | C | T | upstream |  |  |  |
| Os02g0731700 | LOC_Os02g49880 | 2:30479558 | T | A | upstream |  |  |  |
| Os02g0731700 | LOC_Os02g49880 | 2:30479566 | T | C | upstream |  |  |  |
| Os02g0731700 | LOC_Os02g49880 | 2:30479573 | C | A | upstream |  |  |  |
| Os02g0731700 | LOC_Os02g49880 | 2:30480110 | C | T | upstream |  |  |  |
| Os02g0731700 | LOC_Os02g49880 | 2:30480388 | A | G | upstream |  |  |  |
| Os02g0731700 | LOC_Os02g49880 | 2:30480402 | T | C | upstream |  |  |  |
| Os02g0731900 | LOC_Os02g49920 | 2:30495873 | C | T | upstream |  |  |  |
| Os02g0731900 | LOC_Os02g49920 | 2:30495923 | G | A | upstream |  |  |  |
| Os02g0731900 | LOC_Os02g49920 | 2:30496167 | G | A | upstream |  |  |  |
| Os02g0731900 | LOC_Os02g49920 | 2:30496195 | C | T | upstream |  |  |  |
| Os02g0731900 | LOC_Os02g49920 | 2:30496197 | A | T | upstream |  |  |  |
| Os02g0731900 | LOC_Os02g49920 | 2:30496487 | A | C | upstream |  |  |  |
| Os02g0731900 | LOC_Os02g49920 | 2:30496712 | C | T | upstream |  |  |  |
| Os02g0731900 | LOC_Os02g49920 | 2:30496731 | C | T | upstream |  |  |  |
| Os02g0731900 | LOC_Os02g49920 | 2:30496746 | C | T | upstream |  |  |  |
| Os02g0731900 | LOC_Os02g49920 | 2:30496766 | G | A | upstream |  |  |  |
| Os02g0731900 | LOC_Os02g49920 | 2:30496781 | A | T | upstream |  |  |  |
| Os02g0731900 | LOC_Os02g49920 | 2:30496864 | C | T | upstream |  |  |  |
| Os02g0731900 | LOC_Os02g49920 | 2:30496866 | C | T | upstream |  |  |  |
| Os02g0731900 | LOC_Os02g49920 | 2:30497099 | A | G | upstream |  |  |  |
| Os02g0731900 | LOC_Os02g49920 | 2:30497333 | C | T | upstream |  |  |  |
| Os02g0731900 | LOC_Os02g49920 | 2:30497433 | C | T | upstream |  |  |  |
| Os02g0731900 | LOC_Os02g49920 | 2:30497528 | G | A | upstream |  |  |  |
| Os02g0731900 | LOC_Os02g49920 | 2:30497671 | G | A | upstream |  |  |  |
| Os02g0731900 | LOC_Os02g49920 | 2:30497709 | C | T | upstream |  |  |  |
| Os02g0731900 | LOC_Os02g49920 | 2:30497748 | C | T | upstream |  |  |  |
| Os02g0731900 | LOC_Os02g49920 | 2:30497781 | G | T | upstream |  |  |  |
| Os02g0731900 | LOC_Os02g49920 | 2:30498013 | C | T | upstream |  |  |  |
| Os02g0731900 | LOC_Os02g49920 | 2:30498059 | C | A | upstream |  |  |  |
| Os02g0731900 | LOC_Os02g49920 | 2:30498179 | C | T | upstream |  |  |  |
| Os02g0731900 | LOC_Os02g49920 | 2:30498187 | G | A | upstream |  |  |  |
| Os02g0731900 | LOC_Os02g49920 | 2:30498218 | G | A | upstream |  |  |  |
| Os02g0731900 | LOC_Os02g49920 | 2:30498602 | T | A | upstream |  |  |  |
| Os02g0731900 | LOC_Os02g49920 | 2:30498627 | G | C | upstream |  |  |  |
| Os02g0731900 | LOC_Os02g49920 | 2:30499024 | C | A | upstream |  |  |  |
| Os02g0731900 | LOC_Os02g49920 | 2:30499110 | G | A | upstream |  |  |  |
| Os02g0731900 | LOC_Os02g49920 | 2:30499141 | A | T | upstream |  |  |  |
| Os02g0731900 | LOC_Os02g49920 | 2:30499166 | A | C | upstream |  |  |  |
| Os02g0731900 | LOC_Os02g49920 | 2:30499240 | C | T | upstream |  |  |  |
| Os02g0731900 | LOC_Os02g49920 | 2:30499245 | A | G | upstream |  |  |  |
| Os02g0731900 | LOC_Os02g49920 | 2:30499500 | A | G | upstream |  |  |  |
| Os02g0731900 | LOC_Os02g49920 | 2:30499575 | C | T | upstream |  |  |  |
| Os02g0731900 | LOC_Os02g49920 | 2:30499635 | G | A | upstream |  |  |  |
| Os02g0731900 | LOC_Os02g49920 | 2:30499668 | A | T | upstream |  |  |  |
| Os02g0731900 | LOC_Os02g49920 | 2:30500078 | T | C | upstream |  |  |  |
| Os02g0731900 | LOC_Os02g49920 | 2:30502121 | G | T | 3_prime_UTR |  |  |  |
| Os02g0731900 | LOC_Os02g49920 | 2:30503589 | T | C | downstream |  |  |  |
| Os02g0731900 | LOC_Os02g49920 | 2:30503949 | T | A | downstream |  |  |  |
| Os02g0731900 | LOC_Os02g49920 | 2:30503972 | G | A | downstream |  |  |  |
| Os02g0731900 | LOC_Os02g49920 | 2:30503982 | A | G | downstream |  |  |  |
| Os02g0731900 | LOC_Os02g49920 | 2:30504596 | C | A | downstream |  |  |  |
| Os02g0731900 | LOC_Os02g49920 | 2:30504633 | C | T | downstream |  |  |  |
| Os02g0731900 | LOC_Os02g49920 | 2:30504751 | A | G | downstream |  |  |  |
| Os02g0731900 | LOC_Os02g49920 | 2:30504780 | A | G | downstream |  |  |  |
| Os02g0731900 | LOC_Os02g49920 | 2:30504888 | A | G | downstream |  |  |  |
| Os02g0731900 | LOC_Os02g49920 | 2:30505056 | C | T | downstream |  |  |  |
| Os02g0731900 | LOC_Os02g49920 | 2:30505112 | G | C | downstream |  |  |  |
| Os02g0731900 | LOC_Os02g49920 | 2:30505365 | C | A | downstream |  |  |  |
| Os02g0731900 | LOC_Os02g49920 | 2:30505413 | G | A | downstream |  |  |  |
| Os02g0731900 | LOC_Os02g49920 | 2:30505436 | T | C | downstream |  |  |  |
| Os02g0731900 | LOC_Os02g49920 | 2:30505444 | G | A | downstream |  |  |  |
| Os02g0731900 | LOC_Os02g49920 | 2:30505518 | A | G | downstream |  |  |  |
| Os02g0731900 | LOC_Os02g49920 | 2:30506965 | A | G | downstream |  |  |  |
| Os02g0732200 | LOC_Os02g49950 | 2:30516535 | T | C | upstream |  |  |  |
| Os02g0732200 | LOC_Os02g49950 | 2:30516544 | G | A | upstream |  |  |  |
| Os02g0732200 | LOC_Os02g49950 | 2:30516554 | A | G | upstream |  |  |  |
| Os02g0732200 | LOC_Os02g49950 | 2:30516584 | C | T | upstream |  |  |  |
| Os02g0732200 | LOC_Os02g49950 | 2:30516708 | A | G | upstream |  |  |  |
| Os02g0732200 | LOC_Os02g49950 | 2:30516826 | A | T | upstream |  |  |  |
| Os02g0732200 | LOC_Os02g49950 | 2:30516919 | T | G | upstream |  |  |  |
| Os02g0732200 | LOC_Os02g49950 | 2:30516989 | C | T | upstream |  |  |  |
| Os02g0732200 | LOC_Os02g49950 | 2:30517124 | G | T | upstream |  |  |  |
| Os02g0732200 | LOC_Os02g49950 | 2:30517225 | C | A | upstream |  |  |  |
| Os02g0732200 | LOC_Os02g49950 | 2:30517230 | C | A | upstream |  |  |  |
| Os02g0732200 | LOC_Os02g49950 | 2:30517253 | T | C | upstream |  |  |  |
| Os02g0732200 | LOC_Os02g49950 | 2:30520558 | G | A | exonic | nonsynonymous |  |  |
| Os02g0732200 | LOC_Os02g49950 | 2:30521725 | T | G | exonic | synonymous |  |  |
| Os02g0732200 | LOC_Os02g49950 | 2:30522160 | C | G | 3_prime_UTR |  |  |  |
| Os02g0732250 | None | 2:30519236 | C | T | downstream |  |  |  |
| Os02g0732250 | None | 2:30519343 | A | C | downstream |  |  |  |
| Os02g0732250 | None | 2:30519522 | A | T | downstream |  |  |  |
| Os02g0732250 | None | 2:30521434 | A | C | exonic | nonsynonymous |  |  |
| Os02g0732250 | None | 2:30522439 | C | T | upstream |  |  |  |
| Os02g0732250 | None | 2:30522457 | A | G | upstream |  |  |  |
| Os02g0732250 | None | 2:30522503 | T | G | upstream |  |  |  |
| Os02g0732250 | None | 2:30522625 | T | C | upstream |  |  |  |
| Os02g0732250 | None | 2:30522805 | A | G | upstream |  |  |  |
| Os02g0732250 | None | 2:30522849 | A | G | upstream |  |  |  |
| Os02g0732250 | None | 2:30523143 | C | T | upstream |  |  |  |
| Os02g0732250 | None | 2:30523196 | C | T | upstream |  |  |  |
| Os02g0732250 | None | 2:30523197 | C | T | upstream |  |  |  |
| Os02g0732250 | None | 2:30523363 | A | G | upstream |  |  |  |
| Os02g0732250 | None | 2:30523622 | G | A | upstream |  |  |  |
| Os02g0732250 | None | 2:30525393 | C | T | upstream |  |  |  |
| Os02g0732250 | None | 2:30525577 | C | T | upstream |  |  |  |
| Os02g0732250 | None | 2:30525911 | G | T | upstream |  |  |  |
| Os02g0732250 | None | 2:30525953 | G | A | upstream |  |  |  |
| Os02g0732250 | None | 2:30526267 | C | T | upstream |  |  |  |
| Os02g0732250 | None | 2:30527135 | A | T | upstream |  |  |  |
| Os02g0732300 | LOC_Os02g49960 | 2:30519800 | A | T | upstream |  | 6.75E-08 |  |
| Os02g0732300 | LOC_Os02g49961 | 2:30519843 | G | A | upstream |  | 7.28E-08 |  |
| Os02g0732300 | LOC_Os02g49962 | 2:30520035 | G | A | upstream |  |  |  |
| Os02g0732300 | LOC_Os02g49963 | 2:30530607 | C | T | downstream |  |  |  |
| Os02g0732300 | LOC_Os02g49964 | 2:30530657 | G | A | downstream |  |  |  |
| Os02g0732300 | LOC_Os02g49965 | 2:30530686 | C | T | downstream |  |  |  |
| Os02g0732300 | LOC_Os02g49966 | 2:30530695 | A | G | downstream |  |  |  |
| Os02g0732300 | LOC_Os02g49967 | 2:30530881 | C | T | downstream |  |  |  |
| Os02g0732300 | LOC_Os02g49968 | 2:30530959 | A | C | downstream |  |  |  |
| Os02g0732300 | LOC_Os02g49969 | 2:30530976 | T | C | downstream |  |  |  |
| Os02g0732300 | LOC_Os02g49970 | 2:30531118 | A | G | downstream |  |  |  |
| Os02g0732300 | LOC_Os02g49971 | 2:30531129 | G | A | downstream |  |  |  |
| Os02g0732300 | LOC_Os02g49972 | 2:30531145 | G | A | downstream |  |  |  |
| Os02g0732300 | LOC_Os02g49973 | 2:30531214 | G | A | downstream |  |  |  |
| Os02g0732300 | LOC_Os02g49974 | 2:30531270 | A | T | downstream |  |  |  |
| Os02g0732300 | LOC_Os02g49975 | 2:30531408 | G | A | downstream |  |  |  |
| Os02g0732300 | LOC_Os02g49976 | 2:30531621 | A | G | downstream |  |  |  |
| Os02g0732350 | None | 2:30527240 | T | C | upstream |  |  |  |
| Os02g0732350 | None | 2:30527354 | C | T | upstream |  |  |  |
| Os02g0732350 | None | 2:30527412 | G | A | upstream |  |  |  |
| Os02g0732350 | None | 2:30527504 | C | T | upstream |  |  |  |
| Os02g0732350 | None | 2:30528094 | G | A | upstream |  |  |  |
| Os02g0732350 | None | 2:30528095 | G | A | upstream |  |  |  |
| Os02g0732350 | None | 2:30528266 | A | G | upstream |  |  |  |
| Os02g0732350 | None | 2:30529804 | T | C | upstream |  |  |  |
| Os02g0732350 | None | 2:30529933 | C | T | upstream |  |  |  |
| Os02g0732400 | LOC_Os02g49970 | 2:30530432 | A | G | 3_prime_UTR |  |  |  |
| Os02g0732400 | LOC_Os02g49970 | 2:30531759 | T | A | downstream |  |  |  |
| Os02g0732400 | LOC_Os02g49970 | 2:30531766 | C | T | downstream |  |  |  |
| Os02g0732400 | LOC_Os02g49970 | 2:30532117 | T | A | downstream |  |  |  |
| Os02g0732400 | LOC_Os02g49970 | 2:30532130 | C | A | downstream |  |  |  |
| Os02g0732400 | LOC_Os02g49970 | 2:30532144 | G | A | downstream |  |  |  |
| Os02g0732400 | LOC_Os02g49970 | 2:30532362 | T | C | downstream |  |  |  |
| Os02g0732400 | LOC_Os02g49970 | 2:30532408 | C | T | downstream |  |  |  |
| Os02g0732400 | LOC_Os02g49970 | 2:30532600 | A | G | downstream |  |  |  |
| Os02g0732400 | LOC_Os02g49970 | 2:30532626 | C | A | downstream |  |  |  |
| Os02g0732400 | LOC_Os02g49970 | 2:30532672 | G | A | downstream |  |  |  |
| Os02g0732400 | LOC_Os02g49970 | 2:30532709 | A | G | downstream |  |  |  |
| Os02g0732400 | LOC_Os02g49970 | 2:30532744 | G | A | downstream |  |  |  |
| Os02g0732400 | LOC_Os02g49970 | 2:30533140 | G | T | downstream |  |  |  |
| Os02g0732400 | LOC_Os02g49970 | 2:30533176 | G | A | downstream |  |  |  |
| Os02g0732400 | LOC_Os02g49970 | 2:30533286 | C | T | downstream |  |  |  |
| Os02g0732400 | LOC_Os02g49970 | 2:30534236 | G | A | downstream |  |  |  |
| Os02g0732400 | LOC_Os02g49970 | 2:30534390 | T | G | downstream |  |  |  |
| Os02g0732400 | LOC_Os02g49970 | 2:30534402 | A | G | downstream |  |  |  |
| Os02g0732500 | LOC_Os02g49980 | 2:30535037 | T | A | exonic | synonymous |  |  |
| Os02g0732500 | LOC_Os02g49980 | 2:30536901 | T | G | upstream |  |  |  |
| Os02g0732500 | LOC_Os02g49980 | 2:30536907 | C | A | upstream |  |  |  |
| Os02g0732500 | LOC_Os02g49980 | 2:30537000 | C | T | upstream |  |  |  |
| Os02g0732500 | LOC_Os02g49980 | 2:30537024 | A | G | upstream |  |  |  |
| Os02g0732500 | LOC_Os02g49980 | 2:30537226 | C | T | upstream |  |  |  |
| Os02g0732500 | LOC_Os02g49980 | 2:30537865 | G | T | upstream |  |  |  |
| Os02g0732500 | LOC_Os02g49980 | 2:30538216 | G | A | upstream |  |  |  |
| Os02g0732500 | LOC_Os02g49980 | 2:30538537 | A | T | upstream |  |  |  |
| Os02g0732500 | LOC_Os02g49980 | 2:30538645 | T | A | upstream |  |  |  |
| Os02g0732500 | LOC_Os02g49980 | 2:30539273 | G | C | upstream |  |  |  |
| Os02g0732500 | LOC_Os02g49980 | 2:30539585 | A | C | upstream |  |  |  |
| Os02g0732500 | LOC_Os02g49980 | 2:30539629 | T | C | upstream |  |  |  |
| Os02g0732500 | LOC_Os02g49980 | 2:30540575 | T | C | upstream |  |  |  |
| Os02g0732500 | LOC_Os02g49980 | 2:30540803 | C | T | upstream |  |  |  |
| Os02g0732600 | LOC_Os02g49986 | 2:30535605 | T | C | downstream |  |  |  |
| Os02g0732600 | LOC_Os02g49986 | 2:30535722 | C | T | downstream |  |  |  |
| Os02g0732600 | LOC_Os02g49986 | 2:30535815 | G | T | downstream |  |  |  |
| Os02g0732600 | LOC_Os02g49986 | 2:30535855 | G | A | downstream |  |  |  |
| Os02g0732600 | LOC_Os02g49986 | 2:30535861 | T | A | downstream |  |  |  |
| Os02g0732600 | LOC_Os02g49986 | 2:30536159 | G | A | downstream |  |  |  |
| Os02g0732600 | LOC_Os02g49986 | 2:30541604 | G | A | upstream |  |  |  |
| Os02g0732600 | LOC_Os02g49986 | 2:30541924 | C | G | upstream |  |  |  |
| Os02g0732600 | LOC_Os02g49986 | 2:30542118 | T | C | upstream |  |  |  |
| Os02g0732600 | LOC_Os02g49986 | 2:30543427 | A | G | upstream |  |  |  |
| Os02g0732600 | LOC_Os02g49986 | 2:30543530 | A | G | upstream |  |  |  |
| Os02g0732600 | LOC_Os02g49986 | 2:30545020 | T | C | upstream |  |  |  |
| Os02g0732600 | LOC_Os02g49986 | 2:30545136 | T | C | upstream |  |  |  |
| Os02g0732600 | LOC_Os02g49986 | 2:30545380 | G | A | upstream |  |  |  |
| Os02g0732600 | LOC_Os02g49986 | 2:30545477 | G | A | upstream |  |  |  |
| Os02g0732600 | LOC_Os02g49986 | 2:30545819 | T | C | upstream |  |  |  |
| Os02g0732700 | LOC_Os02g49992 | 2:30542494 | T | G | 3_prime_UTR |  |  |  |
| Os02g0732700 | LOC_Os02g49992 | 2:30544191 | G | A | exonic | synonymous |  |  |
| Os02g0732700 | LOC_Os02g49992 | 2:30544852 | G | A | exonic | synonymous |  |  |
| Os02g0732700 | LOC_Os02g49992 | 2:30546066 | C | T | exonic | nonsynonymous |  |  |
| Os02g0732700 | LOC_Os02g49992 | 2:30547004 | A | T | exonic | nonsynonymous |  |  |
| Os02g0732700 | LOC_Os02g49992 | 2:30547528 | G | A | downstream |  |  |  |
| Os02g0732700 | LOC_Os02g49992 | 2:30547700 | G | A | downstream |  |  |  |
| Os02g0732700 | LOC_Os02g49992 | 2:30547720 | T | C | downstream |  |  |  |
| Os02g0732700 | LOC_Os02g49992 | 2:30548053 | A | C | downstream |  |  |  |
| Os02g0732700 | LOC_Os02g49992 | 2:30548353 | A | T | upstream |  |  |  |
| Os02g0732700 | LOC_Os02g49992 | 2:30548382 | T | G | upstream |  |  |  |
| Os02g0732700 | LOC_Os02g49992 | 2:30548931 | G | A | upstream |  |  |  |
| Os02g0732700 | LOC_Os02g49992 | 2:30549210 | T | C | upstream |  |  |  |
| Os02g0732700 | LOC_Os02g49992 | 2:30549861 | A | T | intron |  |  |  |
| Os02g0732700 | LOC_Os02g49992 | 2:30549880 | C | T | exonic | nonsynonymous |  |  |
| Os02g0732700 | LOC_Os02g49992 | 2:30550182 | G | T | upstream |  |  |  |
| Os02g0732700 | LOC_Os02g49992 | 2:30550849 | T | G | upstream |  |  |  |
| Os02g0732700 | LOC_Os02g49992 | 2:30551059 | G | A | exonic | synonymous |  |  |
| Os02g0732700 | LOC_Os02g49992 | 2:30551213 | T | G | upstream |  |  |  |
| Os02g0732700 | LOC_Os02g49992 | 2:30551727 | G | A | exonic | nonsynonymous |  |  |
| Os02g0732700 | LOC_Os02g49992 | 2:30551973 | A | T | exonic | nonsynonymous |  |  |
| Os02g0732700 | LOC_Os02g49992 | 2:30552335 | A | G | exonic | synonymous |  |  |
| Os02g0732700 | LOC_Os02g49992 | 2:30553643 | G | T | upstream |  |  |  |
| Os02g0732700 | LOC_Os02g49992 | 2:30553990 | C | T | upstream |  |  |  |
| Os02g0732700 | LOC_Os02g49992 | 2:30554053 | G | A | upstream |  |  |  |
| Os02g0732700 | LOC_Os02g49992 | 2:30554239 | C | T | upstream |  |  |  |
| Os02g0732700 | LOC_Os02g49992 | 2:30554265 | A | G | upstream |  |  |  |
| Os02g0732700 | LOC_Os02g49992 | 2:30554299 | G | A | upstream |  |  |  |
| Os02g0732700 | LOC_Os02g49992 | 2:30555317 | C | T | upstream |  |  |  |
| Os02g0732700 | LOC_Os02g49992 | 2:30555976 | A | G | upstream |  |  |  |
| Os02g0732700 | LOC_Os02g49992 | 2:30556465 | T | C | upstream |  |  |  |
| Os02g0732700 | LOC_Os02g49992 | 2:30556501 | A | G | upstream |  |  |  |
| Os02g0732700 | LOC_Os02g49992 | 2:30556860 | C | T | upstream |  |  |  |
| Os02g0732700 | LOC_Os02g49992 | 2:30557074 | G | A | upstream |  |  |  |
| Os02g0732700 | LOC_Os02g49992 | 2:30557276 | T | G | upstream |  |  |  |
| Os02g0732700 | LOC_Os02g49992 | 2:30557891 | G | A | upstream |  |  |  |
| Os02g0732700 | LOC_Os02g49992 | 2:30558476 | A | C | upstream |  |  |  |
| Os02g0732800 | LOC_Os02g50000 | 2:30559044 | T | C | upstream |  |  |  |
| Os02g0732800 | LOC_Os02g50000 | 2:30559059 | C | A | upstream |  |  |  |
| Os02g0732800 | LOC_Os02g50000 | 2:30559736 | C | T | upstream |  |  |  |
| Os02g0732800 | LOC_Os02g50000 | 2:30560960 | T | G | upstream |  |  |  |
| Os02g0732900 | LOC_Os02g50010 | 2:30552961 | G | C | upstream |  |  |  |
| Os02g0732900 | LOC_Os02g50010 | 2:30557751 | T | G | exonic | synonymous |  |  |
| Os02g0732900 | LOC_Os02g50010 | 2:30558014 | G | A | intron |  |  |  |
| Os02g0732900 | LOC_Os02g50010 | 2:30558760 | A | G | exonic | nonsynonymous |  |  |
| Os02g0732900 | LOC_Os02g50010 | 2:30561633 | G | A | downstream |  |  |  |
| Os02g0732900 | LOC_Os02g50010 | 2:30561850 | G | A | downstream |  |  |  |
| Os02g0732900 | LOC_Os02g50010 | 2:30561972 | C | T | downstream |  |  |  |
| Os02g0732900 | LOC_Os02g50010 | 2:30561976 | C | T | downstream |  |  |  |
| Os02g0732900 | LOC_Os02g50010 | 2:30562164 | T | C | downstream |  |  |  |
| Os02g0732900 | LOC_Os02g50010 | 2:30562193 | G | A | downstream |  |  |  |
| Os02g0732900 | LOC_Os02g50010 | 2:30562229 | G | A | downstream |  |  |  |
| Os02g0733001 | LOC_Os02g50020 | 2:30564398 | A | G | upstream |  |  |  |
| Os02g0733001 | LOC_Os02g50020 | 2:30564584 | T | C | upstream |  |  |  |
| Os02g0733001 | LOC_Os02g50020 | 2:30564762 | C | G | upstream |  |  |  |
| Os02g0733001 | LOC_Os02g50020 | 2:30564891 | C | A | upstream |  |  |  |
| Os02g0733001 | LOC_Os02g50020 | 2:30564907 | G | A | upstream |  |  |  |
| Os02g0733001 | LOC_Os02g50020 | 2:30565086 | G | A | upstream |  |  |  |
| Os02g0733001 | LOC_Os02g50020 | 2:30565288 | T | C | upstream |  |  |  |
| Os02g0733001 | LOC_Os02g50020 | 2:30565377 | G | A | upstream |  |  |  |
| Os02g0733001 | LOC_Os02g50020 | 2:30565396 | G | A | upstream |  |  |  |
| Os02g0733001 | LOC_Os02g50020 | 2:30565455 | A | G | upstream |  |  |  |
| Os02g0733001 | LOC_Os02g50020 | 2:30565536 | A | G | upstream |  |  |  |
| Os02g0733001 | LOC_Os02g50020 | 2:30565574 | G | A | upstream |  |  |  |
| Os02g0733001 | LOC_Os02g50020 | 2:30565651 | A | G | upstream |  |  |  |
| Os02g0733001 | LOC_Os02g50020 | 2:30565897 | A | G | upstream |  |  |  |
| Os02g0733001 | LOC_Os02g50020 | 2:30566952 | C | T | upstream |  |  |  |
| Os02g0733001 | LOC_Os02g50020 | 2:30566984 | G | A | upstream |  |  |  |
| Os02g0733001 | LOC_Os02g50020 | 2:30567163 | A | T | upstream |  |  |  |
| Os02g0733001 | LOC_Os02g50020 | 2:30568216 | G | T | upstream |  |  |  |
| Os02g0733001 | LOC_Os02g50020 | 2:30568296 | G | T | upstream |  |  |  |
| Os02g0733001 | LOC_Os02g50020 | 2:30568298 | A | T | upstream |  |  |  |
| Os02g0733166 | None | 2:30567274 | C | T | exonic | nonsynonymous |  |  |
| Os02g0733166 | None | 2:30567386 | G | A | exonic | synonymous |  |  |
| Os02g0733166 | None | 2:30570559 | G | A | upstream |  |  |  |
| Os02g0733166 | None | 2:30570592 | A | C | upstream |  |  |  |
| Os02g0733166 | None | 2:30570641 | G | A | upstream |  |  |  |
| Os02g0733166 | None | 2:30570713 | A | C | upstream |  |  |  |
| Os02g0733166 | None | 2:30570714 | A | T | upstream |  |  |  |
| Os02g0733166 | None | 2:30570742 | G | T | upstream |  |  |  |
| Os02g0733166 | None | 2:30570746 | A | G | upstream |  |  |  |
| Os02g0733166 | None | 2:30571075 | T | C | upstream |  |  |  |
| Os02g0733166 | None | 2:30571110 | T | C | upstream |  |  |  |
| Os02g0733166 | None | 2:30571181 | C | T | upstream |  |  |  |
| Os02g0733166 | None | 2:30571192 | C | T | upstream |  |  |  |
| Os02g0733166 | None | 2:30571236 | C | T | upstream |  |  |  |
| Os02g0733166 | None | 2:30571285 | C | T | upstream |  |  |  |
| Os02g0733166 | None | 2:30571345 | T | C | upstream |  |  |  |
| Os02g0733166 | None | 2:30571779 | T | C | upstream |  |  |  |
| Os02g0733166 | None | 2:30571791 | G | A | upstream |  |  |  |
| Os02g0733166 | None | 2:30572105 | G | A | upstream |  |  |  |
| Os02g0733166 | None | 2:30572424 | T | C | upstream |  |  |  |
| Os02g0733200 | None | 2:30562371 | T | C | upstream |  |  |  |
| Os02g0733200 | None | 2:30562461 | A | C | upstream |  |  |  |
| Os02g0733200 | None | 2:30562841 | A | G | upstream |  |  |  |
| Os02g0733200 | None | 2:30574904 | T | C | downstream |  |  |  |
| Os02g0733300 | LOC_Os02g50040 | 2:30572918 | C | T | upstream |  |  |  |
| Os02g0733300 | LOC_Os02g50040 | 2:30573182 | A | T | upstream |  |  |  |
| Os02g0733300 | LOC_Os02g50040 | 2:30577252 | G | T | 3_prime_UTR |  |  |  |
| Os02g0733300 | LOC_Os02g50040 | 2:30577311 | C | T | downstream |  |  |  |
| Os02g0733300 | LOC_Os02g50040 | 2:30577550 | A | T | downstream |  |  |  |
| Os02g0733400 | LOC_Os02g50050 | 2:30574984 | A | G | downstream |  |  |  |
| Os02g0733400 | LOC_Os02g50050 | 2:30578381 | G | A | 5_prime_UTR |  |  |  |
| Os02g0733400 | LOC_Os02g50050 | 2:30578412 | T | A | upstream |  |  |  |
| Os02g0733400 | LOC_Os02g50050 | 2:30578448 | T | G | upstream |  |  |  |
| Os02g0733400 | LOC_Os02g50050 | 2:30578518 | A | G | upstream |  |  |  |
| Os02g0733400 | LOC_Os02g50050 | 2:30578694 | T | A | upstream |  |  |  |
| Os02g0733400 | LOC_Os02g50050 | 2:30578711 | T | C | upstream |  |  |  |
| Os02g0733400 | LOC_Os02g50050 | 2:30578730 | C | A | upstream |  |  |  |
| Os02g0733400 | LOC_Os02g50050 | 2:30579527 | C | A | upstream |  | 1.76E-08 |  |
| Os02g0733400 | LOC_Os02g50050 | 2:30579820 | G | C | upstream |  |  |  |
| Os02g0733400 | LOC_Os02g50050 | 2:30580985 | T | C | upstream |  |  |  |
| Os02g0733400 | LOC_Os02g50050 | 2:30581064 | A | G | upstream |  |  |  |
| Os02g0733400 | LOC_Os02g50050 | 2:30581099 | G | A | upstream |  |  |  |
| Os02g0733400 | LOC_Os02g50050 | 2:30581189 | C | T | upstream |  |  |  |
| Os02g0733400 | LOC_Os02g50050 | 2:30581275 | G | T | upstream |  |  |  |
| Os02g0733400 | LOC_Os02g50050 | 2:30581330 | C | T | upstream |  |  |  |
| Os02g0733400 | LOC_Os02g50050 | 2:30581375 | A | T | upstream |  |  |  |
| Os02g0733400 | LOC_Os02g50050 | 2:30581453 | G | A | upstream |  |  |  |
| Os02g0733400 | LOC_Os02g50050 | 2:30581576 | T | A | upstream |  |  |  |
| Os02g0733400 | LOC_Os02g50050 | 2:30581580 | A | G | upstream |  |  |  |
| Os02g0733400 | LOC_Os02g50050 | 2:30581643 | C | T | upstream |  |  |  |
| Os02g0733400 | LOC_Os02g50050 | 2:30581683 | G | A | upstream |  |  |  |
| Os02g0733400 | LOC_Os02g50050 | 2:30581725 | A | T | upstream |  |  |  |
| Os02g0733400 | LOC_Os02g50050 | 2:30581783 | A | G | upstream |  |  |  |
| Os02g0733400 | LOC_Os02g50050 | 2:30581787 | C | T | upstream |  |  |  |
| Os02g0733400 | LOC_Os02g50050 | 2:30581832 | C | T | upstream |  |  |  |
| Os02g0733400 | LOC_Os02g50050 | 2:30581836 | C | T | upstream |  |  |  |
| Os02g0733400 | LOC_Os02g50050 | 2:30581839 | G | A | upstream |  |  |  |
| Os02g0733400 | LOC_Os02g50050 | 2:30581856 | C | T | upstream |  |  |  |
| Os02g0733400 | LOC_Os02g50050 | 2:30581948 | T | C | upstream |  |  |  |
| Os02g0733400 | LOC_Os02g50050 | 2:30581954 | A | G | upstream |  |  |  |
| Os02g0733400 | LOC_Os02g50050 | 2:30582453 | G | A | upstream |  |  |  |
| Os02g0733500 | LOC_Os02g50060 | 2:30580219 | T | C | 3_prime_UTR |  | 2.04E-06 |  |
| Os02g0733500 | LOC_Os02g50060 | 2:30583758 | A | G | upstream |  |  |  |
| Os02g0733500 | LOC_Os02g50060 | 2:30583797 | T | A | upstream |  |  |  |
| Os02g0733500 | LOC_Os02g50060 | 2:30583858 | A | G | upstream |  |  |  |
| Os02g0733500 | LOC_Os02g50060 | 2:30584221 | G | A | upstream |  |  |  |
| Os02g0733500 | LOC_Os02g50060 | 2:30584229 | G | A | upstream |  |  |  |
| Os02g0733500 | LOC_Os02g50060 | 2:30584293 | C | T | upstream |  |  |  |
| Os02g0733500 | LOC_Os02g50060 | 2:30584380 | C | T | upstream |  |  |  |
| Os02g0733500 | LOC_Os02g50060 | 2:30584516 | C | T | upstream |  |  |  |
| Os02g0733500 | LOC_Os02g50060 | 2:30584719 | T | C | upstream |  |  |  |
| Os02g0733500 | LOC_Os02g50060 | 2:30585002 | C | T | upstream |  |  |  |
| Os02g0733500 | LOC_Os02g50060 | 2:30585408 | T | C | upstream |  |  |  |
| Os02g0733500 | LOC_Os02g50060 | 2:30585472 | C | T | upstream |  |  |  |
| Os02g0733800 | LOC_Os02g50100 | 2:30592534 | T | C | upstream |  |  |  |
| Os02g0733800 | LOC_Os02g50100 | 2:30592538 | G | T | upstream |  |  |  |
| Os02g0733800 | LOC_Os02g50100 | 2:30592600 | T | C | upstream |  |  |  |
| Os02g0733800 | LOC_Os02g50100 | 2:30592720 | A | G | upstream |  |  |  |
| Os02g0733800 | LOC_Os02g50100 | 2:30592804 | C | G | upstream |  |  |  |
| Os02g0733800 | LOC_Os02g50100 | 2:30592856 | G | T | upstream |  |  |  |
| Os02g0733800 | LOC_Os02g50100 | 2:30592957 | C | T | upstream |  |  |  |
| Os02g0733800 | LOC_Os02g50100 | 2:30593003 | T | C | upstream |  |  |  |
| Os02g0733800 | LOC_Os02g50100 | 2:30593010 | C | T | upstream |  |  |  |
| Os02g0733800 | LOC_Os02g50100 | 2:30593070 | T | C | upstream |  |  |  |
| Os02g0733800 | LOC_Os02g50100 | 2:30593349 | G | T | upstream |  |  |  |
| Os02g0733800 | LOC_Os02g50100 | 2:30593872 | C | T | exonic | synonymous |  |  |
| Os02g0733900 | LOC_Os02g50110 | 2:30594441 | C | T | downstream |  |  |  |
| Os02g0733900 | LOC_Os02g50110 | 2:30595105 | A | T | downstream |  |  |  |
| Os02g0733900 | LOC_Os02g50110 | 2:30595594 | G | T | downstream |  |  |  |
| Os02g0733900 | LOC_Os02g50110 | 2:30595614 | A | T | downstream |  |  |  |
| Os02g0733900 | LOC_Os02g50110 | 2:30596461 | A | G | 3_prime_UTR |  |  |  |
| **Os02g0733900** | **LOC_Os02g50110** | **2:30596777** | **G** | **T** | **exonic** | **nonsynonymous** | **4.56E-07** | **9.06E-05** |
| Os02g0733900 | LOC_Os02g50110 | 2:30596890 | C | G | exonic | nonsynonymous |  |  |
| Os02g0733900 | LOC_Os02g50110 | 2:30596917 | C | A | 5_prime_UTR |  |  |  |
| Os02g0733900 | LOC_Os02g50110 | 2:30597020 | G | T | upstream |  |  |  |
| Os02g0733900 | LOC_Os02g50110 | 2:30597044 | A | G | upstream |  |  |  |
| Os02g0733900 | LOC_Os02g50110 | 2:30597376 | T | A | upstream |  |  |  |
| Os02g0733900 | LOC_Os02g50110 | 2:30597552 | T | C | upstream |  |  |  |
| Os02g0733900 | LOC_Os02g50110 | 2:30597575 | T | C | upstream |  |  |  |
| Os02g0733900 | LOC_Os02g50110 | 2:30597807 | A | T | upstream |  |  |  |
| Os02g0733900 | LOC_Os02g50110 | 2:30597898 | A | G | upstream |  |  |  |
| Os02g0733900 | LOC_Os02g50110 | 2:30598318 | T | C | upstream |  |  |  |
| Os02g0733900 | LOC_Os02g50110 | 2:30598327 | C | G | upstream |  |  |  |
| Os02g0733900 | LOC_Os02g50110 | 2:30598485 | C | A | upstream |  |  |  |
| Os02g0733900 | LOC_Os02g50110 | 2:30598523 | G | A | upstream |  |  |  |
| Os02g0733900 | LOC_Os02g50110 | 2:30598534 | C | T | upstream |  |  |  |
| Os02g0733900 | LOC_Os02g50110 | 2:30599468 | T | G | upstream |  |  |  |
| Os02g0733900 | LOC_Os02g50110 | 2:30599497 | T | C | upstream |  |  |  |
| Os02g0733900 | LOC_Os02g50110 | 2:30599572 | G | T | upstream |  |  |  |
| Os02g0733900 | LOC_Os02g50110 | 2:30599584 | G | C | upstream |  |  |  |
| Os02g0733900 | LOC_Os02g50110 | 2:30599620 | T | A | upstream |  |  |  |
| Os02g0733900 | LOC_Os02g50110 | 2:30599803 | G | A | upstream |  |  |  |
| Os02g0733900 | LOC_Os02g50110 | 2:30599913 | A | T | upstream |  |  |  |
| Os02g0733900 | LOC_Os02g50110 | 2:30600012 | G | A | upstream |  |  |  |
| Os02g0733900 | LOC_Os02g50110 | 2:30600108 | C | T | upstream |  |  |  |
| Os02g0733900 | LOC_Os02g50110 | 2:30600114 | T | C | upstream |  |  |  |
| Os02g0733900 | LOC_Os02g50110 | 2:30600336 | G | T | upstream |  |  |  |
| Os02g0733900 | LOC_Os02g50110 | 2:30600353 | T | A | upstream |  |  |  |
| Os02g0733900 | LOC_Os02g50110 | 2:30600809 | C | T | upstream |  |  |  |
| Os02g0733900 | LOC_Os02g50110 | 2:30600863 | T | A | upstream |  |  |  |
| Os02g0733900 | LOC_Os02g50110 | 2:30600885 | C | A | upstream |  |  |  |
| Os02g0733900 | LOC_Os02g50110 | 2:30600946 | T | C | upstream |  |  |  |
| Os02g0733900 | LOC_Os02g50110 | 2:30601103 | C | T | upstream |  |  |  |
| Os02g0733900 | LOC_Os02g50110 | 2:30601119 | G | T | upstream |  |  |  |
| Os02g0733900 | LOC_Os02g50110 | 2:30601156 | A | G | upstream |  |  |  |
| Os02g0733900 | LOC_Os02g50110 | 2:30601157 | C | T | upstream |  |  |  |
| Os02g0733900 | LOC_Os02g50110 | 2:30601203 | G | A | upstream |  |  |  |
| Os02g0733900 | LOC_Os02g50110 | 2:30601226 | C | T | upstream |  |  |  |
| Os02g0733900 | LOC_Os02g50110 | 2:30601248 | A | G | upstream |  |  |  |
| Os02g0734101 | None | 2:30617526 | C | T | upstream |  |  |  |
| Os02g0734101 | None | 2:30617639 | T | C | upstream |  |  |  |
| Os02g0734101 | None | 2:30617687 | A | G | upstream |  |  |  |
| Os02g0734101 | None | 2:30617707 | G | A | upstream |  |  |  |
| Os02g0734101 | None | 2:30617780 | G | A | upstream |  |  |  |
| Os02g0734101 | None | 2:30617942 | G | A | upstream |  |  |  |
| Os02g0734101 | None | 2:30617958 | C | G | upstream |  |  |  |
| Os02g0734101 | None | 2:30618006 | A | G | upstream |  |  |  |
| Os02g0734101 | None | 2:30618049 | T | G | upstream |  |  |  |
| Os02g0734101 | None | 2:30618053 | A | C | upstream |  |  |  |
| Os02g0734101 | None | 2:30618125 | C | T | upstream |  |  |  |
| Os02g0734101 | None | 2:30618205 | G | A | upstream |  |  |  |
| Os02g0734101 | None | 2:30618376 | C | T | upstream |  |  |  |
| Os02g0734101 | None | 2:30618410 | C | T | upstream |  |  |  |
| Os02g0734101 | None | 2:30618948 | T | A | upstream |  |  |  |
| Os02g0734101 | None | 2:30618990 | C | T | upstream |  |  |  |
| Os02g0734101 | None | 2:30619011 | G | T | upstream |  |  |  |
| Os02g0734101 | None | 2:30619866 | C | G | upstream |  |  |  |
| Os02g0734101 | None | 2:30619902 | A | C | upstream |  |  |  |
| Os02g0734101 | None | 2:30620061 | T | G | upstream |  | 1.49E-07 |  |
| Os02g0734101 | None | 2:30620280 | G | T | upstream |  |  |  |
| Os02g0734101 | None | 2:30620356 | C | A | upstream |  |  |  |
| Os02g0734101 | None | 2:30621209 | G | T | upstream |  |  |  |
| Os02g0734101 | None | 2:30621225 | G | T | upstream |  |  |  |
| Os02g0734101 | None | 2:30621229 | C | A | upstream |  |  |  |
| Os02g0734101 | None | 2:30621356 | T | A | upstream |  |  |  |
| Os02g0734101 | None | 2:30621427 | T | A | upstream |  |  |  |
| Os02g0734101 | None | 2:30621455 | G | A | upstream |  |  |  |
| Os02g0734101 | None | 2:30621647 | A | G | upstream |  |  |  |
| Os02g0734101 | None | 2:30622068 | G | A | upstream |  |  |  |
| Os02g0734101 | None | 2:30622570 | A | T | 3_prime_UTR |  |  |  |
| Os02g0734101 | None | 2:30622583 | G | C | 3_prime_UTR |  |  |  |
| Os02g0734101 | None | 2:30622618 | T | C | 3_prime_UTR |  |  |  |
| Os02g0734101 | None | 2:30622642 | C | T | 3_prime_UTR |  |  |  |
| Os02g0734101 | None | 2:30622707 | A | G | 3_prime_UTR |  |  |  |
| Os02g0734101 | None | 2:30622839 | G | C | 3_prime_UTR |  |  |  |
| Os02g0734101 | None | 2:30623392 | A | G | upstream |  |  |  |
| Os02g0734300 | LOC_Os02g50130 | 2:30623462 | G | T | upstream |  |  |  |
| Os02g0734300 | LOC_Os02g50130 | 2:30623582 | C | T | upstream |  |  |  |
| Os02g0734300 | LOC_Os02g50130 | 2:30623635 | T | G | upstream |  |  |  |
| Os02g0734300 | LOC_Os02g50130 | 2:30623744 | G | A | upstream |  |  |  |
| Os02g0734300 | LOC_Os02g50130 | 2:30623971 | T | C | upstream |  |  |  |
| Os02g0734300 | LOC_Os02g50130 | 2:30624043 | G | A | upstream |  |  |  |
| Os02g0734300 | LOC_Os02g50130 | 2:30624097 | C | G | upstream |  |  |  |
| Os02g0734300 | LOC_Os02g50130 | 2:30624187 | A | T | upstream |  |  |  |
| Os02g0734300 | LOC_Os02g50130 | 2:30624340 | A | G | upstream |  |  |  |
| Os02g0734300 | LOC_Os02g50130 | 2:30624422 | C | T | upstream |  |  |  |
| Os02g0734300 | LOC_Os02g50130 | 2:30624531 | C | A | upstream |  |  |  |
| Os02g0734300 | LOC_Os02g50130 | 2:30624573 | G | T | upstream |  |  |  |
| Os02g0734300 | LOC_Os02g50130 | 2:30624578 | G | A | upstream |  |  |  |
| Os02g0734300 | LOC_Os02g50130 | 2:30624619 | G | A | upstream |  |  |  |
| Os02g0734300 | LOC_Os02g50130 | 2:30624715 | G | A | upstream |  |  |  |
| Os02g0734300 | LOC_Os02g50130 | 2:30624724 | T | C | upstream |  |  |  |
| Os02g0734300 | LOC_Os02g50130 | 2:30624860 | G | A | upstream |  |  |  |
| Os02g0734300 | LOC_Os02g50130 | 2:30624952 | G | A | upstream |  |  |  |
| Os02g0734300 | LOC_Os02g50130 | 2:30625064 | C | T | upstream |  |  |  |
| Os02g0734300 | LOC_Os02g50130 | 2:30625095 | G | A | upstream |  |  |  |
| Os02g0734300 | LOC_Os02g50130 | 2:30625103 | G | A | upstream |  |  |  |
| Os02g0734300 | LOC_Os02g50130 | 2:30625364 | G | A | upstream |  |  |  |
| Os02g0734300 | LOC_Os02g50130 | 2:30625567 | C | T | upstream |  |  |  |
| Os02g0734300 | LOC_Os02g50130 | 2:30625589 | T | C | upstream |  |  |  |
| Os02g0734300 | LOC_Os02g50130 | 2:30625639 | C | T | upstream |  |  |  |
| Os02g0734300 | LOC_Os02g50130 | 2:30626275 | G | A | exonic | synonymous |  |  |
| Os02g0734300 | LOC_Os02g50130 | 2:30626778 | C | T | 3_prime_UTR |  |  |  |
| Os02g0734300 | LOC_Os02g50130 | 2:30626807 | A | G | 3_prime_UTR |  |  |  |
| Os02g0734300 | LOC_Os02g50130 | 2:30626846 | T | C | 3_prime_UTR |  |  |  |
| Os02g0734300 | LOC_Os02g50130 | 2:30626897 | C | T | upstream |  |  |  |
| Os02g0734400 | LOC_Os02g50140 | 2:30626914 | G | A | upstream |  |  |  |
| Os02g0734400 | LOC_Os02g50140 | 2:30627681 | C | A | upstream |  |  |  |
| Os02g0734400 | LOC_Os02g50140 | 2:30628323 | C | T | upstream |  |  |  |
| Os02g0734400 | LOC_Os02g50140 | 2:30628324 | T | C | upstream |  |  |  |
| Os02g0734400 | LOC_Os02g50140 | 2:30628424 | C | T | upstream |  |  |  |
| Os02g0734400 | LOC_Os02g50140 | 2:30628494 | C | T | upstream |  |  |  |
| Os02g0734400 | LOC_Os02g50140 | 2:30628548 | T | C | upstream |  |  |  |
| Os02g0734400 | LOC_Os02g50140 | 2:30628551 | T | C | upstream |  |  |  |
| Os02g0734400 | LOC_Os02g50140 | 2:30628923 | G | A | upstream |  |  |  |
| Os02g0734400 | LOC_Os02g50140 | 2:30629098 | C | A | upstream |  |  |  |
| Os02g0734400 | LOC_Os02g50140 | 2:30629117 | C | A | upstream |  |  |  |
| Os02g0734400 | LOC_Os02g50140 | 2:30629131 | C | T | upstream |  |  |  |
| Os02g0734400 | LOC_Os02g50140 | 2:30629186 | C | G | upstream |  |  |  |
| Os02g0734400 | LOC_Os02g50140 | 2:30629230 | G | A | upstream |  |  |  |
| Os02g0734400 | LOC_Os02g50140 | 2:30629365 | C | T | upstream |  |  |  |
| Os02g0734400 | LOC_Os02g50140 | 2:30629383 | G | A | upstream |  |  |  |
| Os02g0734400 | LOC_Os02g50140 | 2:30629452 | C | G | upstream |  |  |  |
| Os02g0734400 | LOC_Os02g50140 | 2:30629568 | C | T | upstream |  |  |  |
| Os02g0734400 | LOC_Os02g50140 | 2:30629601 | A | G | upstream |  |  |  |
| Os02g0734400 | LOC_Os02g50140 | 2:30629797 | A | C | upstream |  |  |  |
| Os02g0734400 | LOC_Os02g50140 | 2:30629861 | G | A | upstream |  |  |  |
| Os02g0734400 | LOC_Os02g50140 | 2:30629885 | C | T | upstream |  |  |  |
| Os02g0734400 | LOC_Os02g50140 | 2:30629978 | C | A | upstream |  |  |  |
| Os02g0734400 | LOC_Os02g50140 | 2:30629987 | G | A | upstream |  |  |  |
| Os02g0734400 | LOC_Os02g50140 | 2:30630192 | G | A | upstream |  | 1.89E-06 |  |
| Os02g0734400 | LOC_Os02g50140 | 2:30630292 | G | A | upstream |  |  |  |
| Os02g0734400 | LOC_Os02g50140 | 2:30630323 | A | G | upstream |  |  |  |
| Os02g0734400 | LOC_Os02g50140 | 2:30630354 | G | A | upstream |  |  |  |
| Os02g0734400 | LOC_Os02g50140 | 2:30630450 | T | C | upstream |  |  |  |
| Os02g0734400 | LOC_Os02g50140 | 2:30630462 | A | G | upstream |  |  |  |
| Os02g0734400 | LOC_Os02g50140 | 2:30630533 | T | C | upstream |  |  |  |
| Os02g0734400 | LOC_Os02g50140 | 2:30630603 | T | C | upstream |  |  |  |
| Os02g0734400 | LOC_Os02g50140 | 2:30630922 | G | A | upstream |  |  |  |
| Os02g0734400 | LOC_Os02g50140 | 2:30631259 | G | A | upstream |  |  |  |
| Os02g0734400 | LOC_Os02g50140 | 2:30631654 | T | C | intron |  |  |  |
| Os02g0734400 | LOC_Os02g50140 | 2:30631898 | C | A | exonic | nonsynonymous |  |  |
| Os02g0734400 | LOC_Os02g50140 | 2:30632488 | A | T | upstream |  |  |  |
| Os02g0734400 | LOC_Os02g50140 | 2:30636924 | G | A | downstream |  |  |  |
| Os02g0734400 | LOC_Os02g50140 | 2:30636933 | A | T | downstream |  |  |  |
| Os02g0734400 | LOC_Os02g50140 | 2:30637135 | A | G | downstream |  |  |  |
| Os02g0734400 | LOC_Os02g50140 | 2:30637142 | C | T | downstream |  |  |  |
| Os02g0734400 | LOC_Os02g50140 | 2:30637263 | T | C | downstream |  |  |  |
| Os02g0734400 | LOC_Os02g50140 | 2:30637264 | A | G | downstream |  |  |  |
| Os02g0734400 | LOC_Os02g50140 | 2:30637488 | G | T | intron |  |  |  |
| Os02g0734400 | LOC_Os02g50140 | 2:30637507 | G | A | upstream |  |  |  |
| Os02g0734500 | LOC_Os02g50150 | 2:30632787 | A | G | upstream |  |  |  |
| Os02g0734500 | LOC_Os02g50150 | 2:30633013 | C | T | upstream |  |  |  |
| Os02g0734500 | LOC_Os02g50150 | 2:30633034 | A | T | upstream |  |  |  |
| Os02g0734500 | LOC_Os02g50150 | 2:30633100 | C | T | upstream |  |  |  |
| Os02g0734500 | LOC_Os02g50150 | 2:30633154 | C | A | upstream |  |  |  |
| Os02g0734500 | LOC_Os02g50150 | 2:30633407 | G | T | upstream |  |  |  |
| Os02g0734500 | LOC_Os02g50150 | 2:30633481 | A | G | upstream |  |  |  |
| Os02g0734500 | LOC_Os02g50150 | 2:30633536 | C | T | upstream |  |  |  |
| Os02g0734500 | LOC_Os02g50150 | 2:30633617 | C | A | upstream |  |  |  |
| Os02g0734500 | LOC_Os02g50150 | 2:30633623 | C | G | upstream |  |  |  |
| Os02g0734500 | LOC_Os02g50150 | 2:30633693 | G | A | upstream |  |  |  |
| Os02g0734500 | LOC_Os02g50150 | 2:30633748 | C | G | upstream |  |  |  |
| Os02g0734500 | LOC_Os02g50150 | 2:30633834 | C | A | upstream |  |  |  |
| Os02g0734500 | LOC_Os02g50150 | 2:30633930 | G | A | upstream |  |  |  |
| Os02g0734500 | LOC_Os02g50150 | 2:30634954 | G | C | upstream |  |  |  |
| Os02g0734500 | LOC_Os02g50150 | 2:30635350 | A | G | upstream |  |  |  |
| Os02g0734500 | LOC_Os02g50150 | 2:30635570 | C | T | upstream |  |  |  |
| Os02g0734500 | LOC_Os02g50150 | 2:30635597 | A | G | upstream |  |  |  |
| Os02g0734500 | LOC_Os02g50150 | 2:30635640 | G | A | upstream |  |  |  |
| Os02g0734500 | LOC_Os02g50150 | 2:30635742 | C | A | upstream |  |  |  |
| Os02g0734500 | LOC_Os02g50150 | 2:30635744 | G | A | upstream |  |  |  |
| Os02g0734500 | LOC_Os02g50150 | 2:30635841 | C | T | upstream |  |  |  |
| Os02g0734500 | LOC_Os02g50150 | 2:30636110 | G | A | 5_prime_UTR |  |  |  |
| Os02g0734500 | LOC_Os02g50150 | 2:30636146 | C | T | exonic | nonsynonymous |  |  |
| Os02g0734500 | LOC_Os02g50150 | 2:30636825 | A | T | downstream |  |  |  |
| Os02g0734500 | LOC_Os02g50150 | 2:30636844 | C | T | downstream |  |  |  |
| Os02g0734500 | LOC_Os02g50150 | 2:30637606 | A | G | upstream |  |  |  |
| Os02g0734500 | LOC_Os02g50150 | 2:30638212 | G | A | upstream |  |  |  |
| Os02g0734500 | LOC_Os02g50150 | 2:30643227 | T | C | downstream |  |  |  |
| Os02g0734500 | LOC_Os02g50150 | 2:30643802 | C | T | intron |  |  |  |
| Os02g0734600 | LOC_Os02g50174 | 2:30637908 | A | C | exonic | synonymous |  |  |
| Os02g0734600 | LOC_Os02g50174 | 2:30638094 | T | C | upstream |  |  |  |
| Os02g0734600 | LOC_Os02g50174 | 2:30638222 | A | G | upstream |  |  |  |
| Os02g0734600 | LOC_Os02g50174 | 2:30641238 | G | C | upstream |  |  |  |
| Os02g0734600 | LOC_Os02g50174 | 2:30641306 | C | G | upstream |  |  |  |
| Os02g0734600 | LOC_Os02g50174 | 2:30641557 | A | G | upstream |  |  |  |
| Os02g0734600 | LOC_Os02g50174 | 2:30641843 | G | T | upstream |  |  |  |
| Os02g0734600 | LOC_Os02g50174 | 2:30642047 | T | A | upstream |  |  |  |
| Os02g0734600 | LOC_Os02g50174 | 2:30642049 | G | T | upstream |  |  |  |
| Os02g0734600 | LOC_Os02g50174 | 2:30642164 | T | C | upstream |  |  |  |
| Os02g0734600 | LOC_Os02g50174 | 2:30642224 | A | G | upstream |  |  |  |
| Os02g0734600 | LOC_Os02g50174 | 2:30642448 | C | T | 5_prime_UTR |  |  |  |
| Os02g0734600 | LOC_Os02g50174 | 2:30642451 | A | T | 5_prime_UTR |  |  |  |
| Os02g0734600 | LOC_Os02g50174 | 2:30642775 | G | A | exonic | nonsynonymous |  |  |
| Os02g0734600 | LOC_Os02g50174 | 2:30642950 | T | A | downstream |  |  |  |
| Os02g0734600 | LOC_Os02g50174 | 2:30643041 | T | G | exonic | nonsynonymous |  |  |
| Os02g0734600 | LOC_Os02g50174 | 2:30643780 | C | T | intron |  |  |  |
| Os02g0734600 | LOC_Os02g50174 | 2:30644119 | T | A | intron |  |  |  |
| Os02g0734600 | LOC_Os02g50174 | 2:30644143 | T | C | intron |  |  |  |
| Os02g0734600 | LOC_Os02g50174 | 2:30644226 | A | G | intron |  |  |  |
| Os02g0734600 | LOC_Os02g50174 | 2:30644266 | C | T | intron |  |  |  |
| Os02g0734600 | LOC_Os02g50174 | 2:30648615 | C | T | upstream |  |  |  |
| Os02g0734600 | LOC_Os02g50174 | 2:30648704 | G | A | upstream |  |  |  |
| Os02g0734600 | LOC_Os02g50174 | 2:30648714 | G | C | upstream |  |  |  |
| Os02g0734600 | LOC_Os02g50174 | 2:30648718 | C | T | upstream |  |  |  |
| Os02g0734600 | LOC_Os02g50174 | 2:30648944 | T | C | upstream |  |  |  |
| Os02g0734600 | LOC_Os02g50174 | 2:30649098 | T | C | upstream |  |  |  |
| Os02g0734600 | LOC_Os02g50174 | 2:30649101 | T | C | upstream |  |  |  |
| Os02g0734600 | LOC_Os02g50174 | 2:30649107 | T | G | upstream |  |  |  |
| Os02g0734600 | LOC_Os02g50174 | 2:30649198 | G | A | upstream |  |  |  |
| Os02g0734600 | LOC_Os02g50174 | 2:30649257 | A | C | upstream |  |  |  |
| Os02g0734600 | LOC_Os02g50174 | 2:30649655 | T | C | upstream |  |  |  |
| Os02g0734600 | LOC_Os02g50174 | 2:30649719 | C | T | upstream |  |  |  |
| Os02g0734600 | LOC_Os02g50174 | 2:30649783 | G | A | upstream |  |  |  |
| Os02g0734600 | LOC_Os02g50174 | 2:30649818 | C | T | upstream |  |  |  |
