# Supplementary material for: OsSYL2 AA, an allele identified by gene‐based association, increases style length in rice (Oryza sativa L.)
Source: Plant J. 2020 Oct 30;104(6):1491–503. doi: 10.1111/tpj.15013 (PMC7821000; doi:10.1111/tpj.15013)
Supplement: Supplementary file 9 — Table S8. Candidate gene annotation in the linkage disequilibrium region 30.45–30.65 Mb associated with style length trait. [file TPJ-104-1491-s009.docx]

**Table S8.** Candidate gene annotation in the LD region 30.45-30.65 Mb associated with style length trait.

| Number | RAP ID | MSU ID | Position | Annotation |
| --- | --- | --- | --- | --- |
| 1 | Os02g0730775 | None | 30,444,816-30,447,866 | hypothetical protein |
| 2 | Os02g0730900 | LOC_Os02g49830 | 30,448,000-30,449,510 | pentatricopeptide repeat domain containing protein |
| 3 | Os02g0731050 | None | 30,449,574-30,451,465 | non-protein coding transcript |
| 4 | Os02g0731200 | LOC_Os02g49840 | 30,456,666-30,462,759 | MADS-box transcription factor, Cold tolerance, Control of tillering |
| 5 | Os02g0731300 | None | 30,459,392-30,464,135 | non-protein coding transcript |
| 6 | Os02g0731400 | LOC_Os02g49850 | 30,467,578-30,468,413 | plantacyanin |
| 7 | Os02g0731500 | LOC_Os02g49860 | 30,468,783-30,470,102 | AWPM-19-like membrane family protein |
| 8 | Os02g0731600 | LOC_Os02g49870 | 30,472,233-30,473,053 | threonine endopeptidase |
| 9 | Os02g0731700 | LOC_Os02g49880 | 30,473,739-30,475,800 | grain number, plant height, and heading date2", CONSTANS-like 1 protein |
| 10 | Os02g0731900 | LOC_Os02g49920 | 30,500,236-30,502,286 | 3-ketoacyl-CoA synthase |
| 11 | Os02g0732200 | LOC_Os02g49950 | 30,518,062-30,522,141 | armadillo-like helical domain containing protein |
| 12 | Os02g0732250 | None | 30,520,916-30,522,141 | hypothetical protein |
| 13 | Os02g0732300 | LOC_Os02g49960 | 30,524,531-30,526,667 | conserved hypothetical protein |
| 14 | Os02g0732350 | None | 30,524,662-30,525,269 | hypothetical protein |
| 15 | Os02g0732400 | LOC_Os02g49970 | 30,528,645-30,530,536 | tify domain containing protein |
| 16 | Os02g0732500 | LOC_Os02g49980 | 30,533,422-30,536,326 | ADP-ribosylation factor |
| 17 | Os02g0732600 | LOC_Os02g49986 | 30,539,853-30,541,113 | MYB family transcription factor |
| 18 | Os02g0732700 | LOC_Os02g49992 | 30,542,197-30,553,568 | mediator complex, subunit Med23 domain containing protein |
| 19 | Os02g0732800 | LOC_Os02g50000 | 30,554,630-30,556,339 | GDSL-like lipase/acylhydrolase |
| 20 | Os02g0732900 | LOC_Os02g50010 | 30,557,558-30,560,948 | protein of unknown function DUF794, plant family protein |
| 21 | Os02g0733001 | LOC_Os02g50020 | 30,561,276-30,563,576 | microtubule motor |
| 22 | Os02g0733166 | None | 30,567,233-30,567,808 | hypothetical conserved gene |
| 23 | Os02g0733200 | None | 30,567,356-30,569,976 | conserved hypothetical protein |
| 24 | Os02g0733300 | LOC_Os02g50040 | 30,573,454-30,577,260 | Endo-beta-1,4-glucanase precursor |
| 25 | Os02g0733400 | LOC_Os02g50050 | 30,577,640-30,578,383 | hypothetical conserved gene |
| 26 | Os02g0733500 | LOC_Os02g50060 | 30,579,928-30,580,869 | EF-Hand type domain containing protein |
| 27 | Os02g0733800 | LOC_Os02g50100 | 30,593,495-30,596,146 | SET domain containing protein |
| 28 | Os02g0733900 | LOC_Os02g50110 | 30,596,400-30,597,001 | hypothetical conserved gene |
| 29 | Os02g0734101 | None | 30,622,333-30,622,900 | hypothetical conserved gene |
| 30 | Os02g0734300 | LOC_Os02g50130 | 30,625,668-30,626,865 | NUDIX family |
| 31 | Os02g0734400 | LOC_Os02g50140 | 30,631,324-30,632,669 | ABA-induced protein |
| 32 | Os02g0734500 | LOC_Os02g50150 | 30,636,023-30,638,501 | ABA-induced protein |
| 33 | Os02g0734600 | LOC_Os02g50174 | 30,642,392-30,654,596 | caleosin related protein |
