# Supplementary material for: OsSYL2 AA, an allele identified by gene‐based association, increases style length in rice (Oryza sativa L.)
Source: Plant J. 2020 Oct 30;104(6):1491–503. doi: 10.1111/tpj.15013 (PMC7821000; doi:10.1111/tpj.15013)
Supplement: Supplementary file 10 — Table S9. Correlation coefficients between grain length trait and stigma characteristics. [file TPJ-104-1491-s010.docx]

**Table S9.** Correlation coefficients between grain length and stigma characteristics.

|  | GL | TSSL | STL | SYL |
| --- | --- | --- | --- | --- |
| GL | 1 |  |  |  |
| TSSL | 0.61^**^ | 1 |  |  |
| STL | 0.44^**^ | 0.88^**^ | 1 |  |
| SYL | 0.58^**^ | 0.71^**^ | 0.30^**^ | 1 |

^**^ represents significant at α = 0.01 probability level.
