# Supplementary material for: OsSYL2 AA, an allele identified by gene‐based association, increases style length in rice (Oryza sativa L.)
Source: Plant J. 2020 Oct 30;104(6):1491–503. doi: 10.1111/tpj.15013 (PMC7821000; doi:10.1111/tpj.15013)
Supplement: Supplementary file 11 — Table S10. The results of significantly associated single‐nucleotide polymorphism loci detected in this study overlapped with the quantitative trait loci/genes of rice stigma characteristics reported previously. [file TPJ-104-1491-s011.docx]

**Table S10.** The results of significantly associated SNP loci detected in this study overlapped with the QTLs/Genes of rice stigma characteristics reported previously.

| Traits | Chromosome | SNP site | Local LD | Known gene | Known QTL/ association locus | Flanking region | Reference |
| --- | --- | --- | --- | --- | --- | --- | --- |
| STL | 7 | 24396164 | 24294065-24763963 | *GL7* |  | 24,664,328-24,669,321 | Wang et al. 2015 |
|  |  |  |  |  |  |  |  |
| SYL | 3 | 16690429 | 16663167-16915445 |  | *qSYL3* | 16,723,270 | Zhou et al. 2017 |
|  | 3 | 16692834 | 16663167-16915445 |  | *qSYL3* | 16,723,270 | Zhou et al. 2017 |
|  | 3 | 16708049 | 16663167-16915445 |  | *qSYL3* | 16,723,270 | Zhou et al. 2017 |
|  | 3 | 16733441 | 16663167-16915445 | *GS3* |  | 16,729,501-16,735,109 | Fan et al. 2006 |
|  | 3 | 16881568 | 16663167-16915445 |  | *qSYL3* | 16,723,270 | Zhou et al. 2017 |
|  | 6 | 26598751 | 26398744-26848690 |  | *qSYL6* | 26,161,226-26,728,508 | Uga et al. 2003 |
|  |  |  |  |  |  |  |  |
| TSSL | 3 | 16686373 | 16663167-16931334 |  | *qSYL3* | 16,723,270 | Zhou et al. 2017 |
|  | 3 | 16691998 | 16663167-16940264 |  | *qSYL3* | 16,723,270 | Zhou et al. 2017 |
|  | 3 | 16720463 | 16663167-16970433 |  | *qSYL3* | 16,723,270 | Zhou et al. 2017 |
|  | 3 | 16733441 | 16663167-16970433 | *GS3* |  |  | Fan et al. 2006 |
|  | 3 | 16878104 | 16663167-17020970 |  | *qSYL3* | 16,723,270 | Zhou et al. 2017 |
|  | 3 | 17019509 | 16777275-17020970 |  | *qSYL3* | 16,723,270 | Zhou et al. 2017 |

STL, stigma length; SYL, style length; TSSL, the sum of stigma and style length; GS3, Grain size 3, *Os03g0407400*; GL7, Grain length, *Os07g0603300*.
