# Supplementary material for: OsSYL2 AA, an allele identified by gene‐based association, increases style length in rice (Oryza sativa L.)
Source: Plant J. 2020 Oct 30;104(6):1491–503. doi: 10.1111/tpj.15013 (PMC7821000; doi:10.1111/tpj.15013)
Supplement: Supplementary file 12 — Table S11. Primers used in this study. [file TPJ-104-1491-s012.docx]

**Table S11.** Primers used in this study.

| Primer name | Forward primers (5’-3’) | Reverse primers (5’-3’) |
| --- | --- | --- |
| Primers for quantitative real-time RT-PCR: | | |
| RT1-18S | GAGATGGGTAGGGACGTGGAT | TGGTACGTCTCGTCCACCTT |
| RT2-*Os02g0733900* | GCACGTCCATCACAAACTCC | AGTAGAGTAGTACCCTGGCCC |
| RT3-*Os03g0407400* | TCACCAGTTTGAGCCGAAGT | TCATTGTCGCCACCCTTCAA |
| Primers for plasmid construction: | | |
| cOsSYL2 | cagtGGTCTCacaactattttatcccctacaacgaat | cagtGGTCTCatacaacagaaccaagatataactttc |
| Primers for genotype identification: | | |
|  | GGTACAGTTCCGTCCACTCT | ATGGGTAGGGACGTGGATGC |
